# Aberrant splicing isoforms detected by full-length transcriptome sequencing as transcripts of potential neoantigens in non-small cell lung cancer

**DOI:** 10.1186/s13059-020-02240-8

**Published:** 2021-01-04

**Authors:** Miho Oka, Liu Xu, Toshihiro Suzuki, Toshiaki Yoshikawa, Hiromi Sakamoto, Hayato Uemura, Akiyasu C. Yoshizawa, Yutaka Suzuki, Tetsuya Nakatsura, Yasushi Ishihama, Ayako Suzuki, Masahide Seki

**Affiliations:** 1 Department of Computational Biology and Medical Sciences, Graduate School of Frontier Sciences, The University of Tokyo, Chiba, Japan; 2 Ono Pharmaceutical Co., Ltd., Ibaraki, Japan; 3 General Medical Education and Research Center, Teikyo University, Tokyo, Japan; 4 Division of Cancer Immunotherapy, Exploratory Oncology Research and Clinical Trial Center, National Cancer Center, Chiba, Japan; 5 Department of Clinical Genomics, National Cancer Center Research Institute, Tokyo, Japan; 6 Department of Molecular and Cellular BioAnalysis, Graduate School of Pharmaceutical Sciences, Kyoto University, Kyoto, Japan

**Keywords:** MinION, Lung cancer, Splicing isoform, Neoantigen

## Abstract

**Background:**

Long-read sequencing of full-length cDNAs enables the detection of structures of aberrant splicing isoforms in cancer cells. These isoforms are occasionally translated, presented by HLA molecules, and recognized as neoantigens. This study used a long-read sequencer (MinION) to construct a comprehensive catalog of aberrant splicing isoforms in non-small-cell lung cancers, by which novel isoforms and potential neoantigens are identified.

**Results:**

Full-length cDNA sequencing is performed using 22 cell lines, and a total of 2021 novel splicing isoforms are identified. The protein expression of some of these isoforms is then validated by proteome analysis. Ablations of a nonsense-mediated mRNA decay (NMD) factor, UPF1, and a splicing factor, SF3B1, are found to increase the proportion of aberrant transcripts. NetMHC evaluation of the binding affinities to each type of HLA molecule reveals that some of the isoforms potentially generate neoantigen candidates. We also identify aberrant splicing isoforms in seven non-small-cell lung cancer specimens. An enzyme-linked immune absorbent spot assay indicates that approximately half the peptide candidates have the potential to activate T cell responses through their interaction with HLA molecules. Finally, we estimate the number of isoforms in The Cancer Genome Atlas (TCGA) datasets by referring to the constructed catalog and found that disruption of NMD factors is significantly correlated with the number of splicing isoforms found in the TCGA-Lung Adenocarcinoma data collection.

**Conclusions:**

Our results indicate that long-read sequencing of full-length cDNAs is essential for the precise identification of aberrant transcript structures in cancer cells.

**Supplementary Information:**

The online version contains supplementary material available at 10.1186/s13059-020-02240-8.

## Background

In cancer cells, transcript regulations are disrupted at various steps, resulting in the accumulation of substantial numbers of aberrant transcripts [1]. In normal cells, these aberrant transcripts, even if transcribed, are subsequently degraded by the mRNA quality control system in a process known as nonsense-mediated mRNA decay (NMD). NMD factors scan each transcript and subject mRNA-harboring premature translation termination codons (PTCs) to degradation [2]. However, the NMD regulation mechanism is also frequently disrupted in cancer cells [3–5], allowing the aberrant transcripts to escape degradation. Therefore, accumulations of aberrant transcripts are frequently observed in cancer cells. For example, *UPF1*, a highly conserved core NMD factor, is frequently mutated in pancreatic adenosquamous carcinoma [4]. Disruption of the *UPF1* function causes the accumulation of aberrant transcripts in the tumor [6, 7]. The relationship between NMD and tumor immunity has been reported [8] and considered to be a therapeutic target for cancers in some conditions [9].

Aberrations are also caused by impaired gene expression of splicing-related factors [10]. Indeed, the relationship between the aberrations and disrupted splicing regulation has been reported for various cancer types. *U2AF1* and *SF3B1*, the core components of spliceosomes, are frequently mutated in cases of myelodysplastic syndrome (MDS) [11, 12] and several types of solid tumor [13, 14]. *BUD31* was reported as an *MYC*-synthetic lethal gene in mammary epithelial cells and was found to disrupt processing in precursor mRNA [15]. Further, some specific aberrant isoforms directly contribute to tumorigenesis and malignancy [16]. Thus, splicing factors have also been identified as therapeutic targets for cancer [17, 18].

Recently, it has been suggested that putative functionally neutral aberrant transcripts have the potential to serve as novel molecular markers for immune check point inhibitor (ICI) therapy, which uses ICIs such as nivolumab, pembrolizumab, and atezolizumab. For current ICI treatment, the tumor mutational burden (TMB), which is determined by the total number of nonsynonymous point mutations, has been frequently considered to be a biomarker to identify patients with expected effects [19–21]. These nonsynonymous mutations alter amino acids and produce novel cancer-specific neoantigens, some of which are subsequently expressed in the HLA molecule and recognized by T cell receptors, which activates cytotoxic T cells to kill the cancer cells. [22, 23] The assumption is that the higher the expression of neoantigens in cancer cells, the greater the potential for the cancer cell to be attacked by immune cells following ICI treatment.

Currently, massive parallel sequencing techniques, such as whole-exome sequencing and panel sequencing, have been employed to measure TMB in various conditions. Several studies have reported that the TMB correlates with the efficacy of ICI treatment, especially in melanoma patients [24]. However, it has been gradually revealed that the TMB may not be a perfect marker of ICIs in many types of cancer [25–27]. Recent studies have reported that transcripts that harbor frameshift mutations and aberrant splicing patterns also produce antigenic peptides under the presumed condition of disrupted NMD [28–31]. Under such circumstances, the aberrant transcripts may have no less important potential as biomarkers for ICIs.

To consider all the effects of mutations and aberrant isoforms, proper peptide sequences from full-length transcript structures are needed. However, it is difficult to precisely identify the complete structure and aberration patterns of a transcript from the fragmented reads produced by short-read RNA sequencing technology. Recently, long-read sequencing technology has enabled us to obtain full-length transcriptome profiles. Particularly, MinION, a nanopore-type sequencer from Oxford Nanopore Technologies, can produce full-length cDNA data, covering the entire transcript at sufficient sequencing depth. Recent papers have also shown that the long-read sequencing achieves approximately 90% accuracy [32], which is sufficient for the precise extraction of splicing patterns.

In this study, in combination with the long-read and short-read sequencing technologies, we attempt to construct a catalog of full-length transcript structures in a series of lung cancer cells. Based on this catalog, we examined whether the deduced full-length protein sequences with mutations and/or frameshift aberrations can serve as predictive markers for ICIs in addition to TMB. For this purpose, we started with a cell line panel of lung adenocarcinoma as the model. We then applied the developed method to clinical samples from Japanese non-small cell lung cancer (NSCLC) patients.

## Results

### Cataloging full-length transcript isoforms of lung cancer cell lines

MinION full-length DNA sequencing was performed for 22 NSCLC cell lines (Additional file 1: Table S1), in which characteristic genomic driver mutation patterns and transcriptomic profiles of lung cancers were collectively represented. The schematic representation of the analytical pipeline constructed for this study is shown in Additional file 12: Fig. S1A. An average of 3.5 million reads at an average read length of 1.6 kb were generated from each cell line (Additional file 12: Figs. S1B, S2 and Additional file 2: Table S2). The obtained full-length cDNA reads were mapped to the human genome by Minimap2 as previously reported [33]. To overcome the inaccuracy of MinION sequencing [32], low-quality and ambiguously aligned reads were filtered out (Additional file 2: Table S2). All splicing junctions were confirmed using the Illumina short-read RNA sequencing dataset of the same cell lines. The obtained splicing patterns were further compared to the current transcript models of the Reference Sequence (RefSeq) database (see “Materials and methods” for further details on the procedure for the identification and characterization of splicing junctions). Among the reads mapped to RefSeq transcripts, more than 50% of reads successfully covered full-length transcripts of up to 8000 genes (Additional file 12: Fig. S1C). For each gene, the number of reads per million (RPM) of MinION and transcripts per million (TPM) calculated from short-read sequencing were strongly correlated (Additional file 12: Fig. S1D and Fig. S3).

**Fig. 1:**
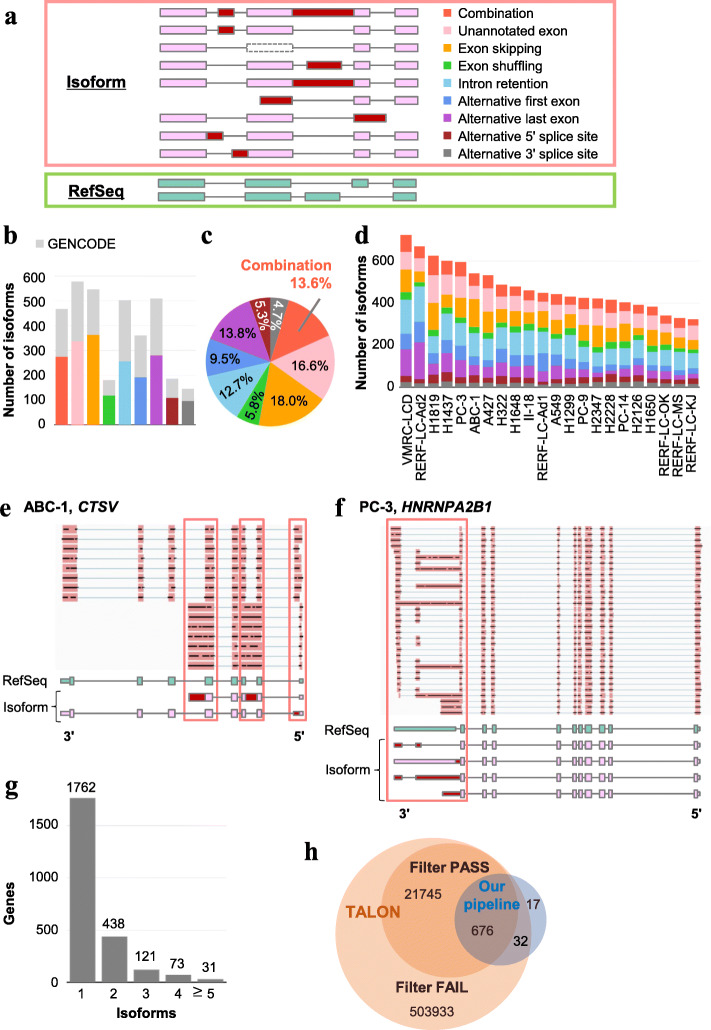
Identification of aberrant splicing isoforms in cell lines. **a** Classification of aberrant splicing events. Pink boxes represent constitutively spliced exons. Red and dotted-line boxes represent alternatively spliced exons. **b** The number of isoforms classified for each splicing event shown in **a**. Light gray bars indicate isoforms represented in GENCODE and other-colored bars indicate unannotated isoforms. **c** The proportion of splicing events in unannotated isoforms. The color key is shown in **a**. **d** The number of splicing isoforms and the proportion of each splicing event in cell lines. The color key is shown in **a**. **e** The full-length structure of the splicing isoform of *CTSV* in ABC-1. Some MinION reads show a combination pattern of intron retention, alternative last exon, and alternative 5′ splice site. **f** The full-length structure of splicing isoforms of *HNRNPA2B1* in PC-3. Some MinION reads indicate extensive alternative splicing within its 3′ UTR. **g** The number of isoform patterns per gene in the cell lines. **h** A comparison of the number of isoforms in VMRC-LCD derived from our pipeline and from the TALON pipeline (99% of the isoforms we detected were included in the TALON results)

We identified the complete exon-intron structures of transcript isoforms and classified them into the following types: unannotated exon, exon skipping, exon shuffling, intron retention, alternative first exon, alternative last exon, alternative 5′ splice site, and alternative 3′ splice site (Fig. 1a and Additional file 3: Table S3). As a result, we identified 3474 non-RefSeq isoforms from all the cell lines and named them putative “aberrant splicing isoforms,” hereinafter referred to as “isoforms.” Of these, 2021 isoforms harbored at least one splicing event that was not represented in the RefSeq or the GENCODE datasets (Fig. 1b). We also identified novel combinations of these splicing events on a single full-length transcript, which would have been difficult to detect via the fragmented reads of short-read sequencing data. Collectively, these patterns accounted for 13.6% of the unannotated isoforms (Fig. 1c).

The patterns and numbers for each of the NSCLC cell lines represent characteristic aberrant splicing events (Fig. 1d and Additional file 3: Table S3). The number of isoforms ranged from 323 to 725. The compositions of isoform patterns differed among the cell lines. For example, intron retention and alternative last exon composed the largest proportions of isoforms in RERF-LC-Ad2, while unannotated exon and exon skipping were characteristics in H1819. These results suggest the diversity of aberrant splicing isoform patterns even in cell lines. An example of a novel complicated combination isoform of splicing events is shown in Fig. 1e. *CTSV* in ABC-1 cells expressed two isoforms, one of which included the combination of alternative last exon and intron retention that occurred between exons 2 and 3.

Genes with multiple isoforms were also observed. For example, *HNRNPA2B1* showed four isoforms that contained alternative splicing events within 3′ UTR of PC-3 cells (Fig. 1f). This extensive accumulation of splicing events in the 3′ UTR of *HNRNPA2B1* was also observed in a previous study that performed a *UPF1* knockdown experiment in HeLa cells [34]. We identified 663 genes with multiple isoforms in total (Fig. 1g). These results reflect the great potential of long-read sequencing to comprehensively detect novel and complicated isoforms.

To validate of our computational pipeline, we compared the isoform patterns in VMRC-LCD cells detected by our pipeline and by the TALON software. The TALON software, which was launched by the ENCODE project [35], identifies novel isoforms using long-read sequencing datasets. As a result, 708 of 725 isoforms (98%) detected in our pipeline were also detected in TALON, and 676 of them (93%) successfully passed TALON’s filtering conditions (Fig. 1h). The 17 isoforms that could not be detected by TALON were supported by reads whose junctions were altered by the long-read error correction tool TranscriptClean [36], a part pf TALON pipeline. Although 21,745 isoforms were detected only in TALON, 99% were covered by reads that were filtered out by our previous filtering conditions (attributed to ambiguous alignments or low expression levels, data not shown). Our filtering conditions aimed to avoid false-positive detection and to keep higher expressed transcripts, which can be translated as neoantigens; therefore, our pipeline extracted more conservative isoform patterns than the isoform patterns extracted by the TALON pipeline.

To confirm the effect of repetitive element loci, which can lead to misalignment of the reads, we evaluated 5508 splice junctions detected in 22 cell lines. We extracted ± 50 bp regions around the splice sites and searched repetitive regions using RepeatMasker. As a result, 94.9% of these splicing sites did not overlap repetitive sequences (Additional File 12: Fig. S1E). This result suggests that novel splice sites were not particularly associated with the repetitive sequences.

Some gene-fusion events were reported to function as driver genes contributing to tumorigenicity in many cancers [37, 38]. To address this issue, we firstly manually inspected the presence or absence of the previously reported frequently “fused” genes. We successfully identified the EML4-ALK fusion transcript, as a driver fusion event, in H2228 (Additional file 12: Figs. S4A). In addition, we identified the ERGIC2-CHRNA6 transcript in H1437 (Additional file 12: Figs. S4B). This is a fusion event causing the frameshift, invoking a drastic amino acid change, thus may serve as an important source of a novel neoantigen. For these fusions, it is true that our previous studies have suggested their presences by the short-read sequencing [1, 39]. However, we firstly identified their complete transcript forms by using the MinION long reads in this study. Particularly interestingly for the ERGIC2-CHRNA6 fusion gene, we identified four alternative splice isoforms, consisting of an unannotated exon and an alternative last exon.

### Characterization of the detected isoforms

**Fig. 2:**
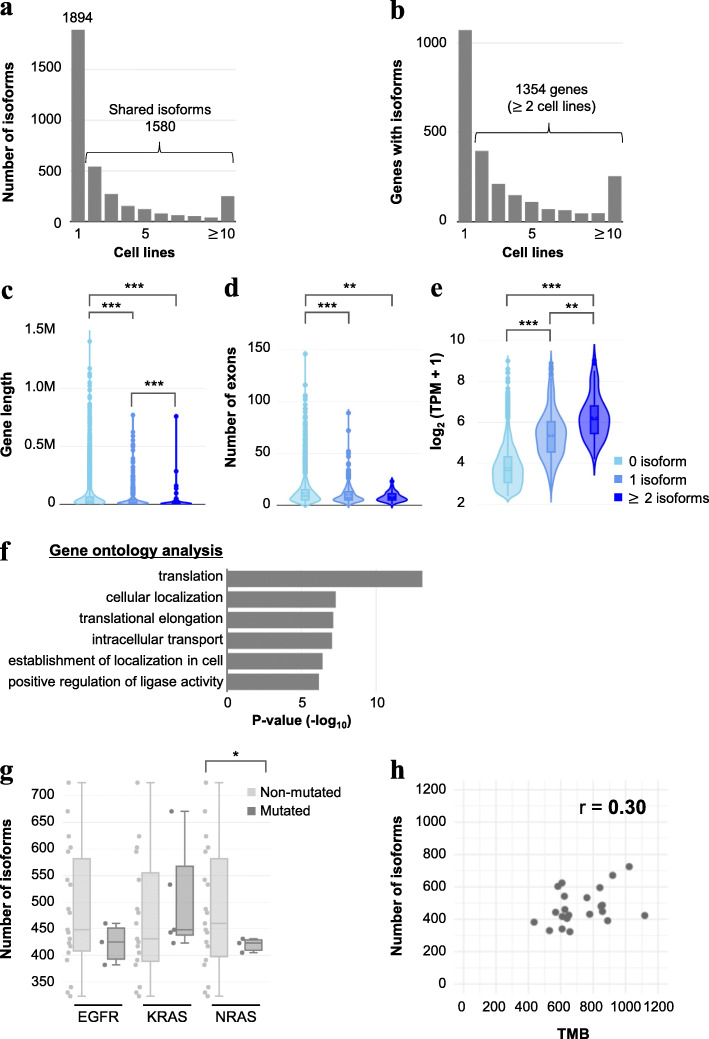
Characterization of isoform-enriched genes in cell lines. **a** The number of cell lines that expressed isoforms in common. **b** The number of cell lines that expressed genes with isoforms in common. **c**–**e** The distribution of lengths (**c**), exons (**d**), and expression level (**e**) of genes with isoforms in VMRC-LCD. Isoform-enriched genes showed tendencies of shorter length, fewer exons, and higher expression. **P < 0.01 and ***P < 0.001 (Kruskal–Wallis test and Dunn–Bonferroni’s post hoc test). **f** The results of the gene ontology enrichment analysis for genes with at least one isoform among cell lines. **g** Comparisons of the distribution of the number of isoforms in cell lines with or without the driver mutations *EGFR*, *KRAS*, and *NRAS*. *P < 0.05 (Welch’s *t*-test). **h** Comparison of the number of isoforms and TMB for each cell line. No significant correlation was observed (*r* = 0.30)

We further characterized the novel isoforms and found that 45% of the isoforms were shared among the cell lines, although 1894 isoforms were uniquely detected in each cell line (Fig. 2a). A total of 1354 genes harbored aberrant splicing isoforms in two or more cell lines (Fig. 2b and Additional file 12: Fig. S5).

To characterize isoform-enriched genes, we compared lengths, the number of exons, and expression levels of genes with/without isoforms. Genes harboring at least one isoform showed significantly shorter lengths and consisted of a smaller number of exons compared to those of genes without isoforms (Additional file 12: Figs. S6, S7, and S9); however, expression levels were found to be significantly higher in genes that harbored isoforms (Additional file 12: Figs. S8 and S9). We also found that isoform variety was associated with gene length and expression level. Several cell lines showed significant differences in gene length and expression level between genes with one isoform and those with two or more isoforms. For example, in VMRC-LCD, isoform-enriched genes tend to have shorter length and higher expression than genes with only one isoform (Fig. 2c–e).

To examine the relation between the expression levels and the detection probability, we subsampled sequencing reads of VMRC-LCD cells to 1/2, 1/5, 1/10, 1/50, and 1/100 (*n* = 100) and calculated the detection probability for each isoform. We divided these isoforms into three classes, High, Middle, and Low, for each of the three categories as below: (1) TPM of the gene (total gene expression), which was calculated from the short-read RNA sequencing data (Additional file 12 Fig. S9B); (2) isoform-reads ratio (isoform frequency within the gene): [the number of MinION reads covering the isoform pattern] / [the total number of MinION reads assigned to the gene] (Additional file 12 Fig. S9C); (3) isoform-reads (absolute count of the isoform): the number of MinION reads covering the isoform pattern (Additional file 12 Fig. S9D). As a result, we found that three classes of the isoforms showed similar detection probability in the categories of “TPM” and “isoform-reads ratio,” suggesting that the isoform detection was somewhat independent of these categories. On the other hand, “isoform-reads” showed significant differences among the classes for the detection probabilities, suggesting the low level isoforms were, indeed, difficult to be detected at low sequencing depth. However, at the sequencing depth conducted in this study, each case seems to have reached to a plateau to some extent. Also note that, on average, 360,000 mRNA molecules are estimated to exist in a single lung cancer cell [40]. Thus, one copy of mRNA per cell corresponds to 3 TPM. The minimum TPM of the gene for which at least one isoform was detected was 6 TPM in VMRC-LCD. These facts suggest that we were able to identify the isoforms to the very low expression level within a cell.

We also conducted a gene ontology analysis using genes with at least one isoform and found that RNA-binding proteins involved in the translation pathway were significantly enriched (Fig. 2f and Additional file 12: Fig. S10). This result is consistent with a previous study of clinical specimens of MDS [41]. The study reported that aberrant splicing events in patients with mutations of *SF3B1*, *U2AF1*, and *SRSF2* were enriched in mRNA metabolic processes, including translation [41]. Expressions of some of the ribosomal protein genes and splicing-related genes are generally regulated via alternative splicing events and thus may be vulnerable to alternative splicing [42, 43]. In general, ribosomal protein genes were found to be shorter and to have higher expression levels than other genes [44]. This feature may contribute to the tendency of isoform-enriched genes to have the shorter gene length and higher expression levels (Fig. 2c–e) that were observed in this study.

No significant association was found between the number of aberrant splicing isoforms and *EGFR*, *KRAS*, or *NRAS* driver mutations in the cell lines (Fig. 2g). Significantly, we found that the number of aberrant splicing isoforms showed poor correlation with the genomic TMB, which is a source of neoantigens and one of the known markers for ICI efficacy (*r* = 0.30, Fig. 2h). The aberrant splicing isoforms detected in this study also have the potential to be translated and presented as neoantigens; therefore, we expect further analysis of these splicing patterns to provide an independent measure to define cancer cells in addition to TMB.

### Biological validation of the aberrant splicing isoforms

**Fig. 3:**
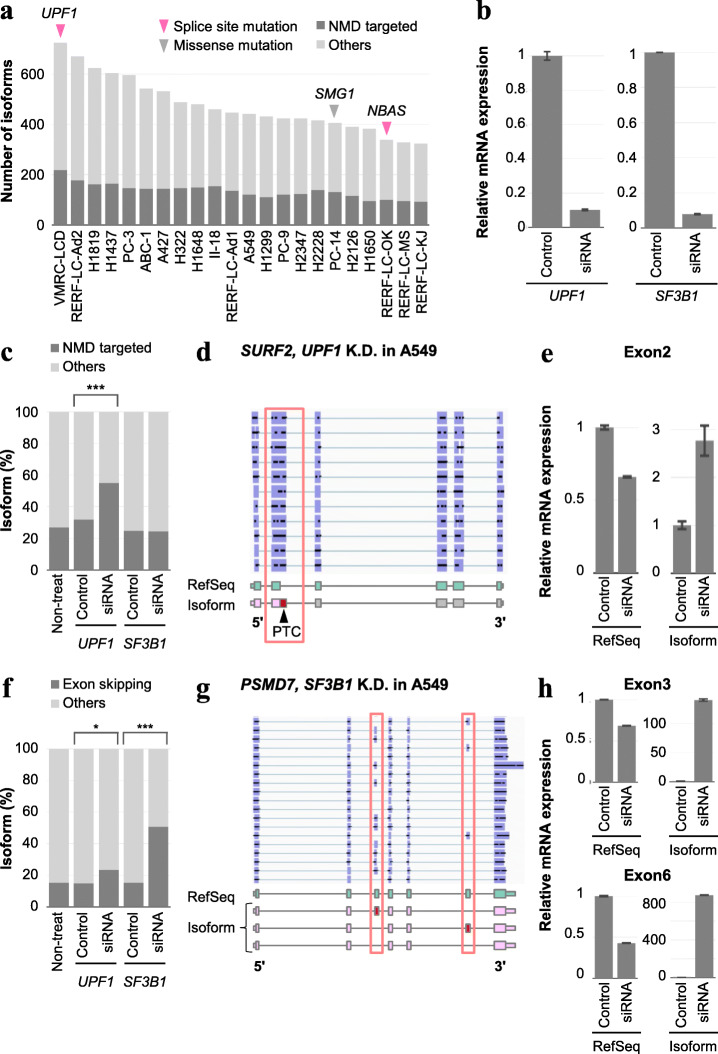
Causes of aberrant transcripts in cancer cells. **a** The number of PTC-containing splicing isoforms in each cell line are shown in gray. Arrows represent somatic mutations of NMD factors in each cell line. **b** The relative *UPF1* and *SF3B1* mRNA levels normalized to *GAPDH* levels in A549 cells at 72 h post-transfection (analyzed by RT-PCR). Error bars represent the standard error of the mean (SEM). **c** The proportion of PTC-containing isoforms after knockdown experiments in A549. The *UPF1*-depleted sample showed a significant increase in the proportion of PTC-containing isoforms. ***P < 0.001 (Fisher’s exact test). **d** The full-length structure of splicing isoforms of *SURF2* in *UPF1*-depleted A549. Some MinION reads showed an alternative 5′ splice site in exon 2 containing a PTC. **e** The relative *SURF2* mRNA levels of the RefSeq type and isoform in *UPF1*-depleted A549 cells. Each expression level was detected with primers flanking exon 2 and was normalized with a common region level shown in Additional file 12: Fig. S10C. Error bars represent the SEM. **f** The proportion of exon-skipping isoforms after knockdown experiments in A549. *SF3B1*- and *UPF1*-depleted samples showed significant increases in the proportion of exon-skipping isoforms. ***P < 0.001, *P < 0.05 (Fisher’s exact test). **g** The full-length structure of splicing isoforms of *PSMD7* in *SF3B1*-depleted A549. Some MinION reads showed a combination of skipping events at exons 3 and 6. **h** The relative *PSMD7* mRNA levels of the RefSeq type and isoform in *SF3B1*-depleted A549 cells. Each expression level was detected with primers flanking exons 3 and 6, then was normalized with a common region level (shown in Additional file 12: Fig. S10E). Error bars represent the SEM

Next, we investigated the potential causes of aberrant splicing isoforms in cancer cells. As we were able to analyze the isoforms as a form of full-length transcript, we counted aberrant isoforms containing PTCs, which would have been targeted by NMD. We found that ~ 30% of the aberrant isoforms contained PTCs (Fig. 3a and Additional file 3: Table S3). Indeed, when we examined the case of VMRC-LCD, which showed the highest number of aberrant splicing isoforms, we found this cell line harbored a splice site mutation in *UPF1*, which is a key NMD factor (Additional file 12: Fig. S11A). To more directly validate the possible cause of the accumulation of aberrant isoforms, we conducted an siRNA knockdown experiment for *UPF1* in A549 (Fig. 3b). We similarly analyzed the obtained full-length cDNA MinION reads in combination with the Illumina short-read RNA sequencing data. As expected, the proportion of NMD-targeted isoforms was significantly increased by the *UPF1* knockdown (Fig. 3c). For example, an intron-retained isoform in the *SURF2* gene was detected only in the *UPF1* knockdown cells despite this isoform containing a PTC and being potentially targeted by NMD (Fig. 3d). To validate the expression of this isoform specifically in *UPF1*-depleted cells, we perform RT-PCR with primers flanking the isoform-specific junction (Additional file 12: Fig. S11C). The alternative 5′ splice site in exon 2 of *SURF2* showed a two- to three-fold increase in response to the *UPF1* knockdown experiment (Fig. 3e). This increase was also detected as size differences among PCR products (Additional file 12: Fig. S11D).

In the previous study, four p-UPF1-binding motifs (CCUGGGG, CCUGGGA, CCUGGAA, and CCUGAGA) were reported [45]. We searched them in the 3′UTR of the isoforms that were detected only in the *UPF1* knockdown in A549 cells. As a result, 34 of the 91 isoforms included the p-UPF1 motifs. We also evaluated the motif enrichment in *UPF1*-depleted A549 cells using the MEME Suite [46] and found that possible GC-rich motifs were enriched in the 3′UTR of isoforms. Further studies would be necessary to confirm whether they can be actually bound by p-UPF1 (Additional file 12: Fig. S12A).

*SF3B1* is a well-known splicing factor that is mutated in several diseases and causes the increase of aberrant splicing isoforms [47, 48]. We assessed the effect of impairment of splicing by *SF3B1* knockdown to investigate whether aberrations of splicing factors influence isoform generation (Additional file 12: Fig. S10B). We found a significant increase in the proportion of exon skipping, which was concordant with the previous report using an SF3B1 inhibitor [47] (Fig. 3f). For example, exons 3 and 6 of *PSMD7* were altered in the knockdown condition (Fig. 3g). The expressions of exon-skipping isoforms of *PSMD7* were significantly increased, and RefSeq types were decreased conversely in *SF3B1*-depleted A549 cells (Fig. 3h, Additional file 12: Fig. S10E and S10F). To confirm consensus sequence in the proximal regions of the splice sites, we collected exon-skipping isoforms detected only in the *SF3B1* knockdown in A549 cells. For this analysis, we considered ± 10 bp regions surrounding regions of novel splicing junctions and skipped exons. Although the canonical splice consensus sequences of GT/AG were found in almost all the isoforms similar to the total RefSeq transcripts, no other distinct feature related to the *SF3B1* knockdown event was detected (Additional file 12: Figs. S12C and D). In addition, the genes with exon-skipping isoforms showed significant enrichment in the translation and ubiquitin-proteasome pathways (Additional file 12: Fig. S13). Similar enrichment of ubiquitin-proteasome pathways has been previously reported in a *SF3B1* knockdown experiment using myeloid cell lines [49]. Interruption of these factors, therefore, may alter at least some of the aberrant splicing isoforms and may contribute to their accumulation in lung cancer cells.

K700E is one of the most common hotspot mutations located in the HEAT-repeat domain of *SF3B1* [48, 50]. As shown in a previous study, the K700E hotspot mutation downregulates intron retention and upregulates alternative 3′ splice site events [51]. Except for exon skipping, the most affected splicing was intron retention (Additional file 12: Fig. S12B). This result was as expected, since it is considered to be a gain-of-function variant [50] and our knockdown assay of *SF3B1* should produce the opposite results. Alternative 3′ splice site events were not significantly affected, which was not always consistent with the previous results, suggesting other cellular contexts also play a role (Additional file 12: Fig. S12B).

### Aberrant transcripts as potential templates for producing neoantigens

**Fig. 4:**
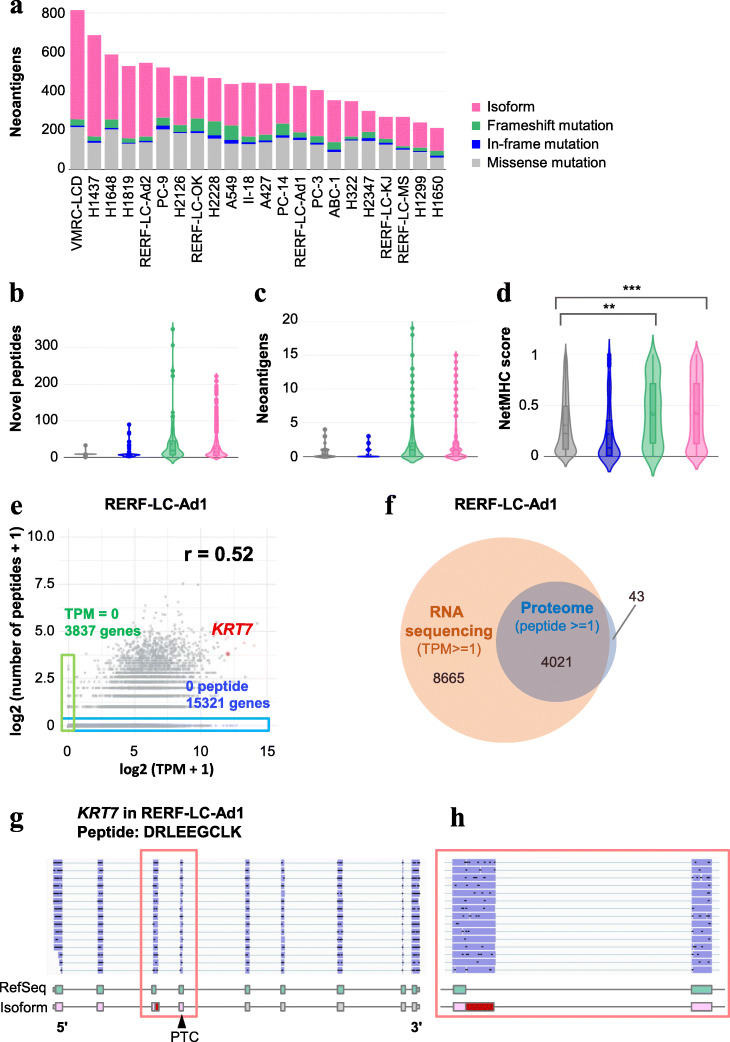
Neoantigens derived from each isoform or mutation type in cell lines. **a** The number of neoantigen candidates in each cell line. **b** The distribution of the number of novel peptides derived from tumor-specific regions of each isoform or mutation type. **c** The distribution of the number of neoantigen candidates from **b**. **d** The distribution of the maximum NetMHC score for each isoform or mutation type. ***P < 0.001 and **P < 0.01 (Kruskal–Wallis test and Dunn–Bonferroni’s post hoc test). **e** A correlation between the gene expression levels calculated by short-read RNA sequencing (TPM) and the number of peptides detected by proteome analysis for each gene in RERF-LC-Ad1 (*r* = 0.52). Both values + 1 were log2-transformed. Red points represent the *KRT7* gene with the isoform whose peptides were detected by proteome, the green area shows 0-TPM genes, and the blue area shows 0-peptide genes. **f** A comparison of genes detected by RNA sequencing (TPM ≥ 1) and by proteome (≥ 1 peptide) in RERF-LC-Ad1. Genes (4021/4064, 99%) detected by proteome were covered by RNA sequencing. **g**, **h** The full-length structure of the splicing isoform of *KRT7* (**g**) and the magnified inset of the alternative 5′ splice site region (**h**). The detected peptide of “DRLEEGCLK” was derived from exon 4 in the aberrant isoform

Accumulated aberrant splicing isoforms in tumors have been reported as possible sources of neoantigens [29–31]. To investigate whether the detected isoforms can be represented as neoantigens, we attempted to evaluate the potential antigenicity of the aberrant peptides encoded by these aberrant splicing isoforms. For this purpose, we estimated the binding affinity between peptides and HLA molecules using the standard method of NetMHC (Additional file 12: Fig. S14A). We conducted whole-genome sequencing (WGS) as well as sequence-based HLA typing for each cell line. Four-digit HLA types and the presence or absence of somatic mutations for each cell line was confidently determined (Additional file 12: Fig. S14B, Additional file 1: Table S1 and Additional file 4: Table S4). For the peptide side, based on the somatic mutations detected by the WGS, we deduced the altered peptide sequences of the isoforms by considering full-length transcript structures for all possible 9-mer peptides. Indeed, the aberrant splicing isoforms frequently and drastically altered the protein sequences by causing frameshift or early termination of translation (Additional file 12: Fig. S14C). Since these peptides were not represented in RefSeq or GENCODE databases, they may be peptides that have never been exposed to immune cells under normal conditions in healthy individuals. These neoantigens accounted for the greatest proportion of total potential neoantigens in most of the cell lines (Fig. 4a) Aberrant splicing isoforms and frameshift mutations contributed to the production of more of these novel peptides than missense or in-frame mutations (Fig. 4b). As expected, the number of neoantigens that were predicted as the “strong binder” by NetMHC was also greater both in splicing isoforms and frameshift mutations (Fig. 4c). In a comparison of the highest NetMHC scores of peptides from each isoform, the peptides that were derived from those aberrant isoforms showed higher score distribution than peptides of the missense and in-frame mutations that would be usually identified using the TMB detection approach (Fig. 4d).

To experimentally validate whether the aberrant isoforms were translated into proteins, we employed a shotgun proteomics approach using liquid chromatography coupled with tandem mass spectrometry (LC/MS/MS) for 11 NSCLC cell lines (A427, A549, H1650, H2228, II-18, PC-9, RERF-LC-Ad1, RERF-LC-Ad2, RERF-LC-KJ, RERF-LC-MS, and VMRC-LCD). For peptide identification using the Mascot search engine, we customized peptide sequence database based on MinION data for each cell line. As previously noted [52], short-read RNA sequencing showed a higher coverage of genes than LC/MS/MS proteomics due to its sequencing capacity (Fig. 4e and Additional file 12: Fig. S15). The number of peptides per gene detected by LC/MS/MS proteomics and TPM calculated by RNA sequencing data correlated (*r* = 0.52, Fig. 4e) and most of the genes detected by LC/MS/MS proteomics were also covered by RNA sequencing (Fig. 4f). We successfully detected 7 peptides translated from aberrant splicing isoform-specific regions (Table 1 and Additional file 12: Fig. S16). For example, the peptide derived from the isoform with alternative 5′ splicing in exon 3 of *KRT7* existed in RERF-LC-Ad1 (Fig. 4g). This isoform was not found in the GENCODE database, but the isoform-specific junction was observed in ENST00000547613 (KRT7-204 in Additional file 12: Fig. S17) which was considered as processed transcript without an open reading frame. The expression was also confirmed in H1437, H2126, and II-18 by MinION (Table 1). Furthermore, this isoform had the potential to produce several neoantigens predicted by NetMHC (Additional file 5: Table S5). These results suggest that at least some aberrant splicing isoforms were truly translated into peptides and could play a role in producing neoantigens in cancer.

**Table 1:** Peptide sequences derived from isoforms detected by proteome analysis

Detected peptide	Peptide-detected cell lines (proteome analysis)	Isoform-detected cell lines (MinION)	Gene symbol	Type	GENCODE
NLPSNPLEFNPDVLK	H1650, II-18, RERF-LC-KJ	A427, ABC-1, H1437, H1648, H1650, H1819, H2126, H2228, H2347, II-18, PC-9, RERF-LC-KJ, VMRC-LCD	ESYT2	Unannotated exon	ENST00000275418
NLPSNPLEFNPDVLKK	H1650, II-18, PC-9	A427, ABC-1, H1437, H1648, H1650, H1819, H2126, H2228, H2347, II-18, PC-9, RERF-LC-KJ, VMRC-LCD	ESYT2	Unannotated exon	ENST00000275418
TLGEIDAQHIQGVQETATDPR	H1650	H1650, H2228, H2347, II-18, RERF-LC-OK	FAM126A	Unannotated exon	ENST00000409923
DRLEEGCLK	RERF-LC-Ad1	H1437, H2126, II-18, RERF-LC-Ad1	KRT7	Alternative 5′ splice site	novel
EVPMVVVPPVGAK	A549, H2228	A549, H1437, H1819, H2228, H322	RRBP1	Alternative last exon	ENST00000398782
HLDAHTAAHSQSPR	RERF-LC-Ad2	H322, RERF-LC-Ad2	SUN1	Combination (shuffling, unannotated exon)	Novel
RHLDAHTAAHSQSPR	RERF-LC-Ad2	H322, RERF-LC-Ad2	SUN1	Combination (shuffling, unannotated exon)	Novel

### Aberrant splicing isoforms in lung cancer specimens

**Fig. 5:**
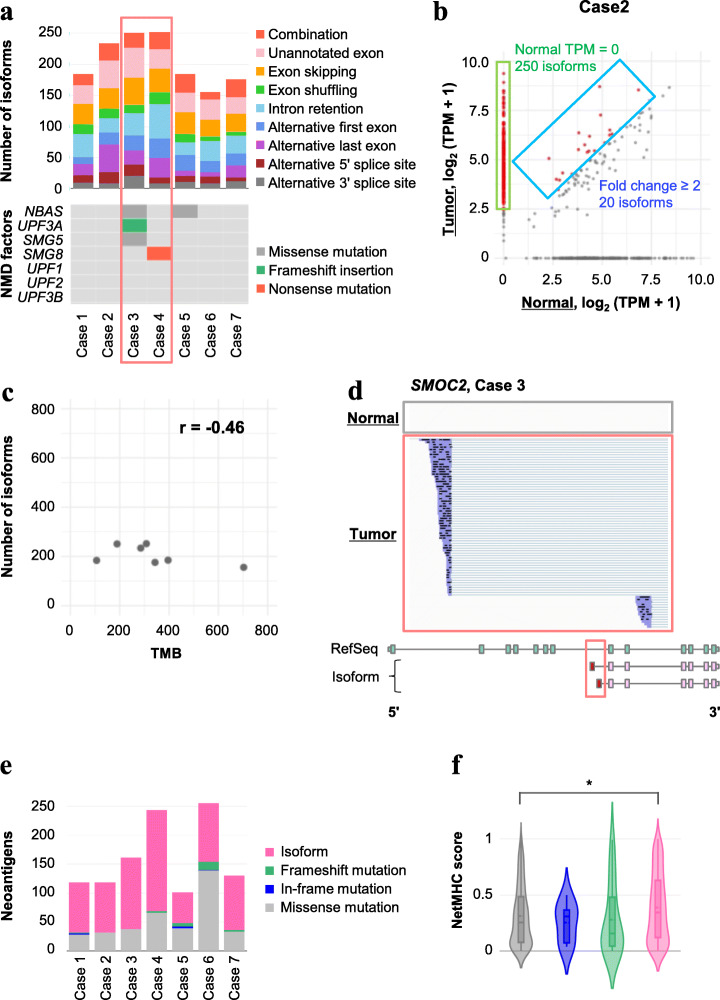
Tumor-specific isoforms and neoantigen candidates in clinical samples. **a** The number of splicing isoforms and the proportion of each splicing event (upper panel), and genes included in the NMD complex (lower panel) for each specimen. Cases 3 and 4 show more isoforms and harbor damaging mutations in NMD factors. **b** A comparison between the isoform expressions of non-tumor and tumor tissues in case 2. Red points represent tumor-specific isoforms (TPM in tumor tissue ≥ 10 and a fold change of TPM ≥ 2). The green area shows isoforms with 0 TPM in normal tissue and the blue area shows fold change of TPM ≥ 2 isoforms. **c** Correlation between the number of isoforms and the TMB for each specimen. No significant correlation was observed (*r* = − 0.46). **d** The full-length structure of splicing isoforms of SMOC2 in case 3. Unannotated exons were detected between exon 7 and exon 8. **e** The number of neoantigen candidates in each specimen. **f** The distribution of the maximum NetMHC score for each isoform or mutation type. *P < 0.05 (Kruskal–Wallis test and Dunn–Bonferroni’s post hoc test)

To examine whether aberrant splicing isoforms also exist in cancer cells in vivo, we next analyzed clinical lung cancer specimens (Additional file 6: Table S6). We applied the same analytical scheme used for the cell line analysis of clinical samples (Additional file 7: Table S7 and Additional file 8: S8). Using this method, we were again able to identify aberrant splicing isoforms in each patient (Fig. 5a). We selected the isoforms that were expressed at least twofold higher in the tumor samples than in the non-cancer counterparts (Fig. 5b) and identified 982 cancer-enriched splicing isoforms among all the clinical samples. Of these, 448 isoforms were not represented in either RefSeq or GENCODE (Additional file 12: Fig. S18A). There were no significant correlations between the number of aberrant isoforms and the TMB (Fig. 5c). As an example of the detected isoforms, novel alternative first exons in *SMOC2* are represented in Fig. 5d and were expressed only in the tumor of case 3. Similar to the results from the cell line analysis, we could identify several unique combination patterns of independent splicing events, and these patterns accounted for 14.5% of these unannotated isoforms (Additional file 12: Fig. S18B). Potential NMD-targeted isoforms that remained in the cancer cells were also identified, which suggests that the NMD mechanism is disrupted in the corresponding cancers (Additional file 12: Fig. S18C). Notably, in cases 3 and 4, the largest number of potential NMD-targeted isoforms were identified to harbor either frameshift or nonsense mutations in the key NMD factors, *UPF3B* and *SMG8* (Fig. 5a).

Similar to the method used for analysis of the cell line datasets, NetMHC analysis was conducted to identify peptides that could be potential neoantigens. For this, we identified somatic mutations and HLA types in each patient using the genomic sequencing data of the clinical samples (Additional file 12: Fig. S18D, Additional file 6: Table S6 and Additional file 9: Table S9). We detected 101–255 neoantigen candidates per case (Fig. 5e). We found that peptides derived from the splicing isoforms showed higher distribution scores compared to the peptides derived from missense mutations (Fig. 5f). Indeed, they accounted for the majority of total neoantigen candidate peptides in most samples (Fig. 5e). These results support the fact that aberrant splicing events in clinical samples can be detected by MinION and have great potential to produce more neoantigen candidate peptides than do missense mutations.

### Evaluation of the potential neoantigens derived from aberrant splicing isoforms in clinical samples

**Fig. 6:**
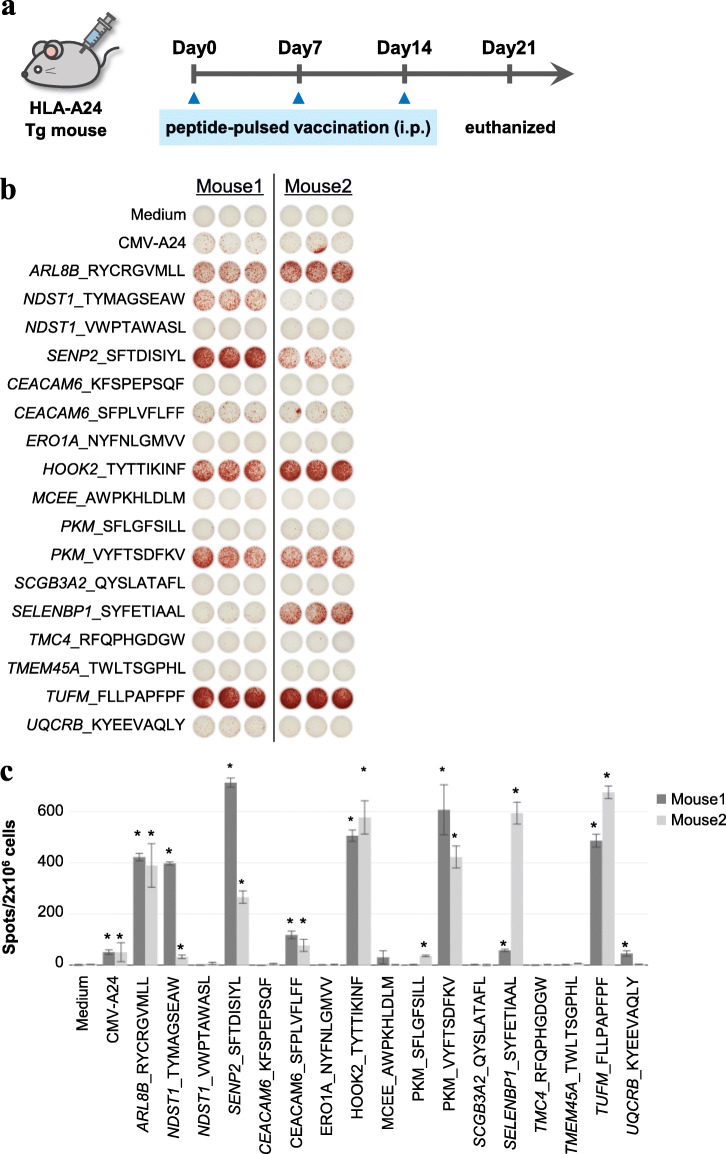
Biological validation of the antigenicity of neoantigen candidates in clinical samples. **a** Experimental scheme of immunization. HLA-A24 Tg mice were vaccinated with neoantigen candidate peptides three times (at days 0, 7, and 14). One week after the last immunization, mice were euthanized and isolated splenocytes were used for IFN-γ ELISpot assay. **b** The results of the ELISpot assay. **c** Statistical analysis of the results of ELISpot. Dark gray and light gray bars indicate independent mice experiments. *P < 0.05 (Student’s *t* test) and signal-to-noise ratio > 5. Error bars represent the SEM

To evaluate the antigenicity of peptides that were identified from the aberrant splicing isoforms and frameshift mutations, we immunized HLA-A24 transgenic mice with the candidate peptides according to the scheme shown in Fig. 6a. We chose 17 candidate peptides based on the NetMHC scores for HLA-A:24:02 (Table 2 and Additional file 12: Fig. S19). We confirmed that the peptide sequences showed no similarity with those of human or mouse protein databases by BLAST-P, but non-cancer-specific regions, which were similar to those of RefSeq transcripts, were represented in both human and mouse. One week after the last vaccination, we isolated splenocytes from mice and subjected the isolates to an enzyme-linked immune absorbent spot (ELISpot) assay. By doing so, we attempted to detect neoantigen-specific spleen lymphocyte responses. The ELISpot results showed that 8 out of 17 peptides induced significantly high IFN-γ production (*n* = 2) compared to the PBS and adjuvant-alone groups (Fig. 6b, c). These results demonstrate that peptides derived from splicing isoforms and frameshift mutations could activate the T cell response through interaction with HLAs.

**Table 2:** Peptide sequences tested using ELISpot assay

Peptide sequence	gene	NetMHC score	Isoform-detected samples	Isoform/frameshift	GENCODE	isoform type
TYMAGSEAW	NDST1	0.0327	Case 6	Frameshift	Frameshift	Frameshift
VWPTAWASL	NDST1	0.1467	Case 6	Frameshift	Frameshift	Frameshift
RYCRGVMLL	ARL8B	0.2162	Case 4	Frameshift	Frameshift	Frameshift
SFTDISIYL	SENP2	0.3557	Case 5	Frameshift	Frameshift	Frameshift
KFSPEPSQF	CEACAM6	0.0565	Case 4	Isoform	Novel	Alternative last exon
SFPLVFLFF	CEACAM6	0.0857	Case 4	Isoform	Novel	Alternative last exon
NYFNLGMVV	ERO1A	0.4236	Case 2	Isoform	Novel	Alternative last exon
TYTTIKINF	HOOK2	0.0185	ABC-1, H1437, H1648, H1650, H2126, H322, II-18, PC-3, PC-9, RERF-LC-Ad1, RERF-LC-Ad2, RERF-LC-KJ, RERF-LC-OK, Case 6, Case 7, VMRC-LCD	Isoform	ENST00000589134	Alternative last exon
AWPKHLDLM	MCEE	0.3848	ABC-1, H2126, H322, II-18, PC-3, RERF-LC-KJ, Case 1, Case 7	Isoform	Novel	Alternative 5′ splice site
VYFTSDFKV	PKM	0.2639	ABC-1, H1299, H1437, H1648, H2126, H2347, H322, II-18, RERF-LC-Ad1, RERF-LC-Ad2, Case 4, VMRC-LCD	Isoform	Novel	Alternative last exon
SFLGFSILL	PKM	0.3814	ABC-1, H1299, H1437, H1648, H2126, H2347, H322, II-18, RERF-LC-Ad1, RERF-LC-Ad2, Case 4, VMRC-LCD	Isoform	Novel	Alternative last exon
QYSLATAFL	SCGB3A2	0.2403	Case 6	Isoform	Novel	Unannotated exon
SYFETIAAL	SELENBP1	0.0848	Case1, Case 4, Case 6	Isoform	ENST00000493560	Intron retention
RFQPHGDGW	TMC4	0.2835	H322, RERF-LC-Ad2, Case 1	Isoform	Novel	Alternative last exon
TWLTSGPHL	TMEM45A	0.2974	Case4, Case7	Isoform	Novel	Combination (unannotated exons)
FLLPAPFPF	TUFM	0.3991	A427, ABC-1, H1299, RERF-LC-Ad1, RERF-LC-Ad2, Case 1, VMRC-LCD	Isoform	Novel	Intron retention
KYEEVAQLY	UQCRB	0.447	H1819, Case 2, Case 3	Isoform	ENST00000519322	Alternative last exon

### Re-evaluation of the TCGA short-read sequencing data from the viewpoint of potential neoantigen candidates

**Fig. 7:**
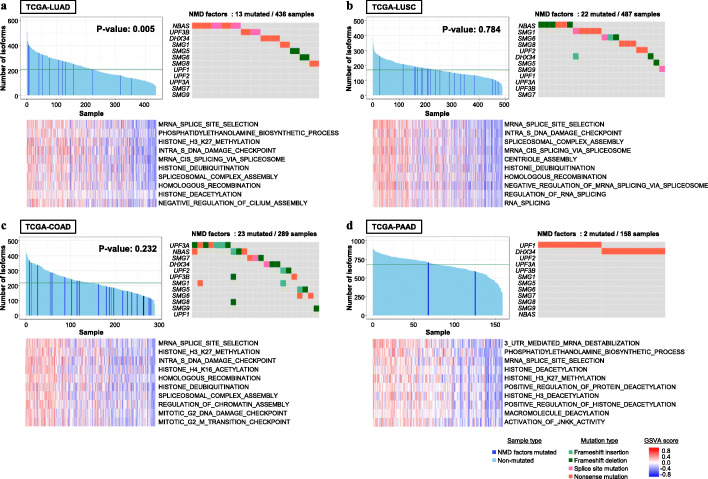
Clinical relevance of our aberrant isoform catalogs in TCGA. **a**–**d** The number of isoforms calculated from the TCGA short-read RNA sequencing dataset (left panel), somatic mutation patterns of genes included in the NMD factors (right panel), and heatmaps showing the GSVA enrichment scores of the gene sets of the gene ontology biological process in lung adenocarcinoma (**a**), lung squamous cell carcinoma (**b**), colon adenocarcinoma (**c**), and pancreatic adenocarcinoma (**d**). Blue bars in the left panels represent specimens with damaging mutations in the NMD factors and green lines represent the median of isoforms. The *P* value was calculated using the one-tailed Mann–Whitney *U* test for testing the positive association of NMD factor mutations with a higher number of isoforms

Finally, to further utilize our aberrant isoform catalog based on MinION sequencing data, we re-evaluated the whole-exome sequencing and short-read RNA sequencing datasets of 436 lung adenocarcinoma (LUAD) and 46 matched normal samples registered in The Cancer Genome Atlas (TCGA). We mapped their RNA sequencing data to our isoform catalog. We then counted the reads which were aligned to the isoform-specific regions according to our catalog (Additional file 12: Fig. S20A, see detail in “Materials and methods”). To eliminate the isoforms in non-tumor lung tissues, we also constructed an isoform panel of normal samples. We selected isoforms that expressed at least two-fold higher in tumor samples compared with those in the panel of normal. In addition, we removed isoforms whose junctions were expressed in the lung tissues samples stored in the Genotype-Tissue Expression (GTEx) project database [53] and counted the number of isoforms for each specimen (see “Materials and methods” for additional details on the procedure). Among the novel splice junctions, which were not represented in either RefSeq or GENCODE, only 12.5% were represented in the lung specimens of GTEx, and 52% were novel junctions even in all tissues taken together in GTEx (Additional file 12: Fig. S20B). As a result, we found 13 specimens harboring damaging mutations on the NMD factors of frameshift, nonsense, and splice site mutations (Fig. 7a, upper right panel), and these cases showed significantly higher distributions of the number of isoform distributions compared to the others (Fig. 7a, upper left panel). We further expanded this analytical scheme to other cancer types, such as lung squamous cell carcinoma (LUSC), colon adenocarcinoma (COAD), and pancreatic adenocarcinoma (PAAD). We determined that some of the isoforms were commonly expressed (Fig. 7b–d). The number of isoforms did not correlate with the TMB or cancer stage in any types of cancer (Additional file 12: Figs. S20C and D). We also found that the damaging profile of NMD factors was not always consistent with the number of isoforms, depending on cancer subtypes (Fig. 7b–d). NMD efficiency differed depending on the PTC position in isoforms [54] and tissues [55]. Moreover, the impact of NMD in individual cancer types has been partly indicated by recent studies [56, 57]. By expanding our analysis scheme to a pan-cancer approach, these aberrant splicing isoforms would facilitate a new field to search neoantigen targets and provide insights into NMD in a variety of cancer types.

For more detailed characterizations of each sample, we applied a gene set variation analysis (GSVA) [58] and calculated the correlation between the number of isoforms and the GSVA enrichment scores. Notably, we found positive correlations between the number of isoforms and the splicing-related signature scores in all cancer types (Fig. 7a–d, lower panel). These results suggest that the upregulation of splicing-related genes may contribute to the increase in the number of aberrant isoforms. Otherwise, there was no significant relationship between the number of isoforms and the specific immune cell signatures for each cancer type (Additional file 12: Figs. S20E) [59, 60]. Since the clinical specimens we analyzed did not have any immune status information, such as the proportion of tumor-infiltrating lymphocytes or the ICI response, further study is necessary to conclude the effects of these aberrant isoforms on immune system response to tumors.

## Discussion

In this study, we examined the full-length transcript structures of aberrant splicing isoforms by using long-read sequencing data. We constructed a catalog of such isoforms for the NSCLCs. To address the inaccurate sequencing of MinION, we used short-read sequencing data to identify the exact splicing junctions. This approach revealed a substantial number of novel aberrant transcripts and some of these isoforms were detected as peptides by the proteome analysis. Furthermore, an ELISpot assay demonstrated that at least some of these isoforms, having strong antigenicity, had the potential of becoming neoantigens. These results clearly show the capability of long-read sequencing to search for novel isoforms and neoantigens that may be overlooked by the current short-read sequencing approaches.

In the clinical setting, the TMB, which is determined by the total number of nonsynonymous mutations, is considered to be a predictor of the effectiveness of immunotherapy for cancer. However, the prediction accuracy is not sufficient for many types of cancer. The current study shows that aberrant splicing isoform and frameshift mutations have a significant potential for producing a larger number of neoantigens. Multiple previous reports suggest that this potential may have an influence on shaping the tumor immune landscape of the patients in addition to the genomic TMB [28–30]. When transcriptomes cannot be directly examined in a given clinical setting, the NMD status and other splicing factors may provide important information. Indeed, previous reports have indicated that the number of frameshift mutations that are predicted not to trigger NMD is higher in responders to immunotherapy and that factor combined with the TMB improves the accuracy of prediction for immunotherapy responders [61].

We mainly focused on NSCLC in this study. However, we were able to identify potential aberrant isoforms for other types of cancer by considering our full-length catalog. Obviously, when as more cancer types are added to the catalog, the sensitivity and selectivity of the detection will further increase. Also note that, since GTEx is based on short-read RNA sequencing datasets, the representation of the junctions in GTEx do not necessarily indicate the existence of the isoform in their full-length forms in normal tissues. To calculate and extract truly cancer-specific isoform patterns, we should generate a larger dataset of long-read isoforms for normal tissues in future study. Further in-depth analyses would elucidate the possibility that these transcriptomic features provide a complementary indicator for predicting the effectiveness of immunotherapy, in addition to genomic features.

## Conclusions

n this study, we indicated that the long-read sequencing of full-length cDNAs in tumors is essential to precisely identify aberrant transcript structures that have been overlooked by short-read sequencing. The aberrant splicing isoforms showed a significant potential for producing a larger number of neoantigens in tumors. These novel transcriptomic features obtained from the long-read sequencing would be helpful for estimating the tumor immune landscapes, which could potentially improve the accuracy of prediction of responders to immunotherapy when used in combination with the current indicators that only use genomic mutations.

## Material and methods

### Cell lines

Twenty two lung adenocarcinoma cell lines (A427, A549, ABC-1, H1299, H1437, H1648, H1650, H1819, H2126, H2228, H2347, H322, II-18, PC-14, PC-3, PC-9, RERF-LC-Ad1, RERF-LC-Ad2, RERF-LC-MS, RERF-LC-OK, RERF-LC-KJ, and VMRC-LCD, Additional file 1: Table S1) were cultured as previously described [1]. Raw sequence data were obtained from the DNA Data Bank of Japan (DDBJ) with accession numbers DRA001859 (whole-genome sequencing), DRA001846 (short-read RNA sequencing) [1], and DRA008295 (long-read RNA sequencing of RERF-LC-KJ, RERF-LC-MS, and PC-9, 62].

### Clinical samples

Clinical samples were obtained with the appropriate informed consent from the National Cancer Center Japan. Surgical specimens from seven patients were pathologically checked. All seven patients were previously diagnosed with primary lung cancer, including four cases of lung squamous cell carcinomas and three cases of lung adenocarcinomas. Fresh frozen surgical specimens were used to extract DNA and RNA as described below.

### Full-length RNA sequencing using MinION

Total RNA was isolated and purified using an RNeasy Mini Kit (Qiagen). Library preparation for full-length transcriptome analysis using MinION (Oxford Nanopore Technologies) was performed as previously described [32]. In brief, 1.5 μg of the full-length-cDNA library was applied for 1D^2^ sequencing according to the manufacturer’s protocol with some modifications using the 1D^2^ sequencing kit, SQK-LSK308, and R9.5 flow cell, FLO-MIN107 (Oxford Nanopore Technologies).

### Conventional short-read RNA sequencing

For A549 used for knockdown assays, total RNA was isolated and purified using an RNeasy Mini Kit (Qiagen). Libraries were prepared using a TruSeq Stranded mRNA kit. Then RNA sequencing was conducted using the NovaSeq 6000 platform (Illumina) with 150 bp paired-end reads according to the manufacturer’s protocol.

For clinical samples, total RNA was extracted by an RNeasy Mini Kit and re-purified with an RNeasy MinElute Cleanup Kit (Qiagen) for some cases. Library preparation was performed using a TruSeq Stranded mRNA kit. Sequencing was performed using the HiSeq 2500 platform (Illumina) with 75 bp paired-end reads.

### Conventional short-read DNA sequencing

Genomic DNA was extracted using a DNeasy Blood & Tissue kit (Qiagen). In total, 150 ng aliquots of genomic DNA from paired cancerous and non-cancerous samples were fragmented by a Covaris-E220 evolution instrument (Covaris) to provide DNA fragments with a base pair peak at 150 to 200 bp. The DNA fragments were end-repaired and ligated with paired-end adaptors (SureSelect XT Library Prep Kit, Agilent Technologies). The resulting DNA library was purified using an Agencourt AMPure XP Reagent (Beckman Coulter) and amplified by PCR (9 cycles). In total, 750 ng aliquots of the adaptor-ligated libraries were hybridized for 16–24 h at 65 °C with biotinylated oligo RNA bait, SureSelect Human All Exon V5 (Agilent Technologies). The hybridized genomic DNA was subjected to ten cycles of PCR re-amplification. Following the manufacturer’s standard protocols, the whole-exome DNA library was sequenced on the Illumina HiSeq 2500 platform (Illumina) with 75 bp paired-end reads.

### Analysis of long-read sequencing data

Base calling of the FAST5 data from MinION was performed using Albacore 2.2.7 and Guppy v3.3.0, then was converted into FASTQ files. Only 1D reads for the following analyses [62]. Reads were aligned to the reference human genome, GRCh38, using Minimap2 (v2.2.14) [33]. To eliminate pseudogene mapping and low-quality reads, aligned reads that met any of the following five conditions were discarded: (1) secondary or supplementary aligned flag; (2) mapping identity, defined as the percentage of matched bases to the sum of matched bases, substitutions, insertions, and deletions, lower than 0.8; (3) unmapped length of reads within splice junctions longer than 10 bp; (4) exon length shorter than 25 bp; (5) overlapping the pseudogene region of GENCODE v27. After these filtering steps, introns shorter than 50 bp were corrected as exons and extracted reads were assigned to a single gene for further analysis. Seqtk (https://github.com/lh3/seqtk) was used for subsampling reads in the fastq file.

### Analysis of short-read RNA sequencing

Raw reads were trimmed with quality < 20 and adaptor filtered using Trimmomatic (v0.32) [63]. rRNA sequences were removed by Bowtie2 (v 2.3.5.1) [64]. Cleaned up reads were aligned to the reference human genome, GRCh38, and transcripts per million (TPM) was calculated using STAR (v2.6.1d) [65] and RSEM (v1.3.1) [66].

### Analysis of short-read DNA sequencing

Raw reads with a quality < 20 were trimmed and adaptor filtered using Trimmomatic (v0.32) then were aligned to reference human genome GRCh38 by BWA-mem (v0.7.17) [67]. We preprocessed the BAM files including marking duplicates and base recalibration steps using GATK (v4.0.10.1) [68]. For variant calling, we used HaplotypeCaller (GATK) for lung cancer cell line samples and filtered low confidence SNPs using the recommended hard filter settings: QD < 2.0, FS > 60.0, MQ < 40.0, MQRankSum − 12.5, ReadPosRankSum < − 8.0. The following filters were also applied to remove low confidence indels: QD < 2.0, FS > 200.0, ReadPosRankSum < − 20.0. Mutect2 and FilterMutectCalls were used for clinical samples with default parameters and tumor-normal mode. We filtered out variants with allele depth < 5 and annotated them with Variant Effect Predictor (VEP, v95) [69]. To extract somatic variants from cell line sample data, we eliminated variants that were registered in dbSNP (v151) unless they were also present at least 5 samples in COSMIC (v90) from the dataset.

### Computational analysis for detection of transcript isoforms from MinION data

We compared all junctions in MinION reads with the RefSeq transcripts (downloaded from the UCSC Genome Browser and Blat software (University of California Santa Cruz) in July 2017, allowing for a margin of 20 bp gaps. Then, we removed reads that were identical to RefSeq model transcripts (Type A) or were truncations of RefSeq model transcripts (Type B).

Type A satisfied three conditions: (1) It ignored the difference between the five-prime and the three-prime end; (2) it had the same number of exons; (3) the junctions from the read were the same as those of the RefSeq model transcripts.

Type B included the remaining reads that satisfied the following three conditions: (1) The putative truncated end of the read was located within the exon; (2) the number of exons was different; (3) the junctions from the read were the same as those of the RefSeq model transcripts.

Considering the inaccuracy of MinION reads, we compared the remaining reads to the short-read sequencing junction sets detected in at least five reads using STAR. If a junction in the read was not identical to that of an RefSeq transcript and was confirmed with short-read sequencing, we classified the read as an isoform. We compared all junctions in the MinION reads with those of the RefSeq transcripts.

After merging isoforms that contained the same junctions, the isoforms were filtered using the following condition: MinION read coverage ≥ total MinION reads/100,000. Then, we create a GTF file of the isoforms and RefSeq transcripts for each sample. Using the GTF file as a reference, we mapped the reads from the short-read sequencing and calculated the TPM. To remove low confidence isoforms, the following filters were applied: TPM ≥ 10, isoform read counts/total read counts assigned to the same gene > 10%. Finally, we classified isoforms by the following nine types: alternative 5′ splice site, alternative 3′ splice site, alternative first exon, alternative last exon, intron retention, exon shuffling, intron retention, unannotated exon, and the corresponding combination patterns (Fig. 1a).

### Detection of transcript isoforms using TALON

After aligning the reads, TranscriptClean (v2.0.2) [36] was performed for error correction of the SAM file using the junction file that was obtained from the short-read RNA sequencing by STAR. TALON (v5.0.0) [35] was executed using the parameter setting --cov 0.5. We counted isoform patterns that harbored the same splice junctions ignoring strand.

### siRNA knockdown in A549

Reverse siRNA transfections were performed using Lipofectamine RNAiMAX (Thermo Fisher Scientific) and siRNA sets (Additional file 10: Table S10). siRNA for *UPF1* was kindly provided by Dr. R. Onoguchi-Mizutani and Dr. N. Akimitsu from The University of Tokyo [45]. We applied 75 pmol of siRNA for *UPF1* knockdown and mixed two different siRNAs (12.5 pmol each) for *SF3B1*. Lipofectamine RNAiMAX (7.5 μl) and each siRNA were diluted in 400 μl of OptiMEM, then incubated for 20 min at room temperature. We seeded 1.2 × 10^5^ cells of A549 in antibiotic-free DMEM into each well of a 6-well plate. After incubation, we added 400 μl of the RNAiMAX and siRNA mixture to each well. After 24 h, we replenished the medium and re-added the RNAiMAX and siRNA mixture. Cells were collected 48 h after the last transfection.

### RT-PCR assay and bioanalyzer

Total RNA was isolated using an RNeasy Mini Kit (Qiagen), and cDNA synthesis was performed using a SMART-Seq v4 Ultra Low Input RNA Kit for Sequencing (Takara Bio) as previously described [32]. RT-PCR was performed using Power SYBR Green Master Mix (Thermo Fisher Scientific) and the LightCycler 96 system (Roche). The data were normalized to the expression of GAPDH or common exonic regions of each gene using the 2−ΔΔCt method. We quantified and compared each PCR product size using a bioanalyzer (Agilent Technologies). Primers were designed using Primer3 Plus [70] and are listed in Additional file 11: Table S11. The RT-PCR experiments were conducted in triplicate.

### Identification of repetitive elements in splice sites

RepeatMasker (v4.1.0) [71] was performed to detect repetitive elements with the “-species human” option. We extracted ±50 bp regions around the splice sites and counted sequences with > 80% to repetitive elements.

### Motif enrichment analysis

To find splice site consensus motifs, we collected exon-skipping isoforms in *SF3B1*- depleted A549 cells. We extracted ± 50 bp regions around the splice sites or skipping exons and generated sequence logos using WebLogo (v2.8.2) [72]. Motif enrichment analysis was performed by using MEME Suite (v5.1.1) [46] with the maximum width of motifs set to ten. The sequences of 3′UTR in aberrant splicing isoforms in *UPF1*-depleted A549 cells were extracted as the input for the analysis. 3′UTR sequences of all RefSeq transcripts were used to generate the background model based on the hidden Markov model.

### Computational analysis for the detection of antigenic peptides

All possible 9-mer peptides were computed considering the variants and isoform patterns. We filtered out peptides listed in GENCODE v31 or RefSeq to extract cancer-specific aberrant peptides. HLA class I loci (HLA-A, B, and C) were typed at four-digit resolution using OptiType (v1.3.1) [73]. Binding affinities between peptides and HLA alleles were predicted using NetMHCpan4.0 [74], and strong binders were defined as having %rank < 0.5.

### Materials for LC/MS/MS proteomics

Ammonium bicarbonate, sodium deoxycholate (SDC), sodium N-lauroylsarcosinate (SLS), tris (hydroxymethyl) aminomethane (Tris), dithiothreitol (DTT), iodoacetamide (IAA), lysyl endopeptidase (Lys-C), ethyl acetate, acetonitrile (ACN), acetic acid, and trifluoroacetic acid (TFA) were obtained from FUJIFILM Wako. Protease inhibitor cocktail and phosphatase inhibitor cocktail 2 and 3 were obtained from Sigma-Aldrich. Trypsin was obtained from Promega. Empore disks for StageTips were obtained from GL Sciences.

### Sample preparation for LC/MS/MS proteomics

Protein digestion was performed according to the phase transfer surfactant (PTS) protocol [75]. Briefly, cell lysates were lysed in a PTS buffer (12 mM SDC, 12 mM SLS, 1% protease inhibitor, and 1% phosphatase inhibitors 2 and 3 in 100 mM Tris-HCl, pH 9.0). Proteins were reduced with 10 mM DTT and alkylated with 50 mM IAA. After five-fold dilution with 50 mM ammonium bicarbonate, Lys-C and trypsin were added at a 1:100 (w/w) protease-to-protein ratio, followed by incubation overnight at 37 °C. Then an equal volume of ethyl acetate was added, and the solution was acidified with TFA. After removing the organic phase, the samples were dried by SpeedVac and reconstituted in 5% ACN and 0.1% TFA. The peptides were fractionated into eight fractions with SCX-StageTips and desalted using SDB-XC StageTips [76, 77].

### LC/MS/MS analyses

The peptides were analyzed by Orbitrap Fusion Lumos mass spectrometry (Thermo Fisher Scientific) coupled with an UltiMate 3000 RSLCnano (Thermo Fisher Scientific) pump and an HTC-PAL autosampler (CTC Analytics). Self-pulled needle columns (150 mm length, 100 μm ID, 6 μm needle opening) packed with ReproSil-Pur C18-AQ (3 μm, Dr. Maisch) were used as analytical columns. The injection volume was 5 μl and the flow rate was 500 nl/min. The mobile phases consisted of 0.5% acetic acid (A) and 0.5% acetic acid in 80% ACN (B). The gradient program was as follows: 5–40% B (65 min), 40–99% B (5 min), 99% B (10 min), 99–5% B (0.1 min), and 5% B (29.9 min). Both MS1 and MS2 scans were obtained with Orbitrap. The MS1 survey scan was performed at 120,000 resolution in the scan range of m/z 300–1500 with an AGC target value of 4e5. The MS2 scan was performed at a resolution of 15,000 with an AGC target value of 5e4, and the scan cycle was 3 s. Dynamic exclusion was set to 30 s, and the normalized collision energy for HCD was 38%.

### Identification and quantification of peptides and proteins

Peak lists were generated from the raw MS/MS spectra using MaxQuant ver.1.6.2.10 [78]. Then the resulting mgf files were searched against our peptide sequence database based on MinION data using the Mascot search engine (Matrix Science) with a precursor mass tolerance of 5 ppm and a fragment ion mass tolerance of 20 ppm. Carbamidomethylation on cysteine was set as fixed modifications. Oxidation on methionine was set as a variable modification. Up to one missed cleavage was allowed for trypsin and Lys-C digestion. The results were filtered at a 1% peptide-level false discovery rate (FDR).

### Mice

HLA-A24 transgenic (A24Tg) mice were kindly provided by Institute Pasteur [79] (Paris, France), and bred in Sankyo-Lab Service. Young adult (7- to 20-week-old) mice were maintained under specific pathogen-free conditions in our animal facility and were used for all experiments.

### Peptide vaccination and ELISpot assays

A24Tg mice were immunized subcutaneously three times (at days 0, 7, and 14) with 50 μg peptides pooled from 5 predicted peptides, and with 8 μg poly-ICLC (Hiltonol, ONCOVIR) used as the adjuvant. One week after the last vaccination, the A24Tg mice were euthanized and splenocytes were harvested. To detect peptide-specific immune response, IFN-γ ELISpot assays were performed using a BD ELISPOT kit for Mouse IFN-γ (BD Bioscience) according to the manufacturer’s protocols. 2 × 10^6^ splenocytes were incubated with 10 μg of each peptide for 20 h at 37 °C and 5% CO_2_. A peptide (QYDPVAALF) derived from cytomegalovirus pp65 was used as a positive control. All analyses were performed in triplicate for two mice. The spots were automatically counted by the Eliphoto system (Minerva Tech). The immune response was considered to be positive when the *P* value for replicates was < 0.05 and the ratio of spot counts for wells vs. the negative controls was > 5.

### Computational analysis of GTEx data

The exon-exon junction read count matrix was downloaded from the GTEx Portal (https://gtexportal.org/home/) [53]. We extracted junction sets detected from at least 20 reads for each sample, then collected junctions that were expressed as “normal junctions” in each tissue in more than 50% of specimens.

### Computational analysis of TCGA data

BAM files for short-read RNA sequencing mapped by STAR and VCF files for somatic mutations detected by the MuTect2 algorithm in the TCGA were downloaded from the National Cancer Institute Genomic Data Commons data portal (https://portal.gdc.cancer.gov/) [80].

We built genome index files for STAR using the reference human genome and our constructed isoform catalog. BAM files were converted into FASTQ files and remapped using the index. We extracted isoforms covered by at least 20 reads that were mapped to isoform-specific exons or junctions. The panel of normal samples was constructed using the max RPM of each isoform expressed in at least five matched normal samples. Finally, we identified tumor-specific isoforms that expressed at least twofold higher than in the panel of normal samples and not represented in normal junction datasets of GTEx.

VCF files were filtered using the filtering conditions of TCGA, annotated using VEP, and converted into MAF files by vcf2maf (https://github.com/mskcc/vcf2maf) for visualization with Maftools [81].

### Statistics

Statistical analysis was performed using Python3.6.5 and R3.5.0 software. For a nonparametric multiple comparison of the distributions, we used the nonparametric Kruskal–Wallis test followed by Dunn’s post hoc test adjusted with Bonferroni correction. Fisher’s exact tests were performed for gene ontology enrichment analysis and to compare the proportion of isoforms in knockdown experiments. Welch’s *t*-test was used to compare the number of isoforms with driver mutation. Student’s *t* test was used for ELISpot assay and the two-tailed Mann–Whitney *U* test was used for the TCGA analysis.

### Gene ontology analysis

The Cytoscape Bingo plugin was used with gene ontology annotations [82]. To remove redundancies, we took account of gene ontology terms with > 50 and < 1000 genes. The *P* value was adjusted by Benjamini and Hochberg FDR correction. The result of the enrichment analysis was visualized using a REVIGO treemap [83].

### Gene set variation analysis

For single-sample gene set enrichment, we used the gene set variation analysis [58] (GSVA) program to derive the absolute enrichment scores for gene signatures using the following datasets: (1) the C5 gene ontology biological process subset of the Molecular Signature Database version 7.2 [84], and (2) gene sets representing immune cell populations reported in several previous publications [59, 60]. We applied log_2_TPM values calculated using RSEM for GSVA with the options “min.size = 10” and “max.size = 1000.” Spearman’s rank correlation coefficient was calculated for the number of isoforms and GSVA scores.

## Supplementary Information

**Additional file 1: Table S1.** General information on NSCLC cell lines.

**Additional file 2: Table S2.** General statistics of nanopore sequencing in NSCLC cell lines.

**Additional file 3: Table S3.** The number of aberrant splice isoforms in NSCLC cell lines.

**Additional file 4: Table S4.** The number of somatic mutations in NSCLC cell lines.

**Additional file 5: Table S5.** The neoantigen candidates derived from isoforms detected by proteome analysis.

**Additional file 6: Table S6.** Clinical information on clinical samples.

**Additional file 7: Table S7.** General statistics of nanopore sequencing in clinical samples.

**Additional file 8: Table S8.** The number of aberrant splice isoforms in clinical samples.

**Additional file 9: Table S9.** The number of somatic mutations in clinical samples.

**Additional file 10: Table S10.** siRNA used for knockdown experiments in A549.

**Additional file 11: Table S11.** Primers used for RT-PCR analysis.

**Additional file 12.** Supplementary figures.

**Additional file 13.** Aberrant splicing isoform dataset of cell lines (gtf). The full-length structures of isoforms detected in our dataset of cell lines.

**Additional file 14.** Aberrant splicing isoform dataset of clinical specimens (gtf). The full-length structures of isoforms detected in our dataset of clinical specimens.

**Additional file 15.** Review history.

## Data Availability

The sequencing data of cell lines have been published in the DNA Data Bank of Japan (DDBJ) [85] under accession numbers DRA010214 [86] and DRA010215 [87]. The sequencing data for clinical samples have been deposited to the Japanese Genotype-phenotype Archive (JGA) [88], which is hosted by the National Bioscience Database Center (NBDC) and DDBJ, under accession number JGAS00000000245 [89]. The proteomics data have been deposited to the ProteomeXchange Consortium [90] via the jPOST partner repository [91, 92] with the dataset identifier PXD019915 [93].

## References

[1] Suzuki A, Makinoshima H, Wakaguri H, Esumi H, Sugano S, Kohno T (2014). Aberrant transcriptional regulations in cancers: genome, transcriptome and epigenome analysis of lung adenocarcinoma cell lines. Nucleic Acids Res.

[2] Nicholson P, Yepiskoposyan H, Metze S, Orozco RZ, Nicole Kleinschmidt OM (2010). Nonsense-mediated mRNA decay in human cells. Cell Mol Life Sci.

[3] Lu JW, Plank TD, Su F, Shi XJ, Liu C, Ji Y (2016). The nonsense-mediated RNA decay pathway is disrupted in inflammatory myofibroblastic tumors. J Clin Invest.

[4] Liu C, Karam R, Zhou Y, Su F, Ji Y, Li G (2014). The UPF1 RNA surveillance gene is commonly mutated in pancreatic adenosquamous carcinoma. Nat Med.

[5] Popp, Maximilian W. LEM. Nonsense-mediated mRNA decay and cancer. Curr Opin Genet Dev. 2018;48:44–50. 10.1016/j.gde.2017.10.007 PMC5869107 29121514

[6] Cowen LE, Tang Y (2017). Identification of nonsense-mediated mRNA decay pathway as a critical regulator of p53 isoform β. Sci Rep.

[7] Karam R, Carvalho J, Bruno I, Graziadio C, Senz J, Huntsman D (2008). The NMD mRNA surveillance pathway downregulates aberrant E-cadherin transcripts in gastric cancer cells and in CDH1 mutation carriers. Oncogene..

[8] Pastor F, Kolonias D, Giangrande PH, Gilboa E (2010). Induction of tumour immunity by targeted inhibition of nonsense-mediated mRNA decay. Nature..

[9] Bokhari A, Jonchere V, Lagrange A, Bertrand R, Svrcek M, Marisa L (2018). Targeting nonsense-mediated mRNA decay in colorectal cancers with microsatellite instability. Oncogenesis..

[10] Seiler M, Peng S, Agrawal AA, Palacino J, Teng T, Zhu P (2018). Somatic mutational landscape of splicing factor genes and their functional consequences across 33 cancer types. Cell Rep..

[11] Graubert TA, Shen D, Ding L, Okeyo-Owuor T, Lunn CL, Shao J (2012). Recurrent mutations in the U2AF1 splicing factor in myelodysplastic syndromes. Nat Genet.

[12] Yoshida K, Sanada M, Shiraishi Y, Nowak D, Nagata Y, Yamamoto R (2011). Frequent pathway mutations of splicing machinery in myelodysplasia. Nature..

[13] The Cancer Genome Atlas Research Network (2014). Comprehensive molecular profiling of lung adenocarcinoma. Nature..

[14] Furney SJ, Pedersen M, Gentien D, Dumont AG, Rapinat A, Desjardins L (2013). SF3B1 mutations are associated with alternative splicing in uveal melanoma. Cancer Discov.

[15] Hsu TYT, Simon LM, Neill NJ, Marcotte R, Sayad A, Bland CS (2015). The spliceosome is a therapeutic vulnerability in MYC-driven cancer. Nature..

[16] El Marabti E, Younis I (2018). The cancer spliceome: reprograming of alternative splicing in cancer. Front Mol Biosci.

[17] Lee SCW, Abdel-Wahab O (2016). Therapeutic targeting of splicing in cancer. Nat Med.

[18] Seiler M, Yoshimi A, Darman R, Chan B, Keaney G, Thomas M (2018). H3B-8800, an orally available small-molecule splicing modulator, induces lethality in spliceosome-mutant cancers. Nat Med.

[19] Yarchoan M, Hopkins A, Jaffee EM (2017). Tumor mutational burden and response rate to PD-1 inhibition. N Engl J Med.

[20] Büttner R, Longshore JW, López-Ríos F, Merkelbach-Bruse S, Normanno N, Rouleau E (2019). Implementing TMB measurement in clinical practice: considerations on assay requirements. ESMO Open.

[21] Chan TA, Yarchoan M, Jaffee E, Swanton C, Quezada SA, Stenzinger A (2019). Development of tumor mutation burden as an immunotherapy biomarker: utility for the oncology clinic. Ann Oncol.

[22] Matsushita H, Vesely MD, Koboldt DC, Rickert CG, Uppaluri R, Magrini VJ (2012). Cancer exome analysis reveals a T-cell-dependent mechanism of cancer immunoediting. Nature..

[23] Cohen CJ, Rosenberg SA, Robbins PF, Cohen CJ, Gartner JJ, Horovitz-fried M (2015). Isolation of neoantigen-specific T cells from tumor and peripheral lymphocytes. J Clin Invest.

[24] Van Allen EM, Miao D, Schilling B, Shukla SA, Blank C, Zimmer L (2015). Genomic correlates of response to CTLA-4 blockade in metastatic melanoma. Science..

[25] McDermott DF, Huseni MA, Atkins MB, Motzer RJ, Rini BI, Escudier B (2018). Clinical activity and molecular correlates of response to atezolizumab alone or in combination with bevacizumab versus sunitinib in renal cell carcinoma. Nat Med.

[26] Miao D, Margolis CA, Gao W, Voss MH, Li W, Martini DJ (2018). Genomic correlates of response to immune checkpoint therapies in clear cell renal cell carcinoma. Science..

[27] M.D. Hellmann, L. Paz-Ares, R. Bernabe Caro, B. Zurawski, Kim SW, Carcereny Costa, K, Park, A, Alexandru, L. Lupinacci, E. de la Mora Jimenez, H. Sakai, I. Albert, A. Vergnenegre, S. Peters, K. Syrigos, F. Barlesi, M. Reck, H. Borghaei, J.R. Brahmer, K.J. O’Byrne, W.J. Geese, P. Bhagavatheeswaran, S.K. Rabindran, R. S and SSR. Nivolumab plus ipilimumab in advanced non–small-cell lung cancer. N Engl J Med. 2019;381:2020–2031. 10.1056/NEJMoa1910231 31562796

[28] Turajlic S, Litchfield K, Xu H, Rosenthal R, McGranahan N, Reading JL (2017). Insertion-and-deletion-derived tumour-specific neoantigens and the immunogenic phenotype: a pan-cancer analysis. Lancet Oncol.

[29] Smart AC, Margolis CA, Pimentel H, He MX, Miao D, Adeegbe D (2018). Intron retention is a source of neoepitopes in cancer. Nat Biotechnol.

[30] Kahles A, Lehmann K Van, Toussaint NC, Hüser M, Stark SG, Sachsenberg T, et al. Comprehensive analysis of alternative splicing across tumors from 8,705 patients. Cancer Cell; 2018;34:211–224.e6. 10.1016/j.ccell.2018.07.001 PMC9844097 30078747

[31] Shen L, Zhang J, Lee H, Batista MT, Johnston SA (2019). RNA transcription and splicing errors as a source of cancer frameshift neoantigens for vaccines. Sci Rep.

[32] Seki M, Katsumata E, Suzuki A, Sereewattanawoot S, Sakamoto Y, Mizushima-Sugano J (2018). Evaluation and application of RNA-Seq by MinION. DNA Res.

[33] Li H (2018). Minimap2: pairwise alignment for nucleotide sequences. Bioinformatics..

[34] McGlincy NJ, Tan LY, Paul N, Zavolan M, Lilley KS, Smith CWJ (2010). Expression proteomics of UPF1 knockdown in HeLa cells reveals autoregulation of hnRNP A2/B1 mediated by alternative splicing resulting in nonsense-mediated mRNA decay. BMC Genomics.

[35] Wyman D, Balderrama-gutierrez G, Reese F, Jiang S, Rahmanian S, Zeng W, et al. A technology-agnostic long-read analysis pipeline for transcriptome discovery and quantification. bioRxiv. 2020;672931.

[36] Wyman D, Mortazavi A (2019). TranscriptClean : variant-aware correction of indels , mismatches and splice junctions in long-read transcripts. Bioinformatics..

[37] Mano H (2008). Non-solid oncogenes in solid tumors: EML4–ALK fusion genes in lung cancer. Cancer Sci.

[38] Yang W, Lee KW, Srivastava RM, Kuo F, Krishna C, Chowell D (2019). Immunogenic neoantigens derived from gene fusions stimulate T cell responses. Nat Med.

[39] Suzuki A, Suzuki M, Mizushima-Sugano J, Frith MC, Makałowski W, Kohno T (2017). Sequencing and phasing cancer mutations in lung cancers using a long-read portable sequencer. DNA Res.

[40] Cui Y, Irudayaraj J (2015). Inside single cells: quantitative analysis with advanced optics and nanomaterials. Wiley Interdiscip Rev Nanomed Nanobiotechnol.

[41] Pellagatti A, Armstrong RN, Steeples V, Sharma E, Repapi E, Singh S (2018). Impact of spliceosome mutations on RNA splicing in myelodysplasia: Dysregulated genes/pathways and clinical associations. Blood..

[42] Cuccurese M, Russo G, Russo A, Pietropaolo C (2005). Alternative splicing and nonsense-mediated mRNA decay regulate mammalian ribosomal gene expression. Nucleic Acids Res.

[43] Ni JZ, Grate L, Donohue JP, Preston C, Nobida N, O’Brien G (2007). Ultraconserved elements are associated with homeostatic control of splicing regulators by alternative splicing and nonsense-mediated decay. Genes Dev.

[44] Yoshihama M, Uechi T, Asakawa S, Kawasaki K, Kato S, Higa S (2002). The human ribosomal protein genes: sequencing and comparative analysis of 73 genes. Genome Res.

[45] Imamachi N, Salam KA, Suzuki Y, Akimitsu N (2017). A GC-rich sequence feature in the 3′ UTR directs UPF1-dependent mRNA decay in mammalian cells. Genome Res.

[46] Bailey TL, Boden M, Buske FA, Frith M, Grant CE, Clementi L (2009). MEME suite: tools for motif discovery and searching. Nucleic Acids Res.

[47] Wu G, Fan L, Edmonson MN, Shaw T, Boggs K, Easton J (2018). Inhibition of SF3B1 by molecules targeting the spliceosome results in massive aberrant exon skipping. RNA..

[48] Darman RB, Seiler M, Agrawal AA, Lim KH, Peng S, Aird D (2015). Cancer-associated SF3B1 hotspot mutations induce cryptic 3′ splice site selection through use of a different branch point. Cell Rep.

[49] Dolatshad H, Pellagatti A, Fernandez-Mercado M, Yip BH, Malcovati L, Attwood M (2015). Disruption of SF3B1 results in deregulated expression and splicing of key genes and pathways in myelodysplastic syndrome hematopoietic stem and progenitor cells. Leukemia..

[50] Papaemmanuil E, Cazzola M, Boultwood J, Malcovati L, Vyas P, Bowen D (2011). Somatic SF3B1 mutation in myelodysplasia with ring sideroblasts. N Engl J Med.

[51] Tang AD, Soulette CM, van Baren MJ, Hart K, Hrabeta-Robinson E, Wu CJ (2020). Full-length transcript characterization of SF3B1 mutation in chronic lymphocytic leukemia reveals downregulation of retained introns. Nat Commun.

[52] Wang D, Eraslan B, Wieland T, Hallström B, Hopf T, Zolg DP (2019). A deep proteome and transcriptome abundance atlas of 29 healthy human tissues. Mol Syst Biol.

[53] Lonsdale J, Thomas J, Salvatore M, Phillips R, Lo E, Shad S (2013). The Genotype-Tissue Expression (GTEx) project. Nat Genet.

[54] Lindeboom RGH, Supek F, Lehner B (2016). The rules and impact of nonsense-mediated mRNA decay in human cancers. Nat Genet.

[55] Zetoune AB, Fontanière S, Magnin D, Anczuków O, Buisson M, Zhang CX (2008). Comparison of nonsense-mediated mRNA decay efficiency in various murine tissues. BMC Genet.

[56] Zhao B, Pritchard JR (2019). Evolution of the nonsense-mediated decay pathway is associated with decreased cytolytic immune infiltration. PLoS Comput Biol.

[57] Wu CC, Beird HC, Andrew Livingston J, Advani S, Mitra A, Cao S (2020). Immuno-genomic landscape of osteosarcoma. Nat Commun.

[58] Hänzelmann S, Castelo R, Guinney J. GSVA: gene set variation analysis for microarray and RNA-Seq data. BMC Bioinformatics. 2013;14;1–15. https://bmcbioinformatics.biomedcentral.com/articles/10.1186/1471-2105-14-7. 10.1186/1471-2105-14-7 PMC3618321 23323831

[59] Bindea G, Mlecnik B, Tosolini M, Kirilovsky A, Waldner M, Obenauf AC (2013). Spatiotemporal dynamics of intratumoral immune cells reveal the immune landscape in human cancer. Immunity..

[60] Sabarinathan R, Piulats JM, Muntasell A (2018). A pan-cancer landscape of interactions between solid tumors and infiltrating immune cell populations. Clin Cancer Res.

[61] Lindeboom RGH, Vermeulen M, Lehner B, Supek F (2019). The impact of nonsense-mediated mRNA decay on genetic disease, gene editing and cancer immunotherapy. Nat Genet.

[62] Sakamoto Y, Xu L, Seki M, Yokoyama TT, Kasahara M, Kashima Y (2020). Long-read sequencing for non-small-cell lung cancer genomes. Genome Res.

[63] Bolger AM, Lohse M, Usadel B (2014). Genome analysis Trimmomatic : a flexible trimmer for Illumina sequence data. Bioinformatics..

[64] Langmead B, Salzberg SL (2012). Fast gapped-read alignment with Bowtie 2. Nat Methods.

[65] Dobin A, Davis CA, Schlesinger F, Drenkow J, Zaleski C, Jha S (2013). STAR: Ultrafast universal RNA-seq aligner. Bioinformatics..

[66] Li B, Dewey CN (2011). RSEM: accurate transcript quantification from RNA-seq data with or without a reference genome. BMC Bioinformatics..

[67] Li H, Durbin R (2010). Fast and accurate long-read alignment with Burrows – Wheeler transform. Bioinformatics..

[68] Mckenna A, Hanna M, Banks E, Sivachenko A, Cibulskis K, Kernytsky A (2010). The Genome Analysis Toolkit : a MapReduce framework for analyzing next-generation DNA sequencing data. Genome Res.

[69] Mclaren W, Gil L, Hunt SE, Riat HS, Ritchie GRS, Thormann A (2016). The Ensembl Variant Effect Predictor. Genome Biol.

[70] Untergasser A, Nijveen H, Rao X, Bisseling T, Geurts R, Leunissen JAM (2007). Primer3Plus, an enhanced web interface to Primer3. Nucleic Acids Res.

[71] Smit, AFA, Hubley, R, Green P. RepeatMasker Open-4.0. Available from: http://www.repeatmasker.org. Accessed 6 Oct 2020.

[72] Crooks GE, Hon G, Chandonia JM, Brenner SE (2004). WebLogo: a sequence logo generator. Genome Res.

[73] Schubert B, Mohr C, Sturm M, Feldhahn M, Kohlbacher O (2014). Sequence analysis OptiType : precision HLA typing from next-generation sequencing data. Bioinformatics..

[74] Jurtz V, Paul S, Andreatta M, Marcatili P, Peters B, Nielsen M (2017). NetMHCpan-4.0: improved peptide–MHC class I interaction predictions integrating eluted ligand and peptide binding affinity data. J Immunol.

[75] Masuda T, Tomita M, Ishihama Y. Phase transfer surfactant-aided trypsin digestion for membrane proteome analysis. J Proteome Res. 2008;7;731–40. https://pubs.acs.org/doi/abs/10.1021/pr700658q. 10.1021/pr700658q 18183947

[76] Rappsilber J, Mann M, Ishihama Y (2007). Protocol for micro-purification, enrichment, pre-fractionation and storage of peptides for proteomics using StageTips. Nat Protoc.

[77] Rappsilber J, Ishihama Y, Mann M (2003). Stop and go extraction tips for matrix-assisted laser desorption/ionization, nanoelectrospray, and LC/MS sample pretreatment in proteomics. Anal Chem.

[78] Cox J, Mann M (2008). MaxQuant enables high peptide identification rates, individualized p.p.b.-range mass accuracies and proteome-wide protein quantification. Nat Biotechnol.

[79] Pascolo S, Bervas N, Ure JM, Smith AG, Lemonnier FA, Pérarnau B (1997). HLA-A2.1–restricted education and cytolytic activity of CD8. J Exp Med..

[80] Grossman RL, Heath AP, Ferretti V, Varmus HE, Lowy DR, Kibbe WA (2016). Toward a shared vision for cancer genomic data. N Engl J Med.

[81] Mayakonda A, Lin D, Assenov Y, Plass C, Koeffler HP (2018). Maftools : efficient and comprehensive analysis of somatic variants in cancer. Genome Res.

[82] Maere S, Heymans K, Kuiper M (2005). BiNGO: a Cytoscape plugin to assess overrepresentation of gene ontology categories in biological networks. Bioinformatics..

[83] Tomislav S (2011). REVIGO summarizes and visualizes long lists of gene ontology terms. PLoS One.

[84] Subramanian A, Tamayo P, Mootha VK, Mukherjee S, Ebert BL (2005). Gene set enrichment analysis : a knowledge-based approach for interpreting genome-wide. PNAS..

[85] Kodama Y, Shumway M, Leinonen R (2012). The sequence read archive: explosive growth of sequencing data. Nucleic Acids Res.

[86] Suzuki Y. Aberrant transcript isoforms detected by full-length transcriptome sequencing as transcripts of potential neoantigens in non-small cell lung cancer. Datasets. DNA Data Bank of Japan. http://trace.ddbj.nig.ac.jp/DRASearch/submission?acc=DRA010214 (2020). 10.1186/s13059-020-02240-8 PMC7780684 33397462

[87] Suzuki Y. Aberrant transcript isoforms detected by full-length transcriptome sequencing as transcripts of potential neoantigens in non-small cell lung cancer. Datasets. DNA Data Bank of Japan. http://trace.ddbj.nig.ac.jp/DRASearch/submission?acc=DRA010215 (2020). 10.1186/s13059-020-02240-8 PMC7780684 33397462

[88] Kodama Y, Mashima J, Kosuge T, Katayama T, Fujisawa T, Kaminuma E (2015). The DDBJ Japanese genotype-phenotype archive for genetic and phenotypic human data. Nucleic Acids Res.

[89] Nakatsura T. Identifying aberrant splicing isoforms and potential neoantigens in non-small cell lung cancer. Datasets. Japanese Genotype-phenotype Archive. https://humandbs.biosciencedbc.jp/hum0236-v1 2020.

[90] Deutsch EW, Csordas A, Sun Z, Jarnuczak A, Perez-Riverol Y, Ternent T (2017). The ProteomeXchange consortium in 2017: supporting the cultural change in proteomics public data deposition. Nucleic Acids Res.

[91] Okuda S, Watanabe Y, Moriya Y, Kawano S, Yamamoto T, Matsumoto M (2017). jPOSTrepo : an international standard data repository for proteomes. Nucleic Acids Res.

[92] Moriya Y, Kawano S, Okuda S, Watanabe Y, Matsumoto M, Takami T (2019). The jpost environment: an integrated proteomics data repository and database. Nucleic Acids Res.

[93] Suzuki Y Aberrant splicing isoforms detected by full-length transcriptome sequencing as transcripts of potential neoantigens in non-small cell lung cancer. Datasets. Japan Proteome Standard Repository/Database. https://repository.jpostdb.org/entry/JPST000874 2020. 10.1186/s13059-020-02240-8 PMC7780684 33397462

