# Supplementary material for: Aberrant splicing isoforms detected by full-length transcriptome sequencing as transcripts of potential neoantigens in non-small cell lung cancer
Source: Genome Biol. 2021 Jan 4;22:9. doi: 10.1186/s13059-020-02240-8 (PMC7780684; doi:10.1186/s13059-020-02240-8)
Supplement: Supplementary file 12 — Additional file 12. Supplementary figures. [file 13059_2020_2240_MOESM12_ESM.pdf]

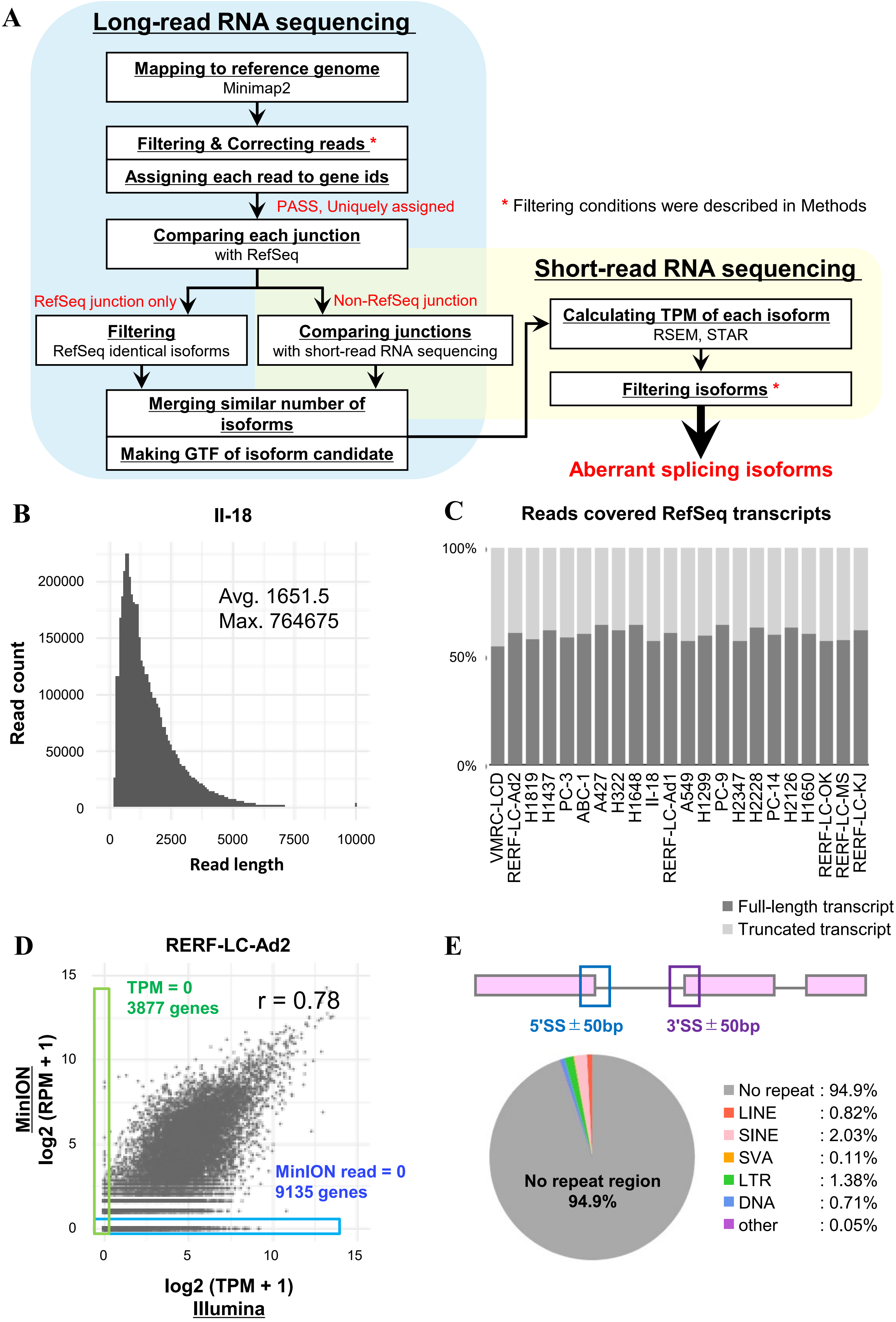
A
Long-read RNA sequencing
Mapping to reference genome
Minimap2
Filtering & Correcting reads *
Assigning each read to gene ids
PASS, Uniquely assigned
Comparing each junction
with RefSeq
RefSeq junction only
Non-RefSeq junction
Filtering
RefSeq identical isoforms
Comparing junctions
with short-read RNA sequencing
Merging similar number of isoforms
Making GTF of isoform candidate
* Filtering conditions were described in Methods
Short-read RNA sequencing
Calculating TPM of each isoform
RSEM, STAR
Filtering isoforms *
Aberrant splicing isoforms
B
II-18
Avg. 1651.5
Max. 764675
Read count
Read length
C
Reads covered RefSeq transcripts
100%
50%
0%
VMRC-LCD
RERF-LC-Ad2
H1819
H1437
PC-3
ABC-1
A427
H322
H1648
II-18
RERF-LC-Ad1
A549
H1299
PC-9
H2347
H2228
PC-14
H2126
H1650
RERF-LC-OK
RERF-LC-MS
RERF-LC-KJ
Full-length transcript
Truncated transcript
D
RERF-LC-Ad2
TPM = 0
3877 genes
r = 0.78
MinION read = 0
9135 genes
MinION
log2 (RPM + 1)
log2 (TPM + 1)
Illumina
E
5'SS ± 50bp
3'SS ± 50bp
No repeat region
94.9%
No repeat : 94.9%
LINE : 0.82%
SINE : 2.03%
SVA : 0.11%
LTR : 1.38%
DNA : 0.71%
other : 0.05%

Fig. S1. Overview of the results of MinION sequencing

(A) Analysis workflow for detecting splicing isoforms from MinION reads. Processes in the blue box were performed with long-read sequencing data, and processes in the yellow box were performed with short-read sequencing data. MinION reads were mapped to the reference genome using Minimap2, then filtered and corrected. Junctions in reads were compared with the RefSeq and short-read data. Merged isoform patterns were converted to a GTF file and used to calculate of the TPM of short-read RNA sequencing data. (B) Raw read length distribution of MinION sequencing in II-18. (C) The proportion of MinION reads covered the full-length of RefSeq transcripts. (D) A comparison of gene expression levels for RefSeq genes in MinION (RPM) and Illumina (TPM) in RERF-LC-Ad2. Both values + 1 were log2-transformed. The RPM of MinION reads were calculated from reads assigned to a single gene. (E) The proportion of repetitive elements detected around the novel splice sites in the isoforms.

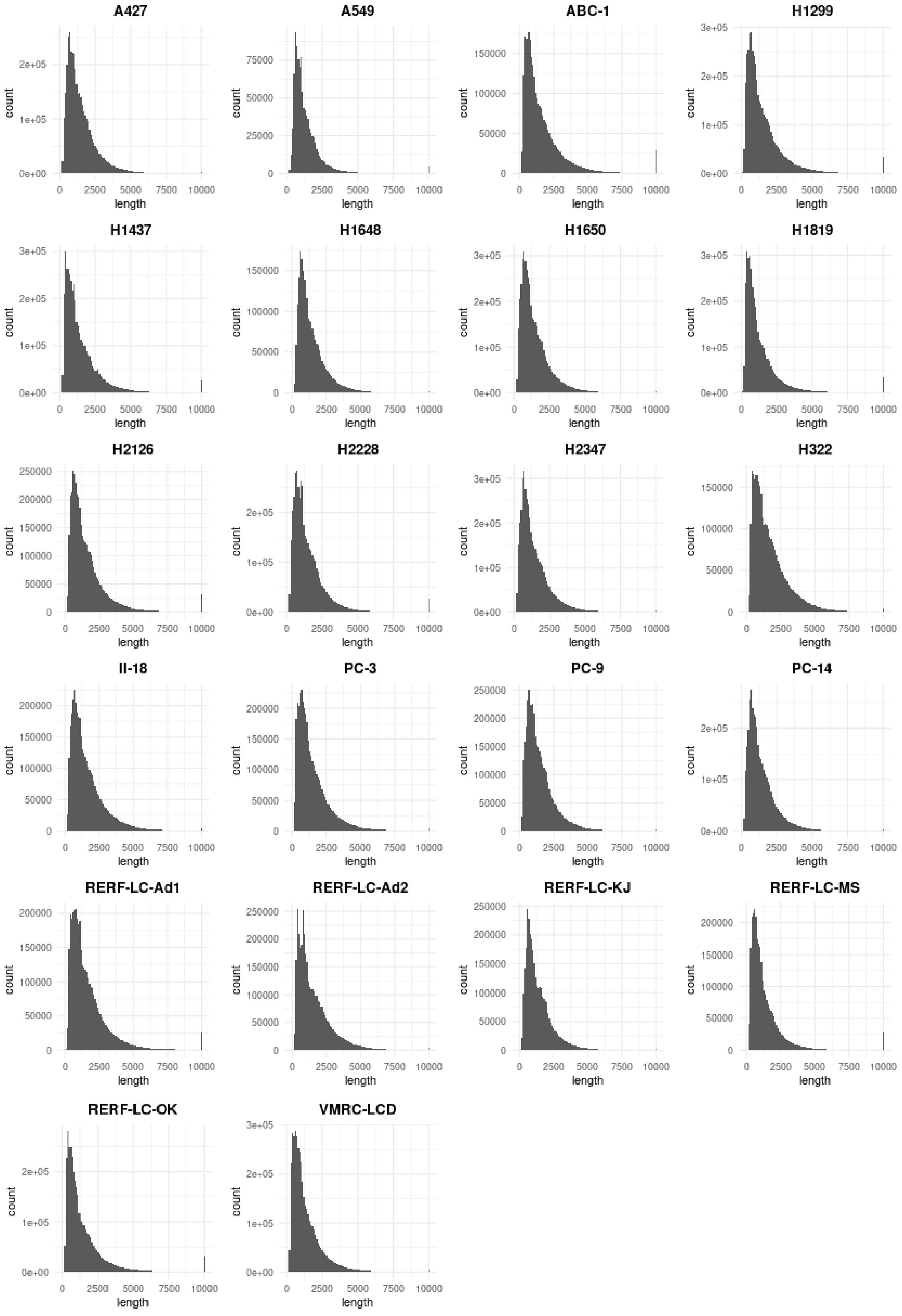
A427
A549
ABC-1
H1299
H1437
H1648
H1650
H1819
H2126
H2228
H2347
H322
II-18
PC-3
PC-9
PC-14
RERF-LC-Ad1
RERF-LC-Ad2
RERF-LC-KJ
RERF-LC-MS
RERF-LC-OK
VMRC-LCD
count
length

Fig. S2. Raw read length distribution of MinION sequencing in NSCLC cell lines

Raw read length distribution of MinION sequencing in each cell line.

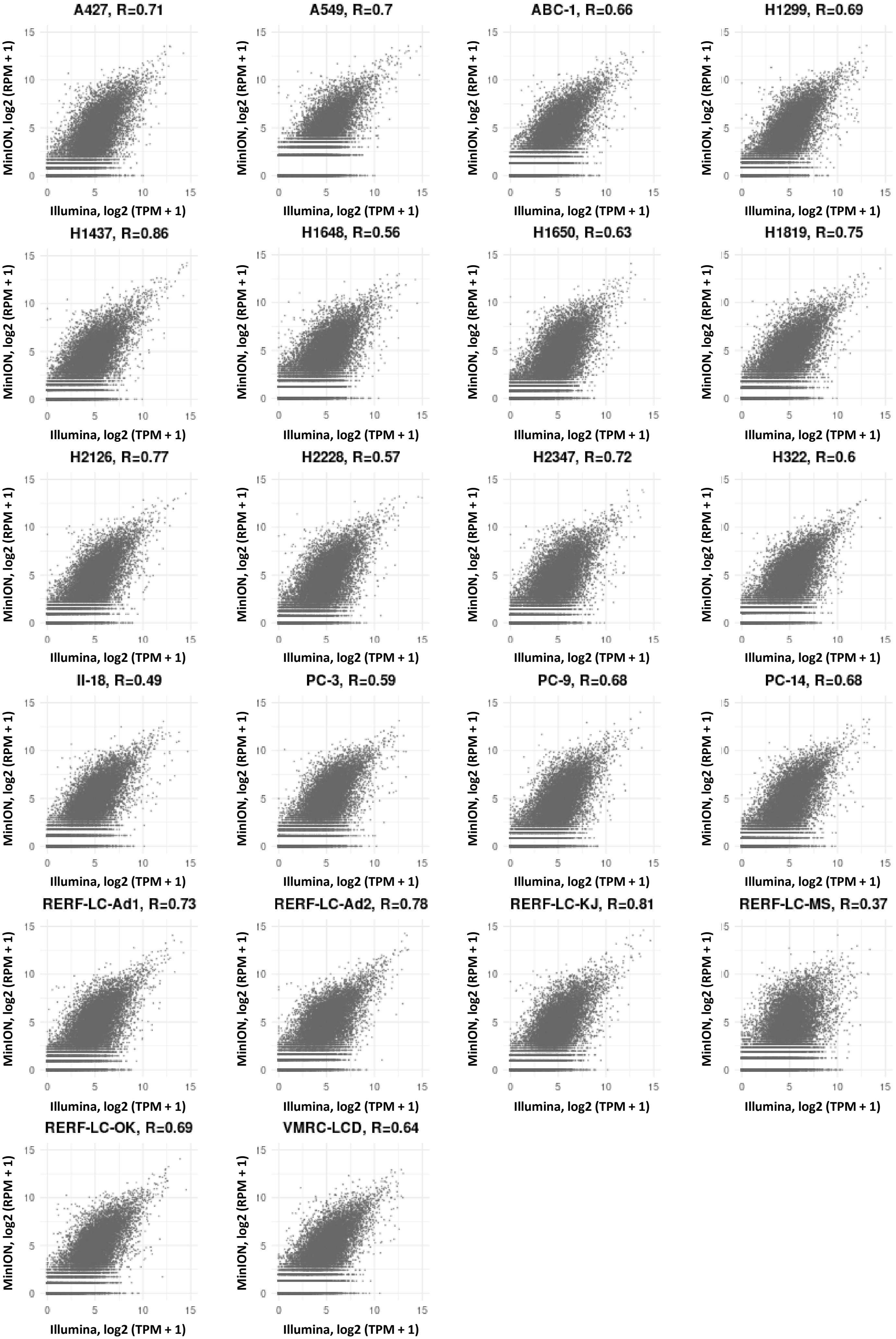
A427, R=0.71
A549, R=0.7
ABC-1, R=0.66
H1299, R=0.69
H1437, R=0.86
H1648, R=0.56
H1650, R=0.63
H1819, R=0.75
H2126, R=0.77
H2228, R=0.57
H2347, R=0.72
H322, R=0.6
II-18, R=0.49
PC-3, R=0.59
PC-9, R=0.68
PC-14, R=0.68
RERF-LC-Ad1, R=0.73
RERF-LC-Ad2, R=0.78
RERF-LC-KJ, R=0.81
RERF-LC-MS, R=0.37
RERF-LC-OK, R=0.69
VMRC-LCD, R=0.64
MinION, log2 (RPM + 1)
Illumina, log2 (TPM + 1)

Fig. S3. Comparison of the gene expression levels for RefSeq genes in MinION and Illumina
Comparison of gene expression levels for RefSeq genes in MinION (RPM) and Illumina (TPM). Both values + 1 were log2-transformed. RPM of MinION reads were calculated from reads assigned to a single gene.

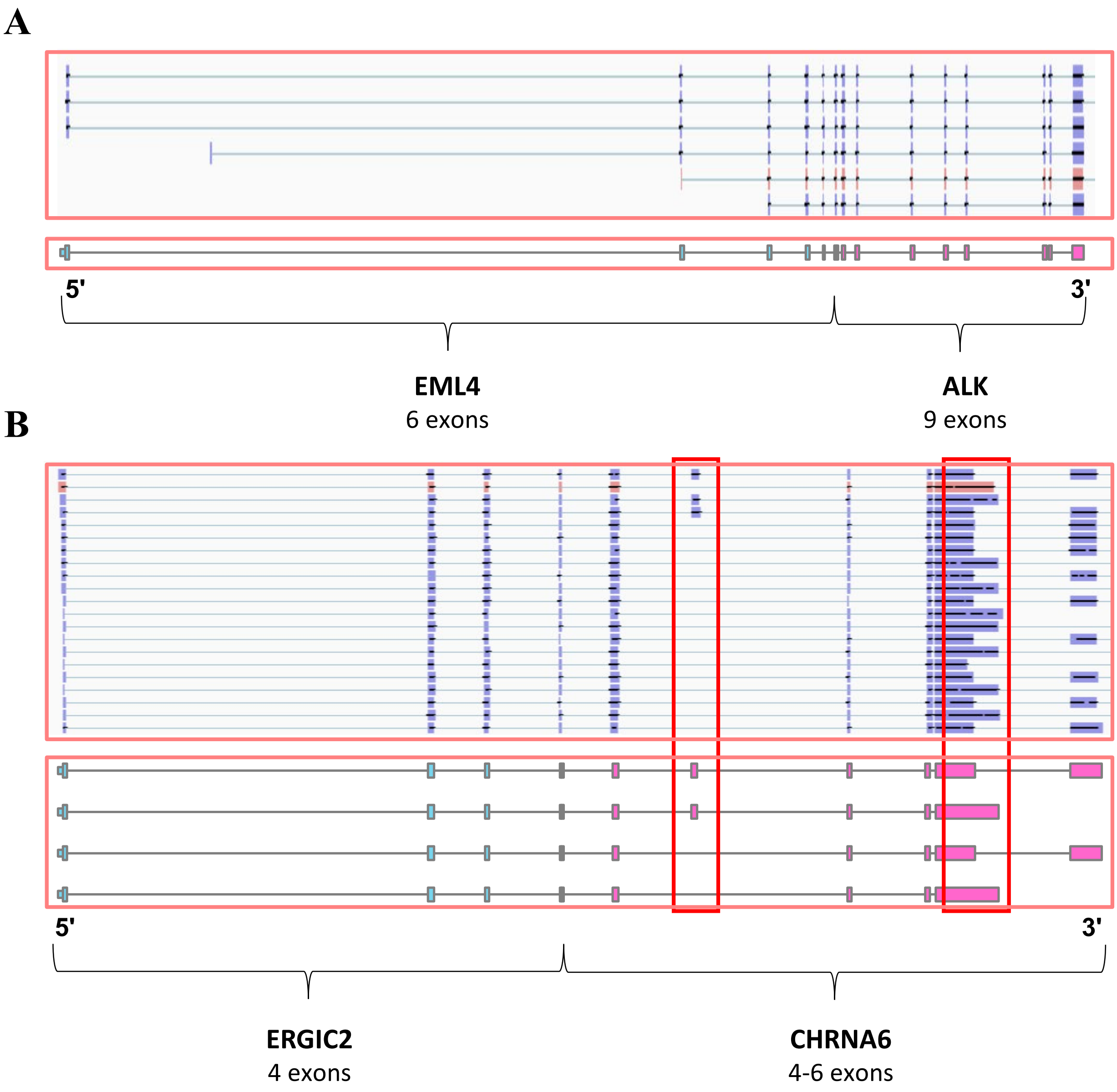

Fig. S4. Full-length structures of fusion-genes in cell lines
(A) The full-length structure of EML4-ALK in H2228. (B) The full-length structure of ERGIC2-CHRNA6 in H1437. Some MinION reads showed an unannotated exon and an alternative last exon in CHRNA6 (shown in red boxes).

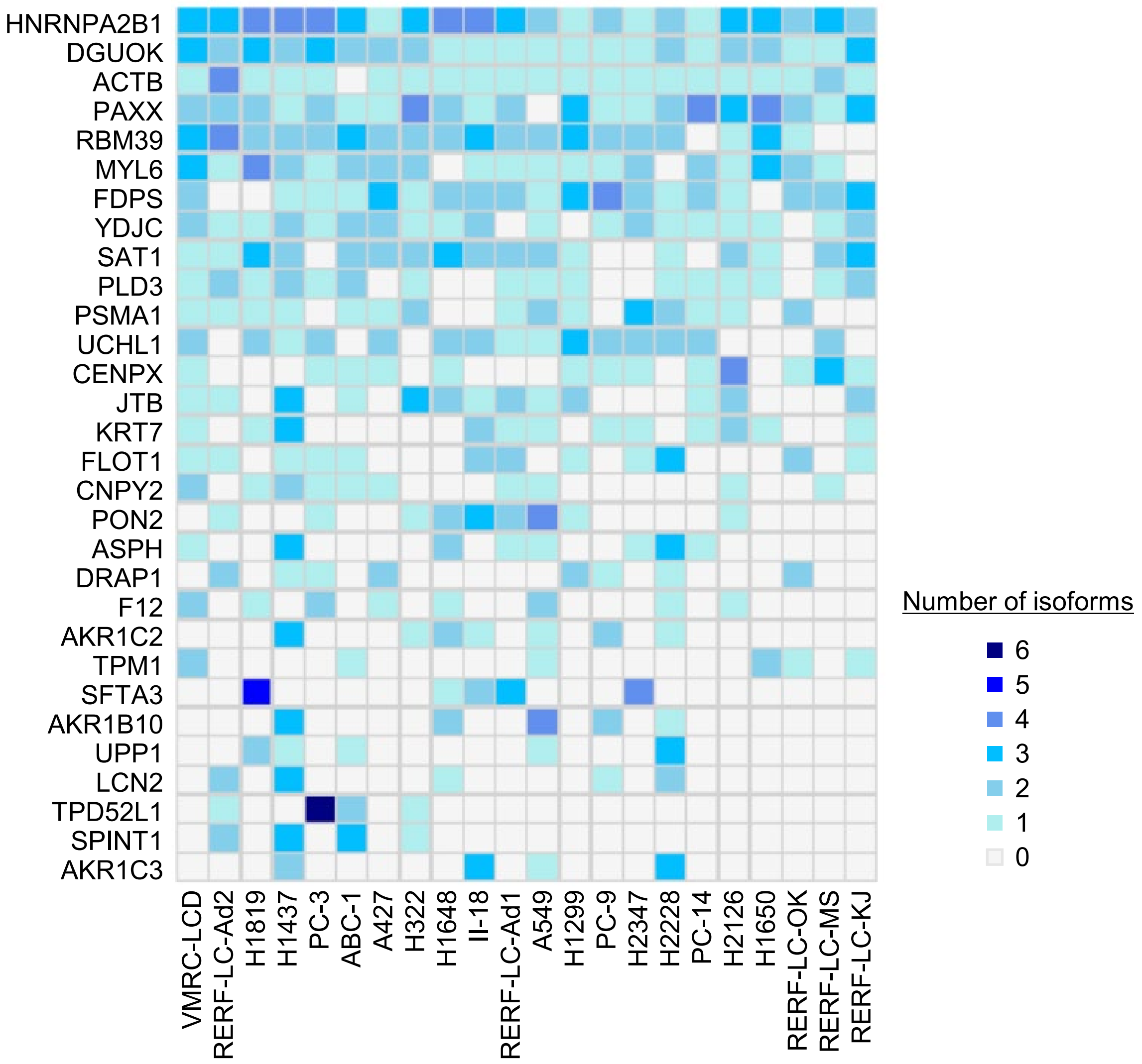

Fig. S5. Heatmap for genes with commonly expressed isoforms in cell lines

Heatmap showing the number of isoforms per gene that were expressed with at least five cell lines in common.

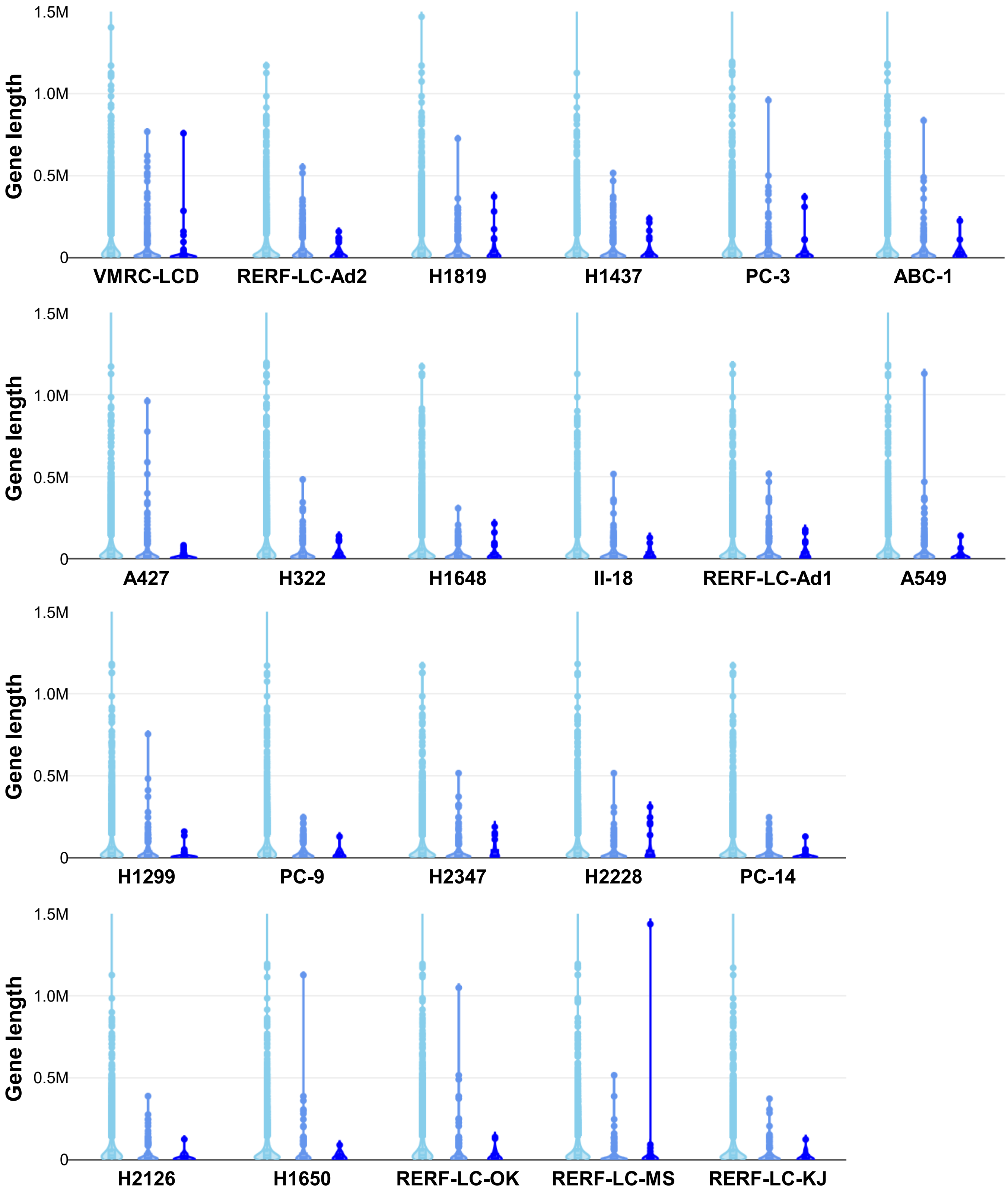

Fig. S6. Distribution of the lengths of genes with isoforms in cell lines

Violin plots of gene lengths for each cell line. Genes were divided into three groups: genes without isoforms, genes with one isoform, and genes with two or more isoforms (shown in light blue, pale blue, and blue, respectively).

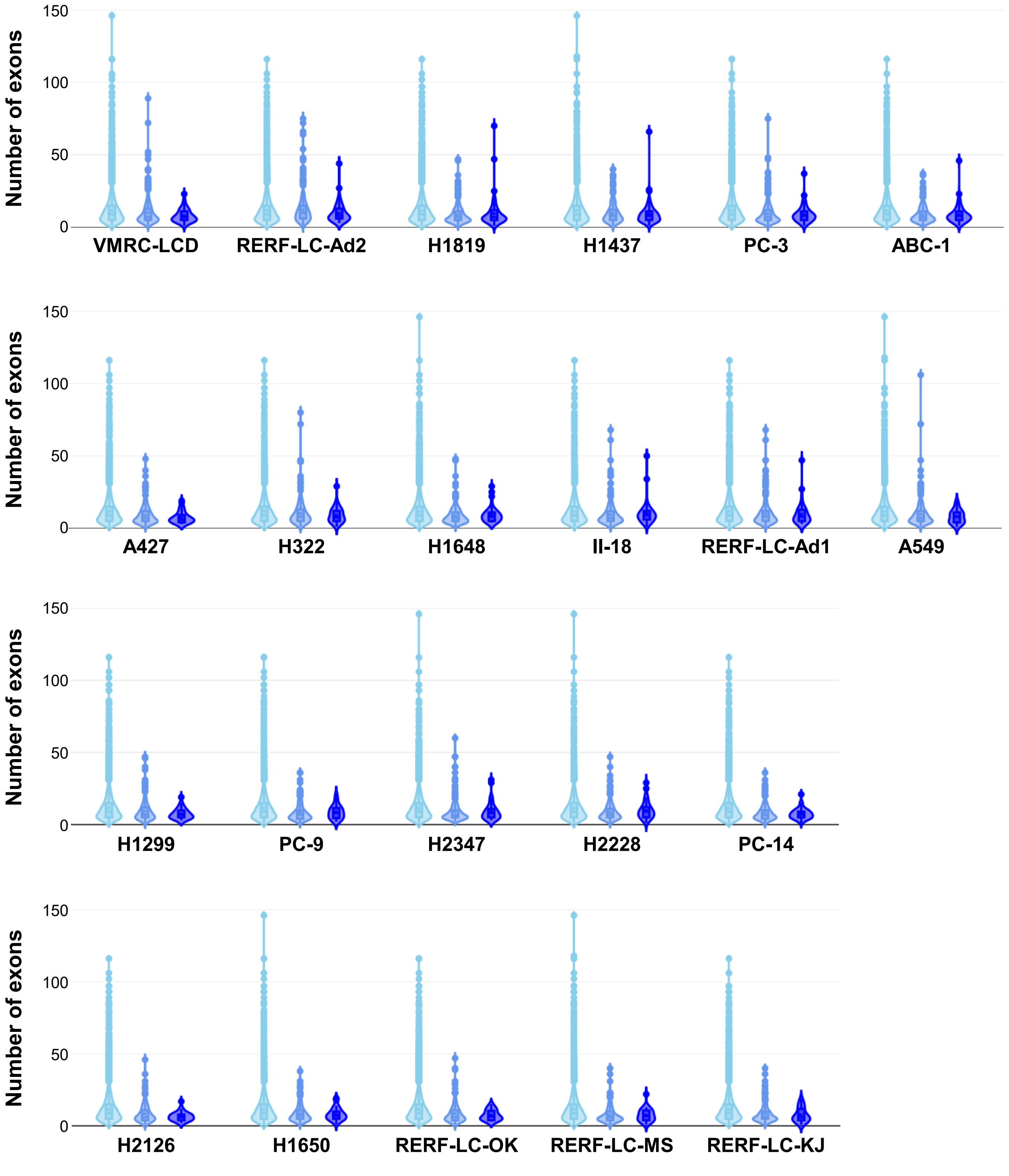

Fig. S7. Distribution of the number of exons in genes with isoforms in cell lines
Violin plots of the number of exons for each cell line. The plots are represented in a similar manner as described for **Fig. S6**.

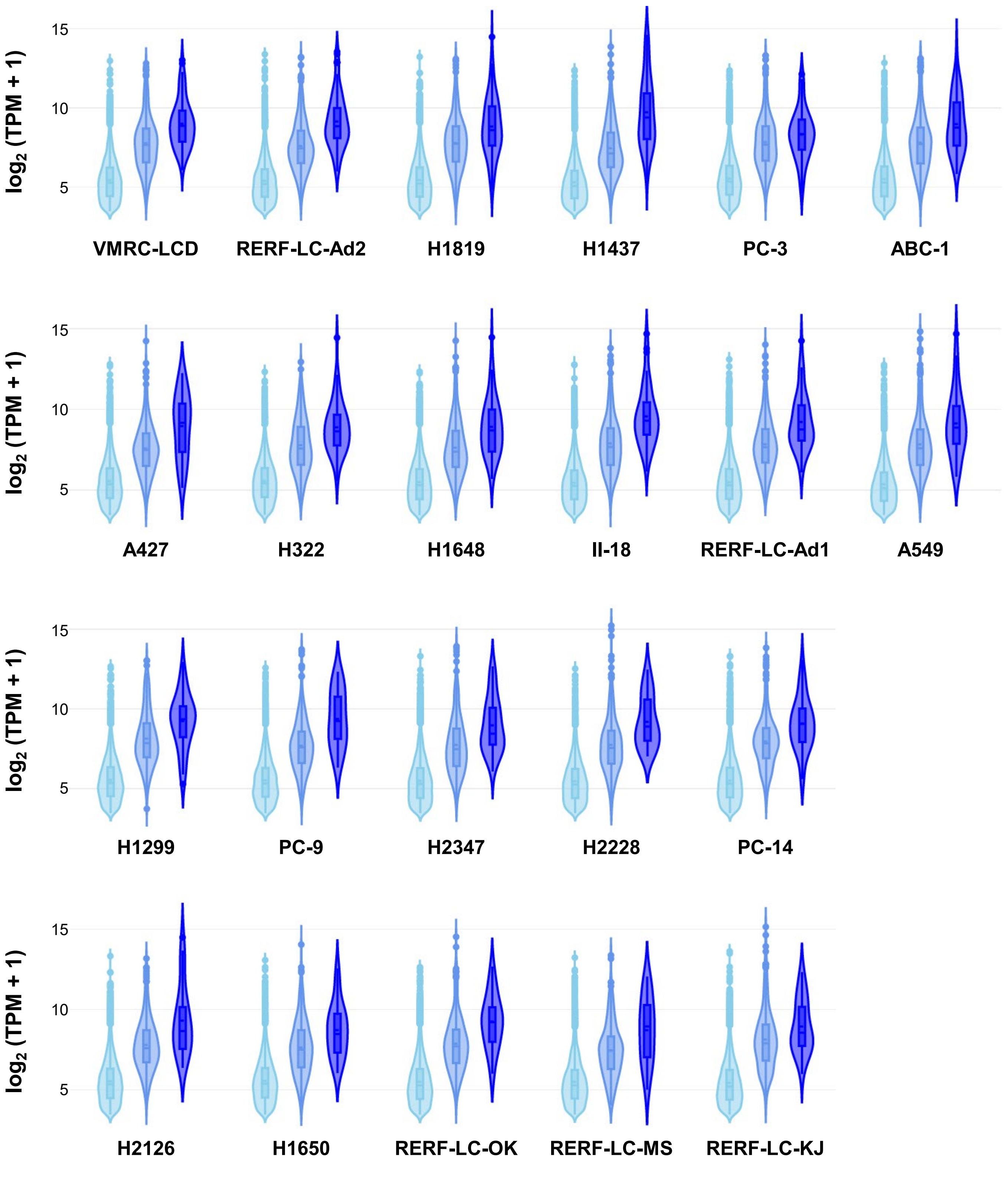

Fig. S8. Distribution of gene expression levels of genes with isoforms in cell lines
Violin plots of gene expression levels for each cell line. Plots are represented in a similar manner as described for **Fig. S6**.

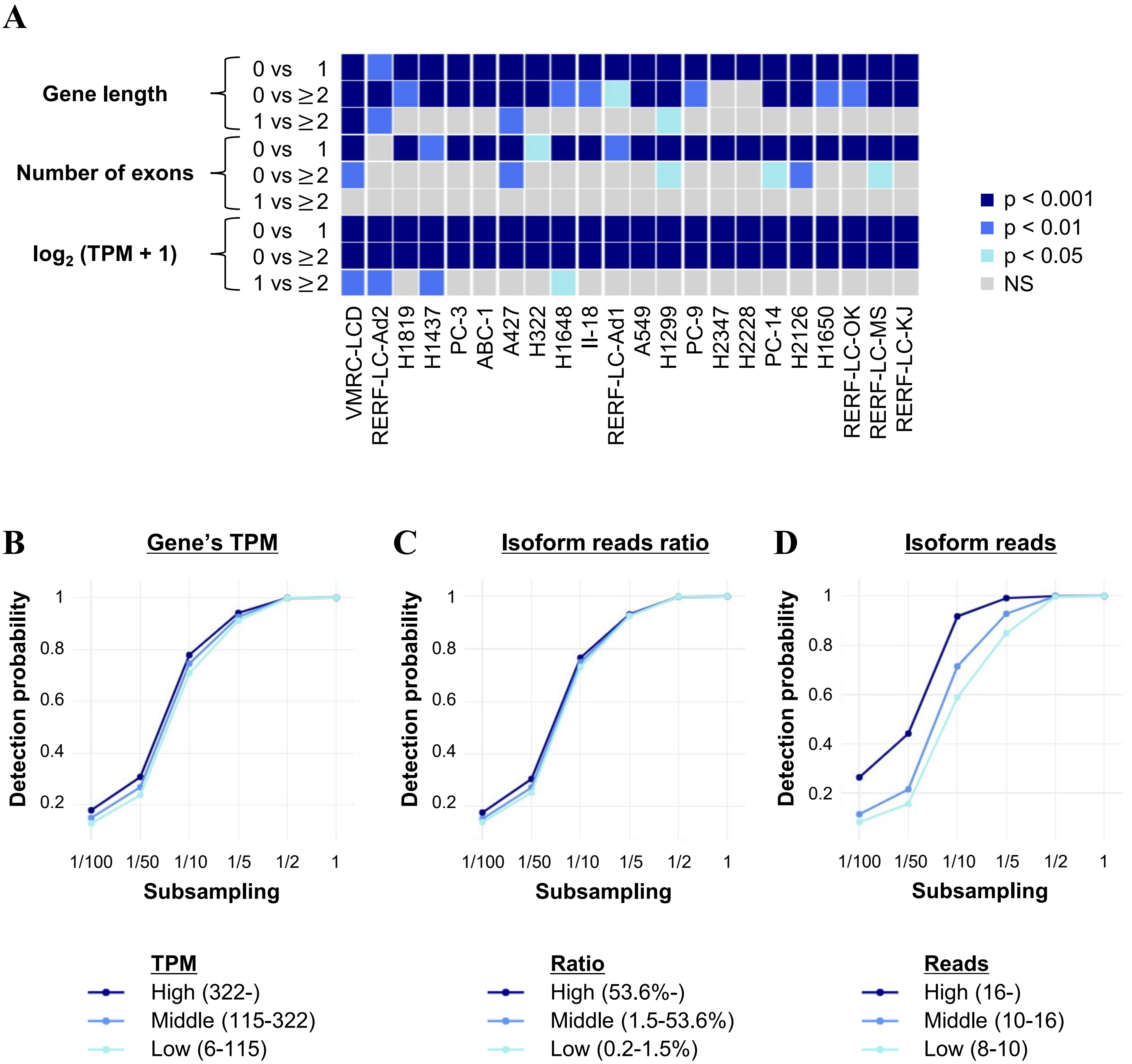

Fig. S9. Summary of the results of comparison for genes with isoforms in cell lines
(A) Heatmap showing the p-values for the distributions of lengths, exons, and expression levels of genes with isoforms for each cell line shown in **Supplementary Figs. 5-8**. P-values were calculated using Dunn–Bonferroni post-hoc tests. (B-D) The detection probability for each isoform during the subsampling of MinION reads in VMRC-LCD. We divided the isoforms into three classes, High, Middle, and Low for each of the three categories as follows; (B) TPM: calculated from short-read RNA sequencing data; (C) isoform-reads ratio: [the number of MinION reads covering the isoform pattern] / [the total number of MinION reads assigned to the gene]; (D) isoform-reads: the number of MinION reads covering the isoform pattern. The separation was made so that the numbers of the entries for each category should be approximately the same.

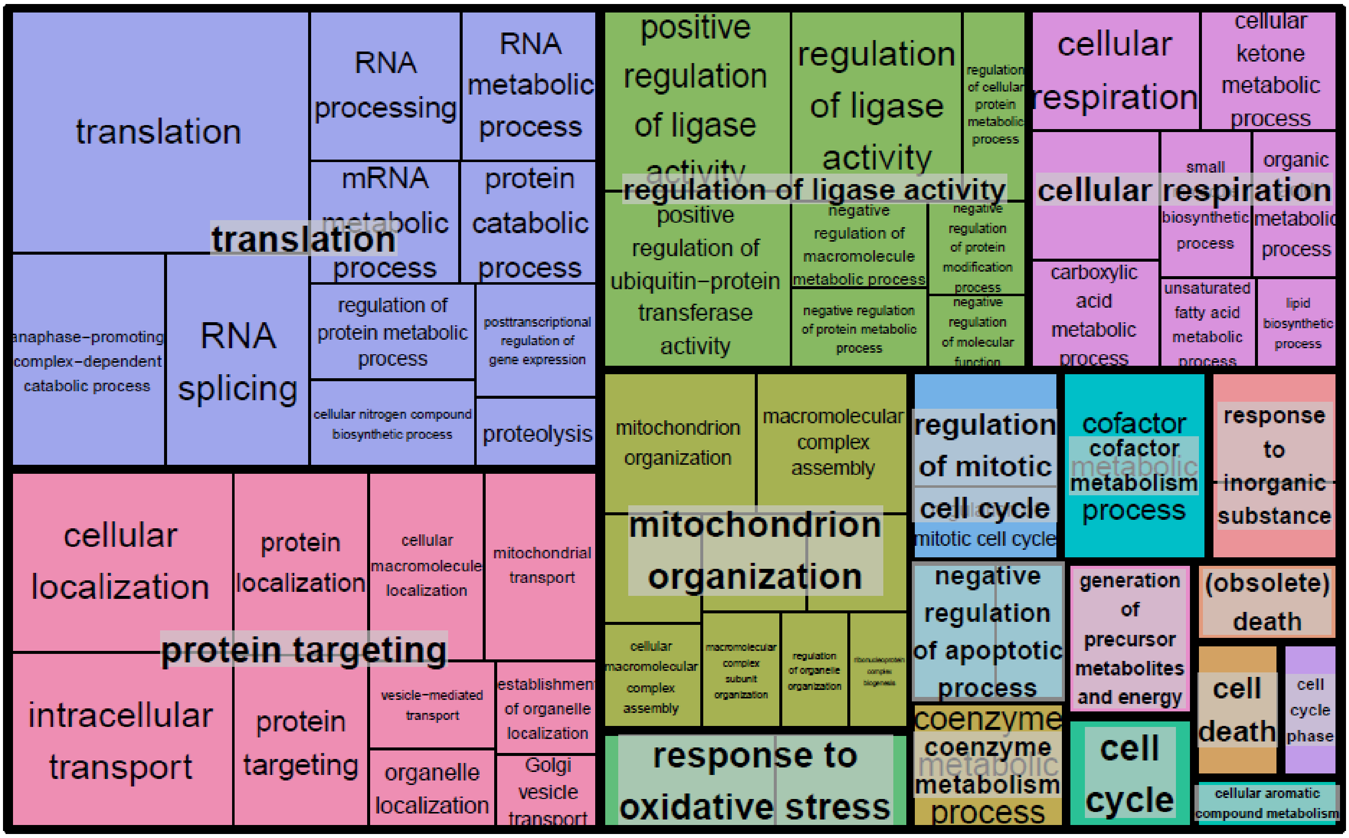

Fig. S10. Gene ontology analysis for genes with isoforms in cell lines

The results of gene ontology enrichment analysis were visualized using a REVIGO treemap. The panel sizes in the treemap are inversely proportional to the p-values.

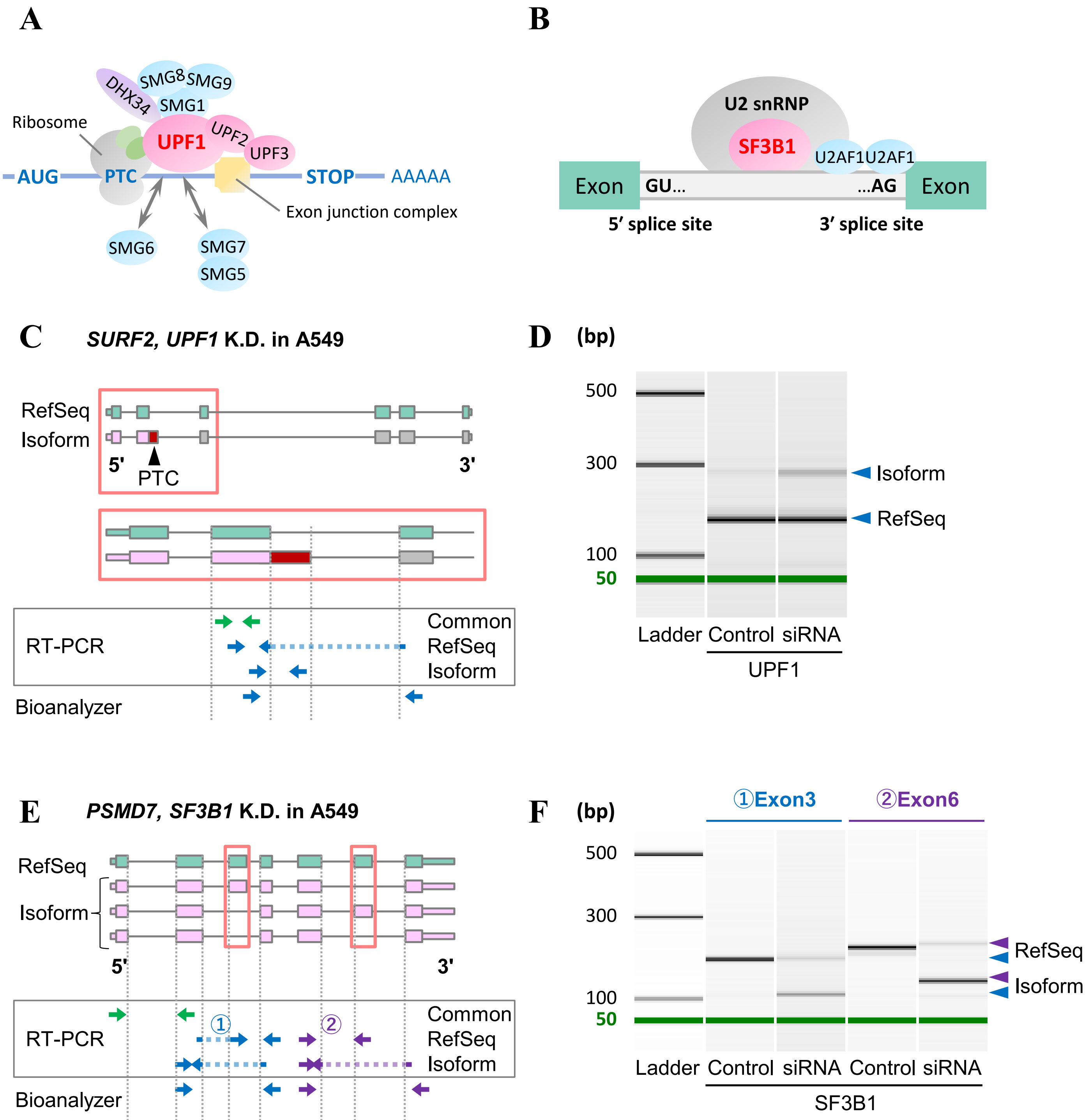

Fig. S11. The effect of *UPF1* and *SF3B1* knockdown in A549

(A) A simplified model of NMD complexes. UPF1 recognized transcripts containing PTC and recruited several NMD factors. (B) A simplified model of the U2 snRNA complex. U2 snRNA complex containing SF3B1 was recruited at the 3′ splice site. (C) The full-length structure of splicing isoforms of *SURF2* and primer sets used for detecting RefSeq- or isoform-specific regions in exon 2 (blue arrows) by RT-PCR and Bioanalyzer. Primers for common regions (green arrows) were used for normalization. (D) The results of the DNA electrophoresis of *UPF1*-depleted A549 using the Bioanalyzer. Arrows indicate RefSeq- or isoform-specific PCR products of *SURF2*. (E) The full-length structure of the splicing isoforms of *PSMD7* and primer sets for detecting RefSeq- or isoform-specific regions in exon 3 (blue arrows) and exon 6 (purple arrows) by RT-PCR and the Bioanalyzer. Primers for common regions were used for normalization. (F) The results of DNA electrophoresis in *SF3B1*-depleted A549 using the Bioanalyzer. Arrows indicate RefSeq- or isoform-specific PCR products of *PSMD7*.

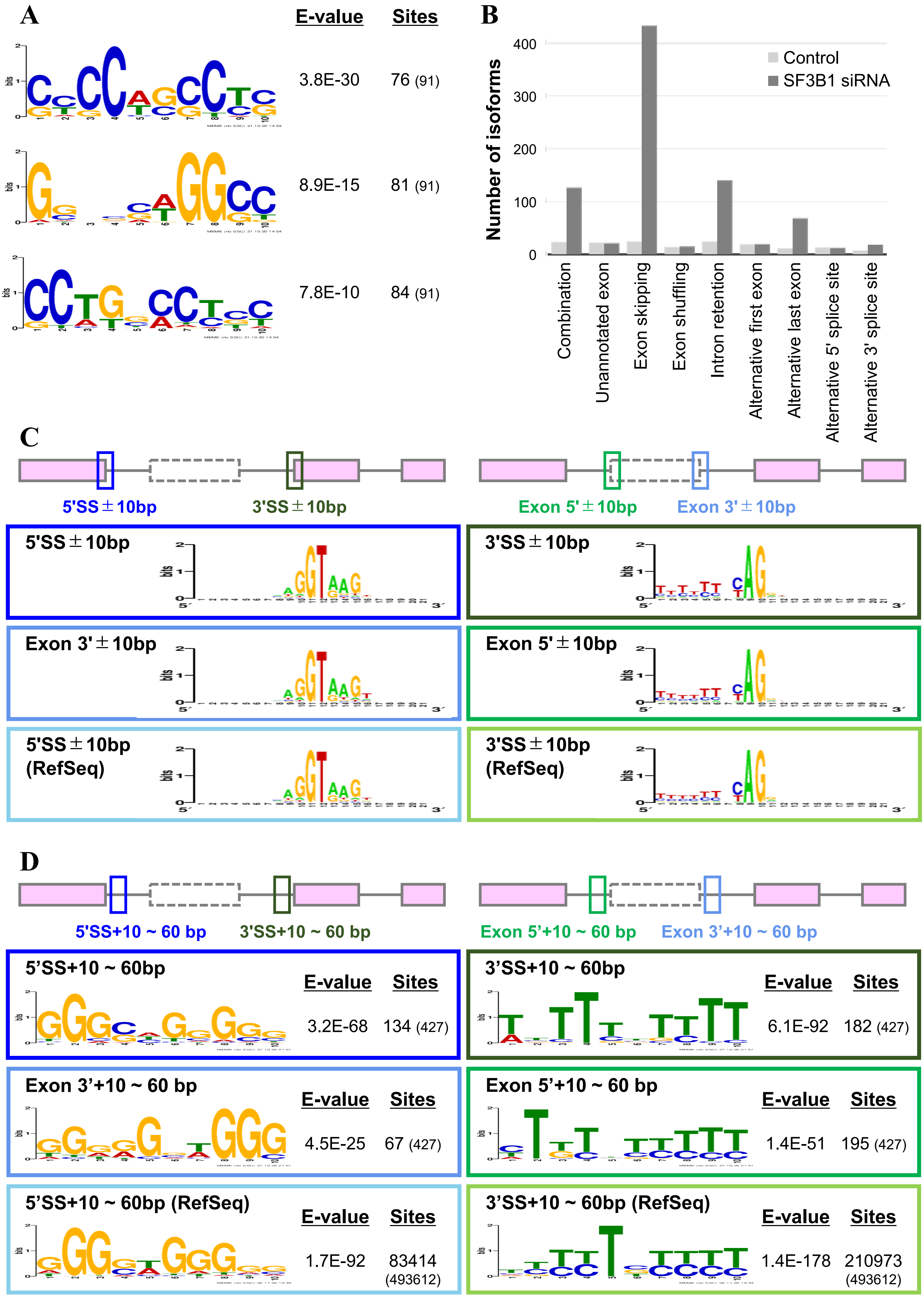
A
E-value
Sites
3.8E-30
76 (91)
8.9E-15
81 (91)
7.8E-10
84 (91)
B
Number of isoforms
400
300
200
100
0
Control
SF3B1 siRNA
Combination
Unannotated exon
Exon skipping
Exon shuffling
Intron retention
Alternative first exon
Alternative last exon
Alternative 5' splice site
Alternative 3' splice site
C
5'SS±10bp
3'SS±10bp
Exon 5'±10bp
Exon 3'±10bp
5'SS±10bp
3'SS±10bp
Exon 3'±10bp
Exon 5'±10bp
5'SS±10bp (RefSeq)
3'SS±10bp (RefSeq)
D
5'SS+10 ~ 60 bp
3'SS+10 ~ 60 bp
Exon 5'+10 ~ 60 bp
Exon 3'+10 ~ 60 bp
5'SS+10 ~ 60bp
E-value
Sites
3.2E-68
134 (427)
3'SS+10 ~ 60bp
E-value
Sites
6.1E-92
182 (427)
Exon 3'+10 ~ 60 bp
E-value
Sites
4.5E-25
67 (427)
Exon 5'+10 ~ 60 bp
E-value
Sites
1.4E-51
195 (427)
5'SS+10 ~ 60bp (RefSeq)
E-value
Sites
1.7E-92
83414 (493612)
3'SS+10 ~ 60bp (RefSeq)
E-value
Sites
1.4E-178
210973 (493612)

Fig. S12. Characterization for isoforms in *UPF1*- or *SF3B1*-depleted A549
(A) Overrepresented motifs in 3′ UTR of isoforms in *UPF1*-depleted A549 detected by MEME Suite. (B) The number of isoforms in *SF3B1*-depleted A549 classified into each splicing event. (C) Splicing consensus sequences detected in skipping exons or adjacent junctions of exon-skipping isoforms in *SF3B1*-depleted A549. (D) Overrepresented motifs in skipping exons or adjacent junctions of exon-skipping isoforms in *SF3B1*-depleted A549 detected by MEME Suite. T-rich motifs could be reflected polypyrimidine tract (PPT) where *U2AF* and PPT-binding proteins can bind. G-rich motifs may reflect potential binding sites for heterogeneous nuclear ribonucleoproteins regulating alternative splicing.

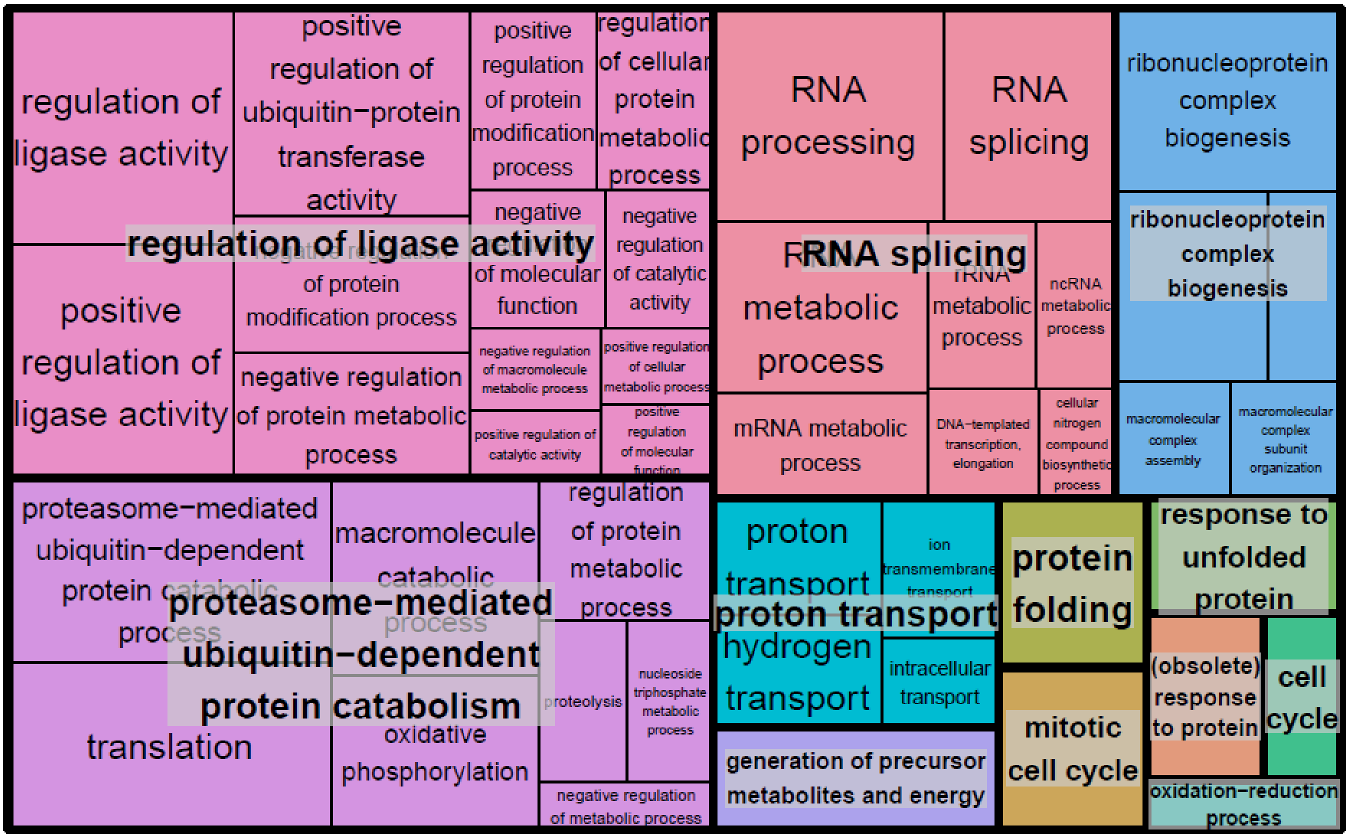

Fig. S13. Gene ontology analysis for genes with exon-skipping isoforms in *SF3B1*-depleted A549
The results of the gene ontology enrichment analysis were visualized using a REVIGO treemap. The panel sizes in the treemap are inversely proportional to the p-values.

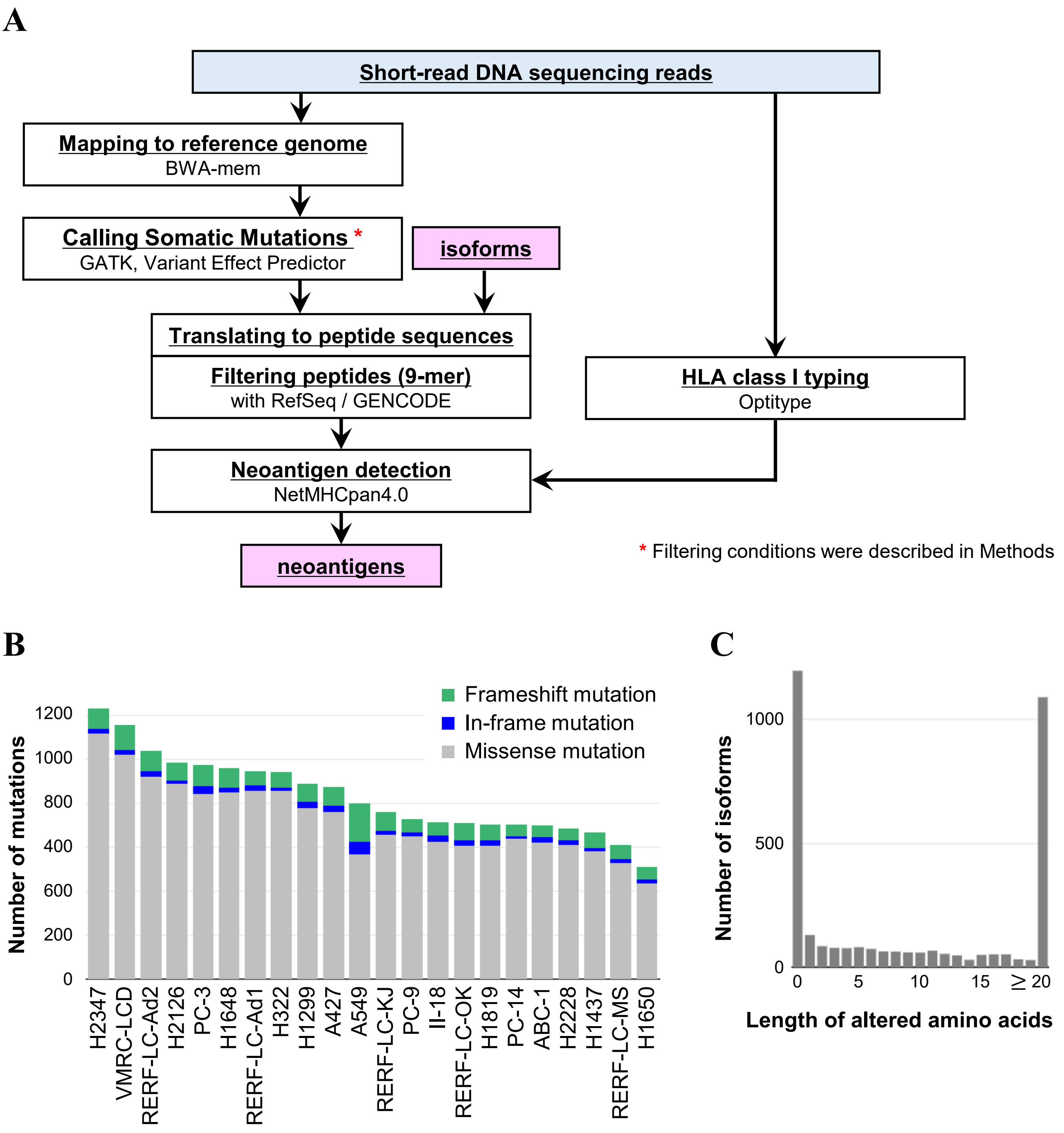

Fig. S14. Detection of neoantigen candidates

(A) The analysis workflow for detecting neoantigen candidates in combination with the long-read and short-read sequencing datasets. Reads from short-read DNA-seq were mapped to the reference genome using BWA-mem. Somatic mutations were called and annotated by GATK and VEP. HLA typing was performed using OptiType. After translating to peptides from aberrant splicing isoforms and somatic mutations, we extracted all possible 9-mer peptides that were not represented in RefSeq or in the GENCODE. The NetMHC score was calculated by combining the peptide sequences and HLA types. (B) The number of somatic mutations in each cell line. Missense mutations accounted for the majority of the detected mutations. (C) Comparison of the length of changed amino acids in aberrant splicing isoforms using RefSeq.

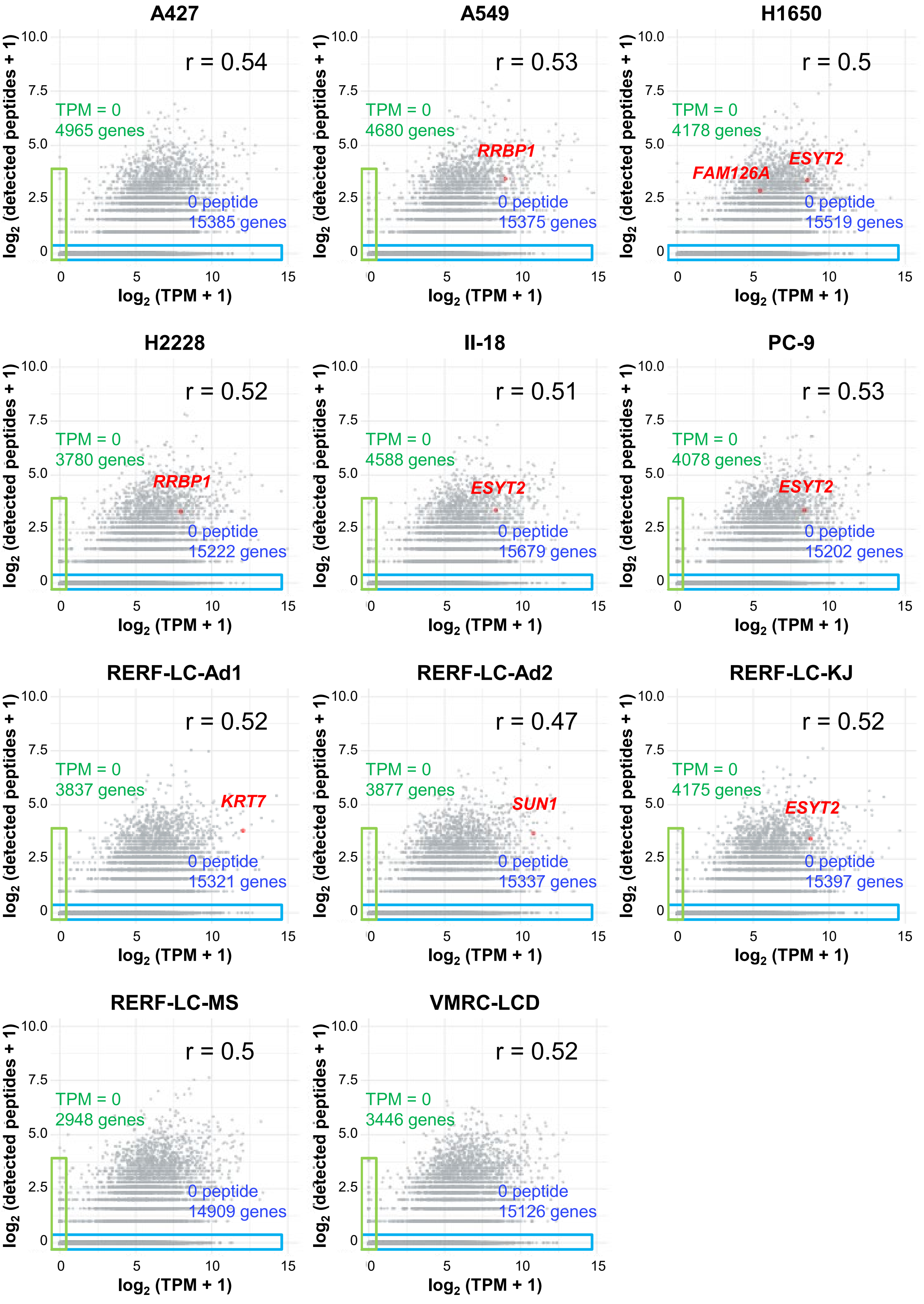

A427
r = 0.54
TPM = 0
4965 genes
0 peptide
15385 genes
A549
r = 0.53
TPM = 0
4680 genes
RRBP1
0 peptide
15375 genes
H1650
r = 0.5
TPM = 0
4178 genes
FAM126A
ESYT2
0 peptide
15519 genes
H2228
r = 0.52
TPM = 0
3780 genes
RRBP1
0 peptide
15222 genes
II-18
r = 0.51
TPM = 0
4588 genes
ESYT2
0 peptide
15679 genes
PC-9
r = 0.53
TPM = 0
4078 genes
ESYT2
0 peptide
15202 genes
RERF-LC-Ad1
r = 0.52
TPM = 0
3837 genes
KRT7
0 peptide
15321 genes
RERF-LC-Ad2
r = 0.47
TPM = 0
3877 genes
SUN1
0 peptide
15337 genes
RERF-LC-KJ
r = 0.52
TPM = 0
4175 genes
ESYT2
0 peptide
15397 genes
RERF-LC-MS
r = 0.5
TPM = 0
2948 genes
0 peptide
14909 genes
VMRC-LCD
r = 0.52
TPM = 0
3446 genes
0 peptide
15126 genes
log2 (detected peptides + 1)
log2 (TPM + 1)

Fig. S15. Comparisons between the results of short-read RNA sequencing and proteome analysis
Correlations between the gene expression levels calculated by short-read RNA sequencing (TPM) and the number of peptides detected by proteome analysis for each gene in the cell lines. Both values + 1 were log2-transformed. The red points represent genes with isoforms whose peptides were detected by proteome analysis. The green area highlights 0-TPM genes and the blue area highlights 0-peptide genes.

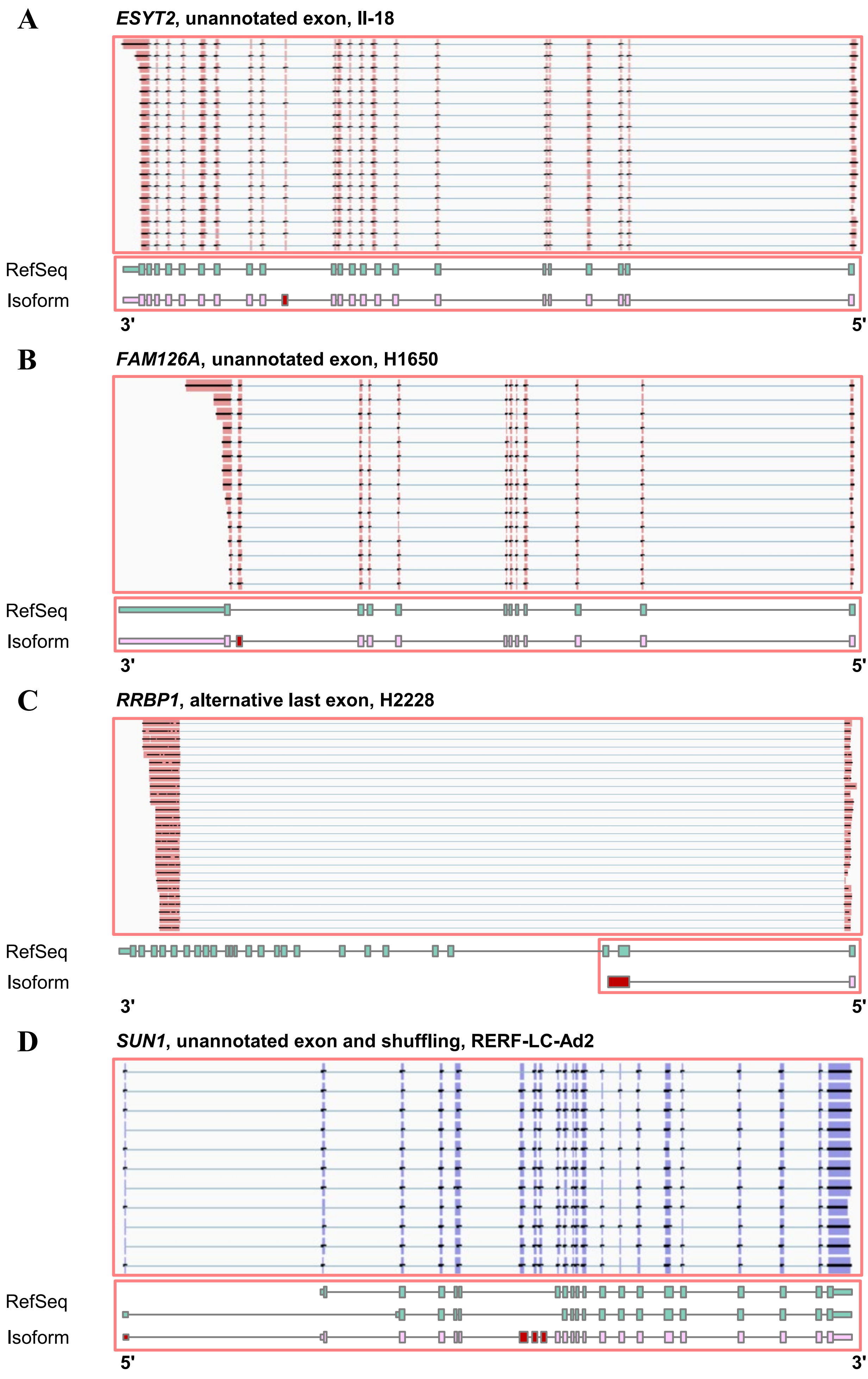
A
ESYT2, unannotated exon, II-18
RefSeq
Isoform
3'
5'
B
FAM126A, unannotated exon, H1650
RefSeq
Isoform
3'
5'
C
RRBP1, alternative last exon, H2228
RefSeq
Isoform
3'
5'
D
SUN1, unannotated exon and shuffling, RERF-LC-Ad2
RefSeq
Isoform
5'
3'

Fig. S16. Full-length structures of aberrant splicing isoforms detected by the proteome analysis
(A) The full-length structure of splicing isoforms of *ESYT2* in II-18. Some MinION reads showed an unannotated exon between exons 13 and 14. (B) The full-length structure of splicing isoforms of *FAM126A* in H1650. Some MinION reads showed an unannotated exon between exons 10 and 11. (C) The full-length structure of splicing isoforms of *RRBP1* in H2228. Some MinION reads showed an alternative last exon. (D) The full-length structure of splicing isoforms of *SUN1* in RERF-LC-Ad2. Some MinION reads showed exon shuffling of the first exon and unannotated exons between RefSeq exons 5 and 6.

A

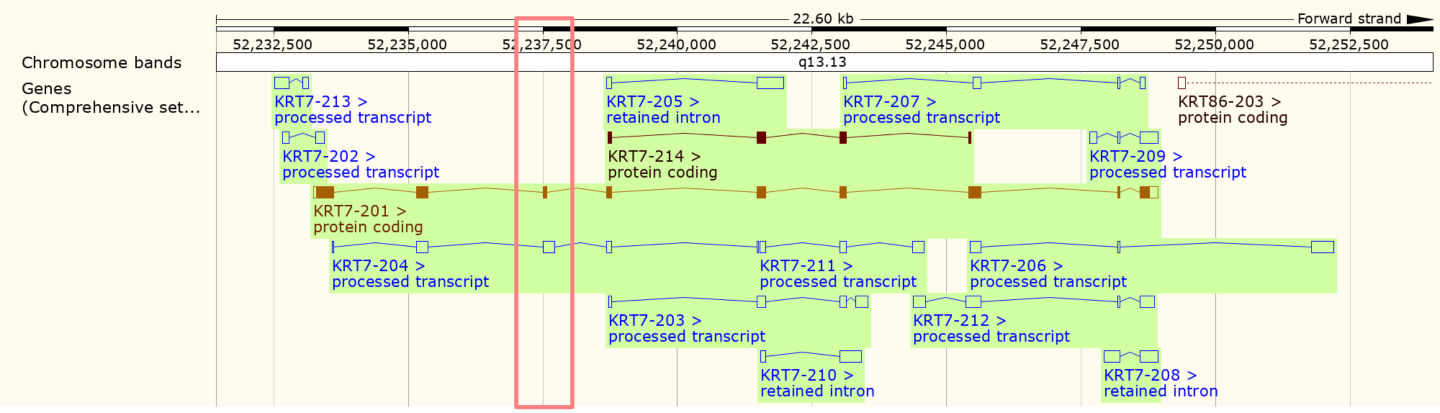

B

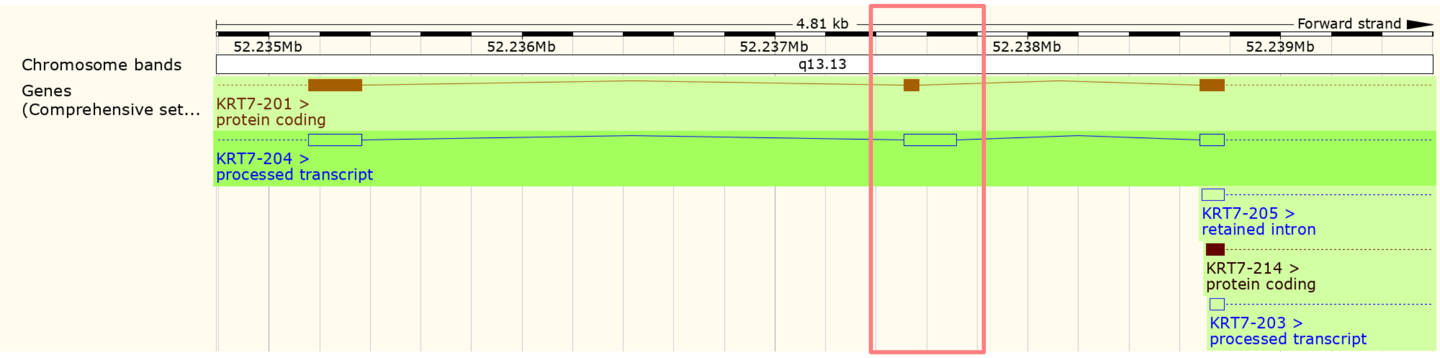

Fig. S17. *KRT7* transcripts registered in the Ensembl database

(A, B) Full-length structures of *KRT7* transcripts (A) and a magnified inset of alternative 5′ splice site region (B) in Ensembl. KRT7-204 (ENST00000547613) contained an isoform-specific junction (shown in **Figs. 4g** and **h**).

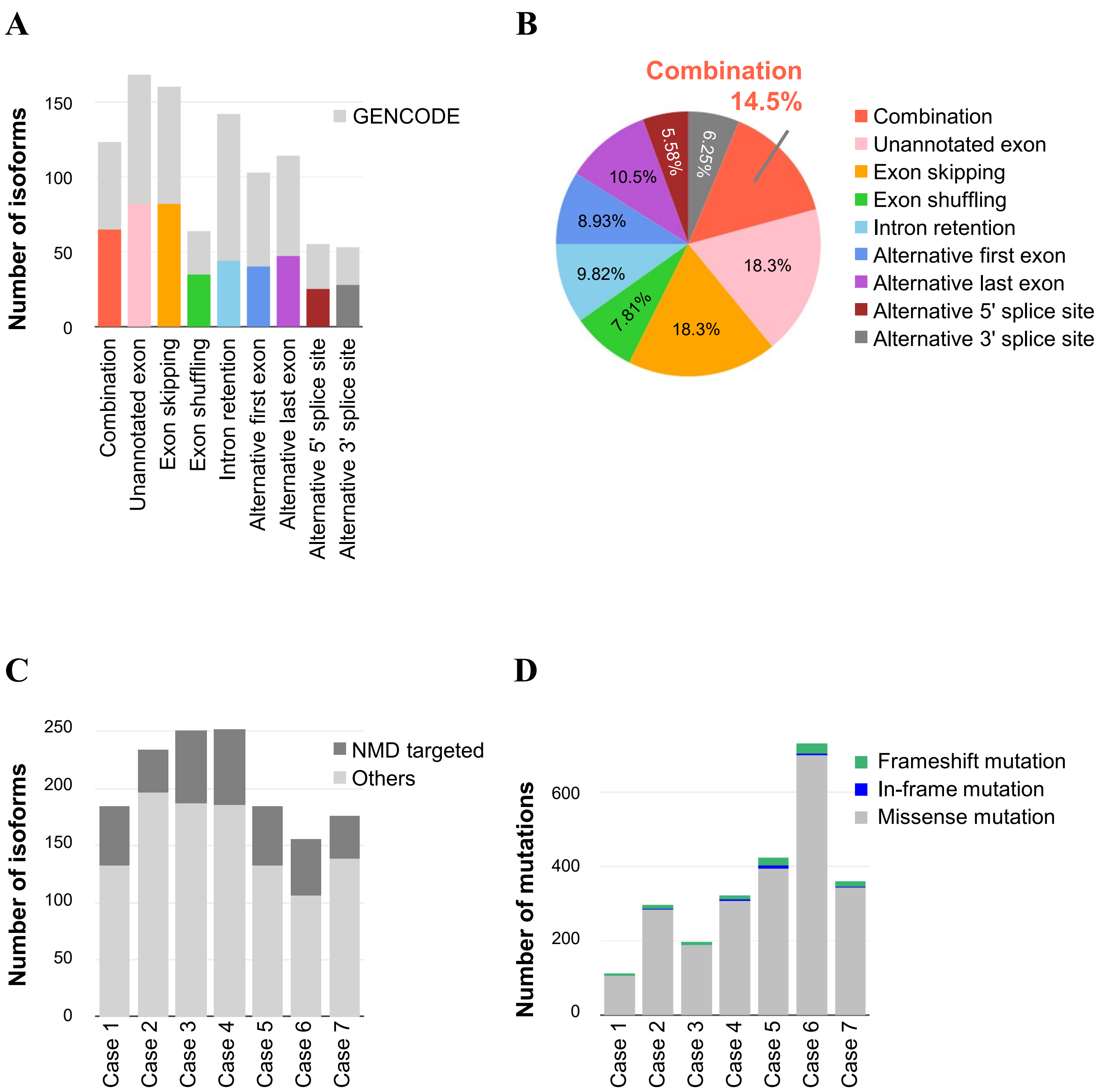

Fig. S18. Aberrant splicing isoforms detected in clinical specimens

(A) The number of isoforms classified for each splicing event. Light gray bars indicate isoforms represented in GENCODE, and other-colored bars indicate unannotated isoforms. (B) The proportion of splicing events in unannotated isoforms. Combination patterns accounted for 14.5%. (C) The number of PTC-containing splicing isoforms in each specimen are shown in gray. (D) The number of somatic mutations in each specimen.

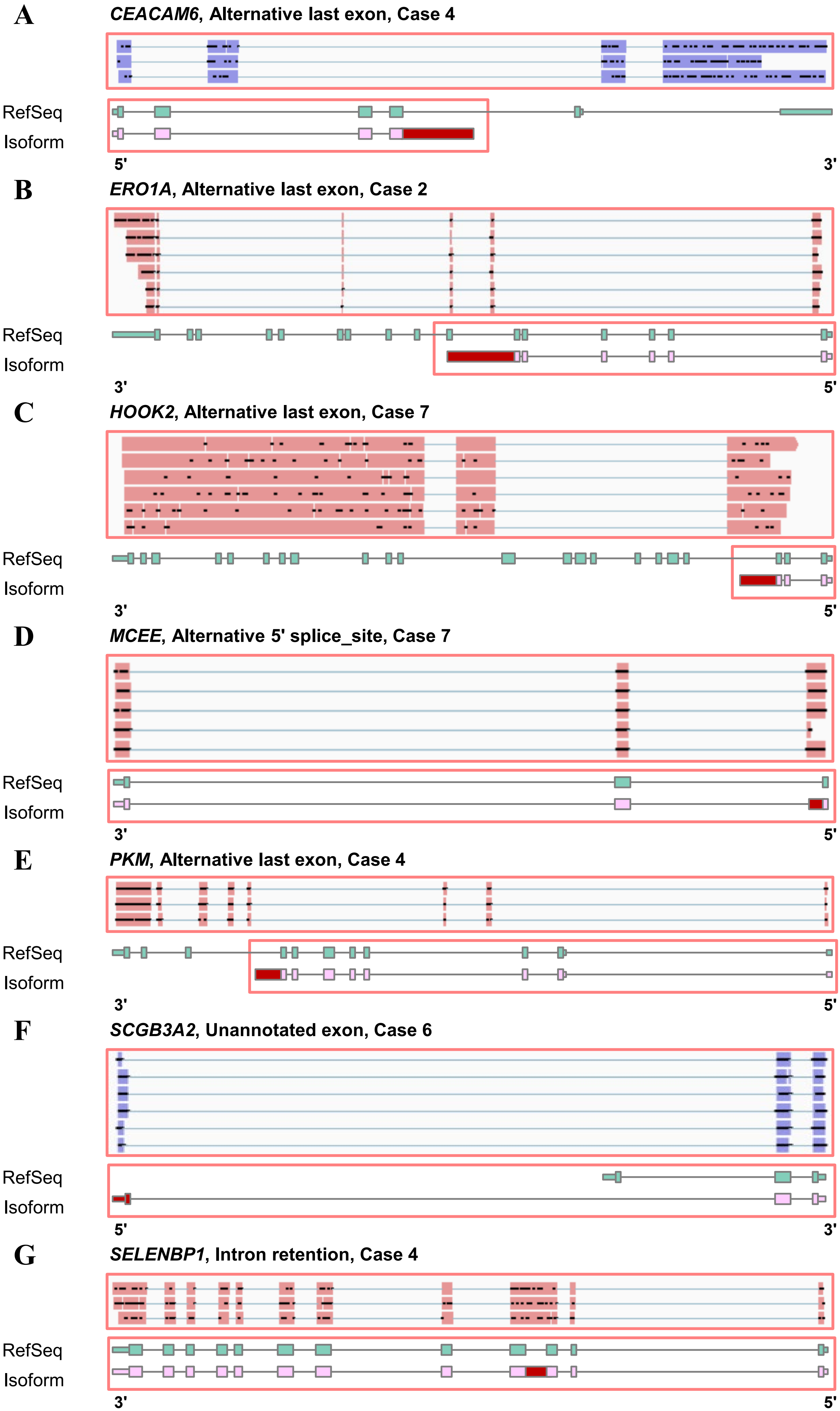

A
CEACAM6, Alternative last exon, Case 4
RefSeq
Isoform
5'
3'
B
ERO1A, Alternative last exon, Case 2
RefSeq
Isoform
3'
5'
C
HOOK2, Alternative last exon, Case 7
RefSeq
Isoform
3'
5'
D
MCEE, Alternative 5' splice_site, Case 7
RefSeq
Isoform
3'
5'
E
PKM, Alternative last exon, Case 4
RefSeq
Isoform
3'
5'
F
SCGB3A2, Unannotated exon, Case 6
RefSeq
Isoform
5'
3'
G
SELENBP1, Intron retention, Case 4
RefSeq
Isoform
3'
5'

H ***TMC4*, Alternative last exon, Case 1**

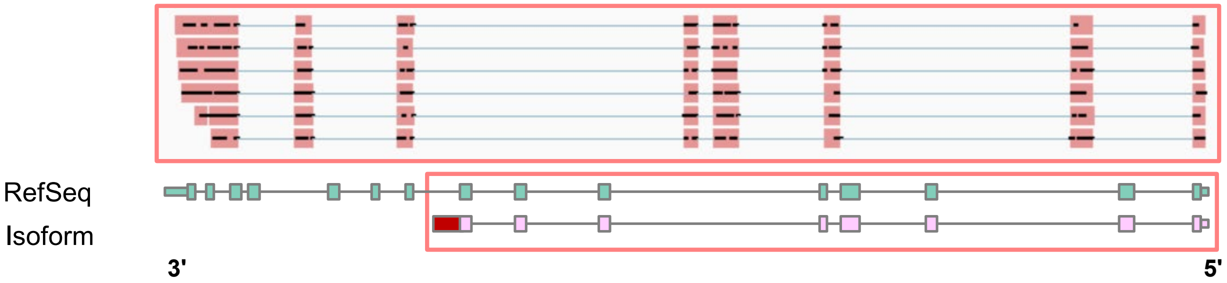

I ***TMEM45A*, Combination, Case 4**

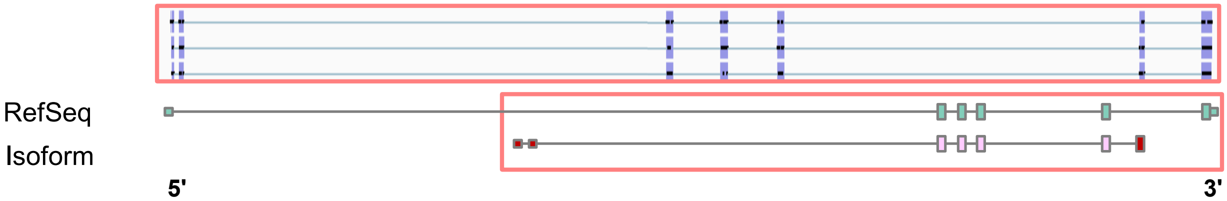

J ***TUFM*, Intron retention, Case 1**

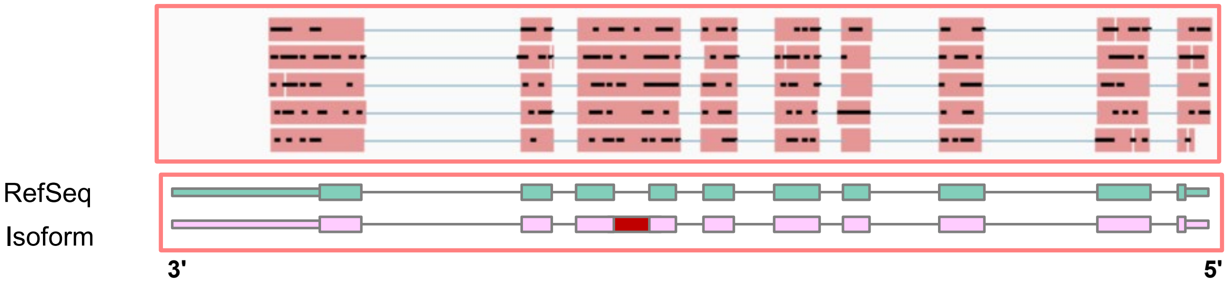

K ***UQCRB*, Alternative last exon, Case 3**

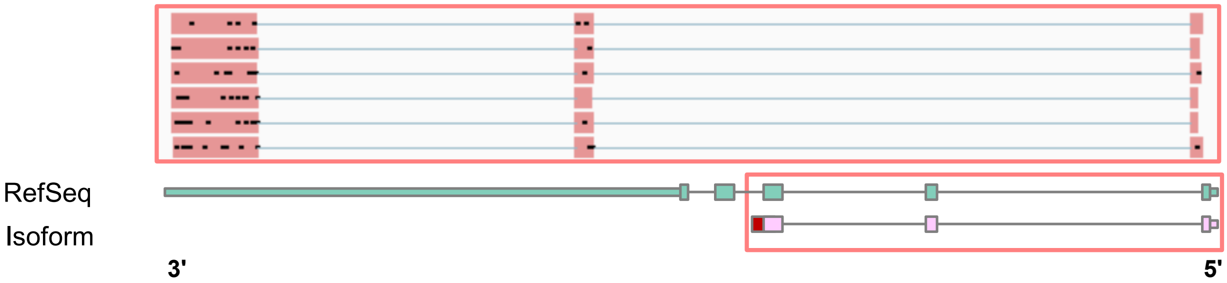

Fig. S19. Full-length structures of aberrant splicing isoforms tested in ELISpot assays
(A) The full-length structure of splicing isoforms of *CEACAM6* in case 4. Some MinION reads showed an alternative last exon. (B) The full-length structure of splicing isoforms of *ERO1A* in case 2. Some MinION reads showed an alternative last exon. (C) The full-length structure of splicing isoforms of *HOOK2* in case 7. Some MinION reads showed an alternative last exon. (D) The full-length structure of splicing isoforms of *MCEE* in case 7. Some MinION reads showed an alternative 5′ splice site in the first exon. (E) The full-length structure of splicing isoforms of *PKM* in case 4. Some MinION reads showed an alternative last exon. (F) The full-length structure of splicing isoforms of *SCGB3A2* in case 6. Some MinION reads showed an unannotated exon before the first exon. (G) The full-length structure of splicing isoforms of *SELENBP1* in case 4. Some MinION reads showed intron retention between exons 3 and 4. (H) The full-length structure of splicing isoforms of *TMC4* in case 1. Some MinION reads showed an alternative last exon. (I) The full-length structure of splicing isoforms of *TMEM45A* in case 4. Some MinION reads showed unannotated exons before exon 2 and after exon 5 of RefSeq. (J) The full-length structure of splicing isoforms of *TUFM* in case 1. Some MinION reads showed intron retention between exons 7 and 8. (K) The full-length structure of splicing isoforms of *UQCRB* in case 3. Some MinION reads showed an alternative last exon.

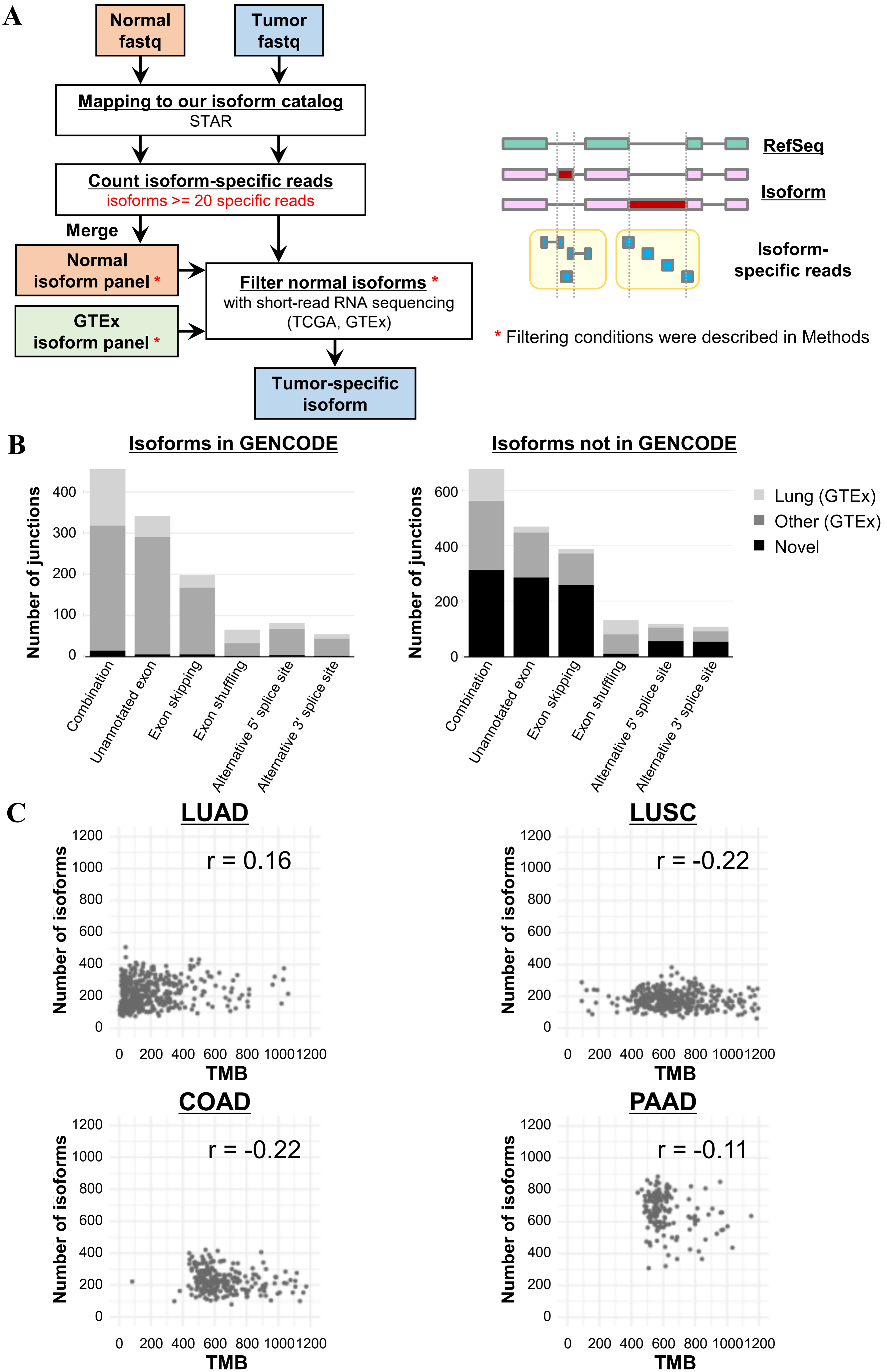
A
Normal fastq
Tumor fastq
Mapping to our isoform catalog
STAR
Count isoform-specific reads
isoforms >= 20 specific reads
Merge
Normal isoform panel *
GTEx isoform panel *
Filter normal isoforms *
with short-read RNA sequencing (TCGA, GTEx)
Tumor-specific isoform
RefSeq
Isoform
Isoform-specific reads
* Filtering conditions were described in Methods
B
Isoforms in GENCODE
Isoforms not in GENCODE
Number of junctions
Combination
Unannotated exon
Exon skipping
Exon shuffling
Alternative 5' splice site
Alternative 3' splice site
Lung (GTEx)
Other (GTEx)
Novel
C
LUAD
r = 0.16
LUSC
r = -0.22
COAD
r = -0.22
PAAD
r = -0.11
Number of isoforms
TMB

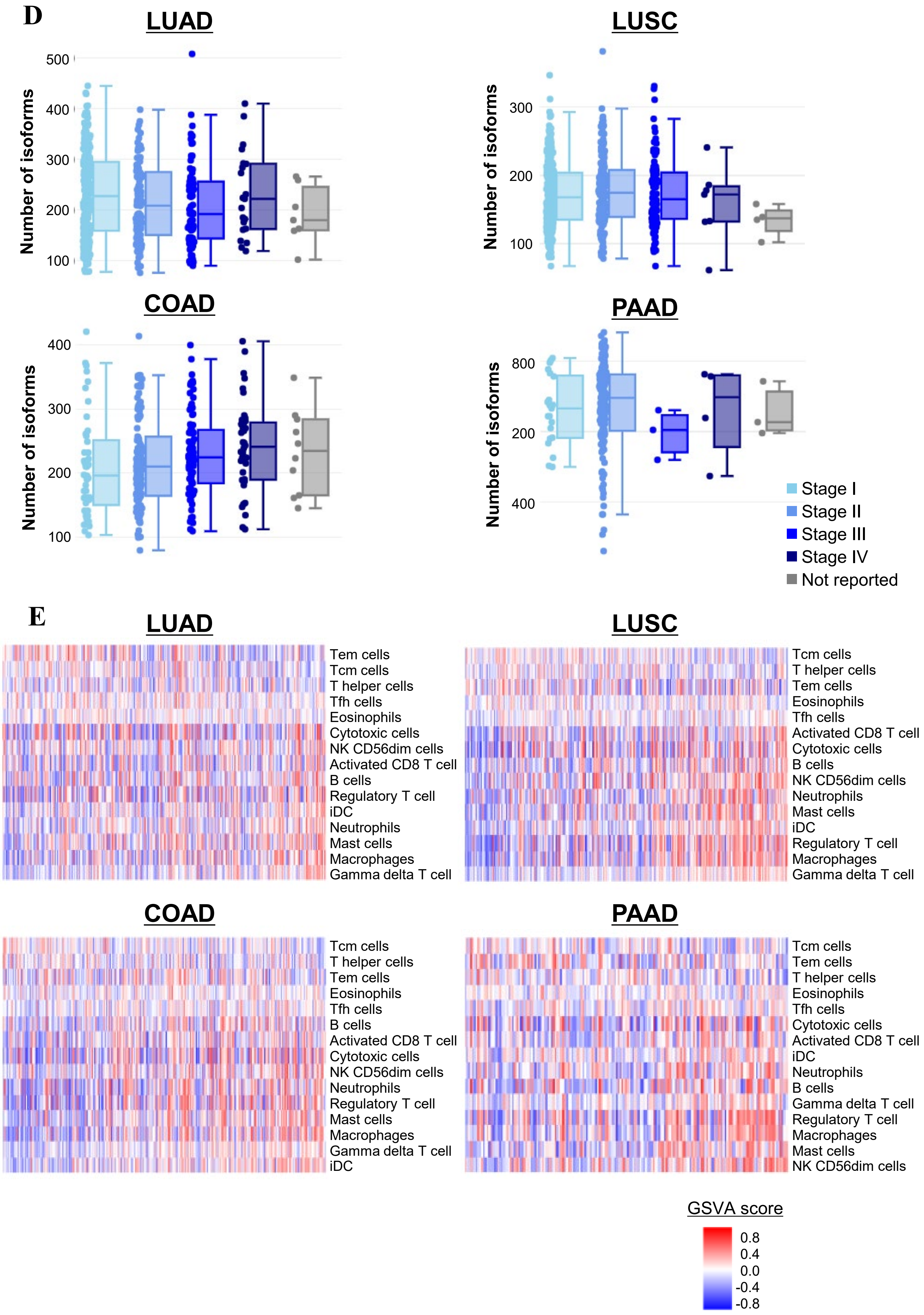
D
LUAD
LUSC
COAD
PAAD
Number of isoforms
500
400
300
200
100
800
Stage I
Stage II
Stage III
Stage IV
Not reported
E
Tem cells
Tcm cells
T helper cells
Tfh cells
Eosinophils
Cytotoxic cells
NK CD56dim cells
Activated CD8 T cell
B cells
Regulatory T cell
iDC
Neutrophils
Mast cells
Macrophages
Gamma delta T cell
GSVA score
0.8
0.4
0.0
-0.4
-0.8

Fig. S20. Overview of analysis using TCGA datasets
(A) The analysis workflow for counting aberrant isoforms in the TCGA datasets using our isoform catalog. Reads from short-read RNA sequencing of TCGA were mapped to the reference genome and our reference catalog using STAR. Reads aligned to isoform-specific regions were counted, and extracted isoforms covered at least 20 reads. A normal reference panel was created by merging normal datasets to extract tumor-specific isoforms. We also removed isoforms whose junctions were expressed in the lung tissues of the GTEx database. (B) The number of junctions found in the GTEx datasets in isoforms that were represented in GENCODE (left panel) and not represented in GENCODE (right panel). (C) Correlation between the number of isoforms and the TMB in the TCGA datasets for lung adenocarcinoma (LUAD), lung squamous cell carcinoma (LUSC), colon adenocarcinoma (COAD) and pancreatic adenocarcinoma (PAAD). No significant correlation was observed. (D) Comparisons of the distribution of the number of isoforms in specimens in each cancer stage. (E) Heatmaps showing the GSVA enrichment scores of gene sets of specific immune cell signatures.
