# Supplementary material for: Aberrant splicing isoforms detected by full-length transcriptome sequencing as transcripts of potential neoantigens in non-small cell lung cancer
Source: Genome Biol. 2021 Jan 4;22:9. doi: 10.1186/s13059-020-02240-8 (PMC7780684; doi:10.1186/s13059-020-02240-8)
Supplement: Supplementary file 15 — Additional file 15. Review history. [file 13059_2020_2240_MOESM15_ESM.docx]

Review History

**First round of review**

**Reviewer 1**

**Are you able to assess all statistics in the manuscript, including the appropriateness of statistical tests used?**

Yes, and I have assessed the statistics in my report.

**Comments to author:**

In this manuscript, the authors applied nanopore long-read sequencing technology to identify novel transcripts in 22 non-small cell lung cancers. The novel transcripts were then classified based on the splicing events, e.g., exon skipping/intron retention/alternative splice sites. The protein expression of these transcripts were confirmed with the proteome analysis. Moreover, the Enzyme-linked immune absorbent spot (ELISPOT) assay analysis showed that half of these peptides interact with HLA molecules and potentially act as an antigen to activate T cells.

By knocking down the nonsense-mediated mRNA decay (NMD) factor, UPF1, and splicing factor, SF3B1, it was concluded that the UPF1- and SF3B1-depleted samples had an increasing number of exon skipping events in their aberrant gene transcripts.

In general, this study sheds a light on the transcript diversities in the cancer cells and also illustrated that UPF1/SF3B1 help in tuning the transcript diversities in the cells. It also indicated that many novel transcripts may act as antigens in cancer cells. However, there are a number of comments/concerns that should be considered:

1-	Considering the main goal of identifying the transcript aberrations in the non-small cell lung cancers, I would suggest to identify the gene-fusion events and their splicing events (including the splices across two genes) as they could be associated with cancer as well.

2-	It was concluded that approximately half peptide candidates (from the translated novel transcripts) act as potential antigens. Please also perform a computational study to predict the potential functions for the other half of novel transcripts with protein products.

3-	Please characterize/identify the sequence motifs and also the nucleotide composition in the 3' UTR (or PTC-containing regions) of the UPF1 targeted mRNA transcripts, i.e., with an aberration after UPF1 knock down, and compare the motifs with the recent studies (e.g. Genome Res. 2017 Mar; 27(3): 407-418.)

4-	Recent studies (e.g., Nature Communications volume 11, Article number: 1438 (2020)) have already confirmed that the SF3B1 mutations are associated with the differential 3' splice sites in the gene transcripts. As you have mainly discussed the exon skipping events in the SF3B1 knock down experiment, I would suggest to investigate the possibilities for the differential 3' splice site events and to compare the result with previous studies.

5-	Please characterize the sequence/structure motifs in the regions that are flanked by the skipped exons, in the SF3B1 knock down, and discuss if they could be binding sites for the SF3B1 or its co-factors.

6-	Do the detected novel splice sites have any preference for a specific repetitive region, e.g., micro-satellites, tandem repeats, LINEs … which could be of biological functions or could cause incorrect alignment (thus false novel splice detection). or they are evenly distributed in all types of regions in the genome.

**Reviewer 2**

**Are you able to assess all statistics in the manuscript, including the appropriateness of statistical tests used?**

Yes, and I have assessed the statistics in my report.

**Comments to author:**

Oka et al presented an analysis of non-ref isoform transcripts in the tumor samples, which are potentially caused by abnormal nonsense mediated decay, and may produce novel neoantigens. This work is conceptually novel, and has the potential to locate new immunotherapy targets, or to provide new prognostic biomarkers for checkpoint blockade therapies. The combined use of long and short read sequencing technologies is a highlight of this work, which reduced the ambiguity in calling multiple isoforms, and provided the benchmark for the analysis of the TCGA data. However, although the methodology is relatively strong, most of the conclusions are weak, if useful at all. I have the following comments:

1. The association between the number of isoforms and gene expression level seems to be a discovery bias. One way to figure this out is, to down-sample sequence reads of the higher-expressed genes, to match the TPM of the lowly expressed ones. Then re-run the pipeline, to see if the number of isoforms is still higher.

2. The biggest concern for the neoantigen analysis is lack of proof that these variants are cancer-specific. Except for frameshift insertions/deletions, the other types of isoforms may also present in the normal transcriptome. Their missing in the RefSeq might simply be due to a lack of thorough annotation. To show if this is the case, the authors need to profile the short read sequencing data of some non-cancer samples, preferably the same tissue type from matched samples, and show that these isoforms are strictly missing in these controls. GTEx might be a good option here. Otherwise, it is not possible to determine if or what fraction of these aberrant isoforms are truly cancer-specific, instead of being cancer-associated (meaning that they are less expressed in the normal tissues, but still transcripts of some normal biological process).

3. The ELISPOT assay simply proved that 8 of 17 peptides were predicted correctly by NetMHC. Since they are 'foreign' to mice, it is almost sure that repeated vaccination will result in CD8+ T cell response. So interferon gamma response doesn't mean antigenicity. The authors should do, or at least discuss some useful experiments that could lead to meaningful conclusions in humans.

4. TCGA analysis: the fact that for most cancer types, NMD deficiency was not correlated with aberrant isoforms, implies that these isoforms may not be truly aberrant. The way the authors defined cancer-specific isoforms is problematic: for TCGA data, it is known that control samples are sequenced at lower depths (about half) than the tumor samples. Therefore, it is expected that most isoforms will be observed to have 2-3 fold increase in the tumor. The authors should define cancer-specific isoforms more strictly, and perform the downstream analysis again. With that, the authors should investigate a broader spectrum of factors, such as the survival associations, immune infiltration levels, cancer grade/stage, etc, instead of TMB only. Such analysis may lead to more useful conclusions.

5. Sequencing data for lung cancer cell lines should be made available to all researchers without controlled access. Also, the predicted isoform information of the TCGA data (as well as other public cohorts to be analyzed by the authors) should be provided along with this manuscript.

**Authors Response**

**Point-by-point responses to the reviewers’ comments:**

Reviewer 1:

In this manuscript, the authors applied nanopore long-read sequencing technology to identify novel transcripts in 22 non-small cell lung cancers. The novel transcripts were then classified based on the splicing events, e.g., exon skipping/intron retention/alternative splice sites. The protein expression of these transcripts were confirmed with the proteome analysis. Moreover, the Enzyme-linked immune absorbent spot (ELISPOT) assay analysis showed that half of these peptides interact with HLA molecules and potentially act as an antigen to activate T cells.

By knocking down the nonsense-mediated mRNA decay (NMD) factor, UPF1, and splicing factor, SF3B1, it was concluded that the UPF1- and SF3B1-depleted samples had an increasing number of exon skipping events in their aberrant gene transcripts.

In general, this study sheds a light on the transcript diversities in the cancer cells and also illustrated that UPF1/SF3B1 help in tuning the transcript diversities in the cells. It also indicated that many novel transcripts may act as antigens in cancer cells. However, there are a number of comments/concerns that should be considered:

1-	Considering the main goal of identifying the transcript aberrations in the non-small cell lung cancers, I would suggest to identify the gene-fusion events and their splicing events (including the splices across two genes) as they could be associated with cancer as well.

*We totally agree with this comment of the reviewer. In fact, the gene-fusion event is reported to play the most important role for tumorigenicity in many cancers, particularly in lung cancers (Mano H et al. 2008 Cancer Sci., Yang W et al. 2019 Nat Med.). To address this issue, we firstly manually inspected the presence or absence of the previously-reported frequently “fused” genes. We successfully identified the EML4-ALK fusion transcript, as a driver fusion event, in H2228. In addition, we attempted to identify previously uncharacterized fusion genes. We identified the ERGIC2-CHRNA6 transcript in H1437. This fusion event is causing the frameshift, invoking a drastic amino acid change, thus may serve as an important source of a novel neoantigen. For these fusions, it is true that our previous study has suggested their presences by the short-read sequencing (Suzuki A et al 2017 DNA Res, Suzuki A et al 2014 NAR). However, in this study, we firstly identified their complete transcript forms by using the MinION long-reads. In fact, by this analysis, we identified the precise fusion junction, which were supported by multiple long-reads. Particularly interestingly for the ERGIC2-CHRNA6 fusion gene, we identified four alternative splice isoforms, consisting of one previously unannotated exon. (Additional file 12: Figs. S4A and B). We added this description in the text (line 200).*

*We further attempted to construct a bioinformatics pipeline which enables such an identification more systematically. To our knowledge, there has not yet been any standard tool for detecting fusion genes from the MinION reads, in spite that several tools are being developed (e.g., AERON; Rautiainen M et al. 2020 bioRxiv). Indeed, we encountered several technically difficulties. It was particularly difficult to avoid the extraction of inaccurate transcript fusions or splicing patterns particularly for the cases spanning homologous genes, due to the error-prone MinION reads. In this particular revision, it was difficult to construct an accurate pipeline to filter out reads that were misaligned to multiple genes. Therefore, in this study, please allow us to focus on the splicing events occurring within the single genes. By this approach, we believe that the constructed catalog of the transcript aberrations should have become much robust.*

2-	It was concluded that approximately half peptide candidates (from the translated novel transcripts) act as potential antigens. Please also perform a computational study to predict the potential functions for the other half of novel transcripts with protein products.

*We thank for this interesting suggestion made by the reviewer. We compared the isoforms used for ELISpot assay with RefSeq transcripts and searched protein domains that were newly generated or disrupted in isoforms. For this purpose, we used the NCBI Batch Web CD-Search Tool (Lu S, et al. 2020 NAR). Although several domains in the isoforms were disrupted due to amino acid change, there was no significant relationship with the result of ELISpot.*

*We were also interested in examining whether some protein domains are characteristic of the group of novel isoforms, or some of which are prone to produce neoantigens. We investigated the protein domains ”which are" newly generated or disrupted in the 3,712 detected isoforms. As a result, only 41 isoforms had newly appeared protein domains and 567 isoforms contained disrupted domains. No domain was significantly enriched in the groups of the isoforms producing neoantigen candidates. We also examined whether the RefSeq genes having a particular domain are likely to produce aberrant isoforms or neoantigen candidates, but no significant enrichment was observed "in this analysis, too". To avoid the confusion, we did not include this information to the text. Please take a look at the Table R1-R4 in “Response Letter”.*

3-	Please characterize/identify the sequence motifs and also the nucleotide composition in the 3' UTR (or PTC-containing regions) of the UPF1 targeted mRNA transcripts, i.e., with an aberration after UPF1 knock down, and compare the motifs with the recent studies (e.g. Genome Res. 2017 Mar; 27(3): 407-418.)

*We appreciate this constructive suggestion. As for four p-UPF1-binding motifs (CCUGGGG, CCUGGGA, CCUGGAA, and CCUGAGA) represented by the indicated paper (Imamachi N et al. 2017 Genome Res), we searched them in the 3'UTR of the isoforms that were detected only in UPF1-knockdown A549 cells. As a result, 34 of the 91 isoforms included the UPF1 motifs. We also evaluated the motif enrichment in UPF1-depleted A549 cells using MEME Suite (Bailey TL et al. 2009 NAR). We found that possible GC-rich motifs were enriched in the 3'UTR of isoforms (Additional file 12: Fig. S12A). Further studies would be necessary to confirm whether they can be actually bound by UPF1. We added these results in the revised manuscript (line 292).*

4-	Recent studies (e.g., Nature Communications volume 11, Article number: 1438 (2020)) have already confirmed that the SF3B1 mutations are associated with the differential 3' splice sites in the gene transcripts. As you have mainly discussed the exon skipping events in the SF3B1 knock down experiment, I would suggest to investigate the possibilities for the differential 3' splice site events and to compare the result with previous studies.

*We thank the reviewer for this important suggestion. We conducted the indicated analysis and discussed the obtained results in the revised manuscript (line 320). The previous study (Tang AD et al. 2020 Nat. Commun.) showed that the K700E hotspot mutation of SF3B1 downregulates intron retention and upregulates alternative 3' splice site events. Consistently in our study, the affected splicing events differed between splicing patterns. Particularly, except for exon skipping, the most affected splicing was intron retention. This result was as expected, since the K700E hotspot is considered to be a gain-of-function variant (Papaemmanuil E et al. 2011 N Engl J Med.) and our knockdown assay of SF3B1 should have an opposite consequence. Alternative 3' splice site events were not significantly affected, which was not always consistent with the previous results, suggesting other cellular contexts also play a role. We presented these results in the Supplementary Figure (Additional file 12: Fig. S12B).*

5-	Please characterize the sequence/structure motifs in the regions that are flanked by the skipped exons, in the SF3B1 knock down, and discuss if they could be binding sites for the SF3B1 or its co-factors.

*We conducted the indicated analysis and added the obtained results in the revised manuscript (line 308). To confirm the consensus sequence in the proximal regions of the splice sites, we collected the exon skipping isoforms that were detected only in the SF3B1-knockdown A549 cells. For this analysis, we considered ±10 bp surrounding regions of the novel splicing junctions and the skipped exons. Although the canonical splice consensus sequences of GT/AG were found in almost all the isoforms similar to the total RefSeq transcripts, no other distinct feature related to the SF3B1 knockdown event was detected. We included these results in Supplementary Figure (Additional file 12: Fig. S12C).*

*We further tried to find a new possible motif in the deep intron regions, which are upstream or downstream of the skipping junctions or exons. Several T-rich or G-rich motifs were detected as well as RefSeq transcripts. T-rich motifs could be polypyrimidine tract (PPT) where U2AF and PPT-binding proteins bind. G-rich motifs may reflect potential binding sites for heterogeneous nuclear ribonucleoprotein regulating alternative splicing. This analysis also needs to be further validated by future experimental assays. We also included these results and the discussion in Supplementary Figure (Additional file 12: Fig. S12D).*

6-	Do the detected novel splice sites have any preference for a specific repetitive region, e.g., micro-satellites, tandem repeats, LINEs … which could be of biological functions or could cause incorrect alignment (thus false novel splice detection). or they are evenly distributed in all types of regions in the genome.

*We thank the reviewer for this insightful comment. To confirm this point, we evaluated the 5,508 splice sites detected in 22 cell lines. We extracted ±50 bp regions around the splicing sites and searched repetitive regions using RepeatMasker. As a result, 94.9% of these splicing sites did not overlap the repetitive sequences (Additional file 12: Fig. S1E)). This result suggests that the novel splice sites were not particularly associated with the repetitive sequences. Also, it was confirmed that those exons were not derived from misalignments and other computational errors. We provided this conclusion in the revised manuscript (line 194).*

Reviewer 2:

Oka et al presented an analysis of non-ref isoform transcripts in the tumor samples, which are potentially caused by abnormal nonsense mediated decay, and may produce novel neoantigens. This work is conceptually novel, and has the potential to locate new immunotherapy targets, or to provide new prognostic biomarkers for checkpoint blockade therapies. The combined use of long and short read sequencing technologies is a highlight of this work, which reduced the ambiguity in calling multiple isoforms, and provided the benchmark for the analysis of the TCGA data. However, although the methodology is relatively strong, most of the conclusions are weak, if useful at all. I have the following comments:

1. The association between the number of isoforms and gene expression level seems to be a discovery bias. One way to figure this out is, to down-sample sequence reads of the higher-expressed genes, to match the TPM of the lowly expressed ones. Then re-run the pipeline, to see if the number of isoforms is still higher.

*As suggested by the reviewer, we subsampled sequencing reads of VMRC-LCD cells to 1/2, 1/5, 1/10, 1/50, and 1/100 (n=100) and calculated the detection probability for each isoform (Additional file 12: Figs. S9B-D and Fig. R1 below). To examine the relation between the expression levels and the detection probability, we divided the isoforms into three classes, High, Middle, and Low (their respective ranges are shown in the margins of Fig. R1), for each of the three categories as below:*

*(1) TPM of the gene (total gene expression), which was calculated from the short-read RNA sequencing data (Additional file 12 Fig. S9B and Fig. R1).*

*(2) Isoform-reads ratio (isoform frequency within the gene): [the number of MinION reads covering the isoform pattern] / [the total number of MinION reads assigned to the gene] (Additional file 12 Fig. S9C and Fig. R1)*

*(3) Isoform-reads (absolute count of the isoform): the number of MinION reads covering the isoform pattern (Additional file 12 Fig. S9D and Fig. R1).*

*As a result, we found that three classes of the isoforms showed similar detection probability in the categories of “TPM” and “isoform reads ratio.”, suggesting that the “isoform” detection was somewhat independent of these categories. On the other hand, “isoform-reads” showed significant differences among the classes for the detection probabilities, suggesting the low-level isoforms were, indeed, difficult to be detected at low sequencing depth. However, at the sequencing depth conducted in this study, each case seems to have reached to a plateau to some extent.*

*Also note that, it is reported, on average, 360,000 mRNA molecules are estimated to exist in a single lung cancer cell (Cui Y et al. 2015 Wiley Interdiscip Rev Nanomed Nanobiotechnol). Thus, one copy of mRNA per cell corresponds to 3 TPM. The minimum TPM of the gene for which at least one isoform was detected was 6 TPM in VMRC-LCD. These facts suggest that we were able to identify the isoforms to the very low expression level within a cell. We added these results in the revised manuscript (line 230).*

2. The biggest concern for the neoantigen analysis is lack of proof that these variants are cancer-specific. Except for frameshift insertions/deletions, the other types of isoforms may also present in the normal transcriptome. Their missing in the RefSeq might simply be due to a lack of thorough annotation. To show if this is the case, the authors need to profile the short read sequencing data of some non-cancer samples, preferably the same tissue type from matched samples, and show that these isoforms are strictly missing in these controls. GTEx might be a good option here. Otherwise, it is not possible to determine if or what fraction of these aberrant isoforms are truly cancer-specific, instead of being cancer-associated (meaning that they are less expressed in the normal tissues, but still transcripts of some normal biological process).

*We thank the reviewer for this precise suggestion. We downloaded the exon-exon junction reads from GTEx. We considered all the data from lung as well as the other tissues. Using the collected GTEx data, we compared their junction patterns with the isoform junctions in this study (see the new section, "Computational analysis of GTEx data", in the MATERIAL AND METHODS section). When we compared them with the novel splice junctions detected in our analysis (line 439), we found only 12.5% of the junctions (neither RefSeq nor GENCODE) were represented in the lung specimens in the GTEx database. Fifty-two percent of the junctions were novel even in all tissues taken together in GTEx (Additional file 12: Fig. S20B; Fig R4). On the other hand, most of the isoforms which had overlapped with GENCODE, thus, had been filtered out from our neoantigen evaluations, were also represented in GTEx in lung or other tissues. To very carefully evaluate the effect of those isoforms represented by GENCODE or GTEx, we removed those isoforms from the TCGA-related analyses (Additional file 12: Fig. S20A). We recalculated all the cases and confirmed the results were essentially the same (Figs. 7A-D, upper left panels).*

*Also note that, since GTEx is based on short-read RNA sequencing datasets, the representation of the junctions in GTEx do not necessarily indicate the existence of the isoform in their full-length forms in normal tissues. To calculate and extract truly cancer-specific isoform patterns, we should generate a larger dataset of long-read isoforms for normal tissues in future study. We included this discussion in the text (line 501).*

3. The ELISPOT assay simply proved that 8 of 17 peptides were predicted correctly by NetMHC. Since they are 'foreign' to mice, it is almost sure that repeated vaccination will result in CD8+ T cell response. So interferon gamma response doesn't mean antigenicity. The authors should do, or at least discuss some useful experiments that could lead to meaningful conclusions in humans.

*We appreciate receiving this important comment of the reviewer. According to the suggestion, we carefully removed isoforms whose junctions were also represented in GTEx. We recalculated the number of isoforms in each specimen, accordingly (Additional file 12: Fig. S20A and line 434). This did not change our conclusion; the number of isoforms and damaging profiles of the NMD factors were significantly associated only in LUAD (p=0.004, Fig. 7A, upper left panel).*

*We also conducted the indicated extensive analyses on the relation between the presence of isoforms and the cancer-related factors. Interestingly, we found positive correlations between the number of isoforms and splicing related signature scores for all cancer types (Fig. 7A-D and line 461). These results suggest that the upregulation of such splicing related genes may contribute to the increase in the number of aberrant isoforms. Otherwise, there was no significant relationship between the number of isoforms and cancer stages or specific immune cell signatures for each cancer type (Additional file 12: Fig. S20D and E, and line 453). Since the clinical specimens we analyzed did not have any immune status information, such as the proportion of TILs or the response of immune checkpoint inhibitor, we were not able to deepen the analysis regarding these factors. Further study is necessary to conclude the effect of these aberrant isoforms for immune systems in tumors. We included this discussion to the text (line 469).*

4. TCGA analysis: the fact that for most cancer types, NMD deficiency was not correlated with aberrant isoforms, implies that these isoforms may not be truly aberrant. The way the authors defined cancer-specific isoforms is problematic: for TCGA data, it is known that control samples are sequenced at lower depths (about half) than the tumor samples. Therefore, it is expected that most isoforms will be observed to have 2-3 fold increase in the tumor. The authors should define cancer-specific isoforms more strictly, and perform the downstream analysis again. With that, the authors should investigate a broader spectrum of factors, such as the survival associations, immune infiltration levels, cancer grade/stage, etc, instead of TMB only. Such analysis may lead to more useful conclusions.

*We appreciate receiving this important comment of the reviewer. According to the suggestion, we carefully removed isoforms whose junctions were also represented in GTEx. We recalculated the number of isoforms in each specimen, accordingly (Additional file 12: Fig. S20A and line 434). This did not change our conclusion; the number of isoforms and damaging profiles of the NMD factors were significantly associated only in LUAD (p=0.004, Fig. 7A, upper left panel).*

*We also conducted the indicated extensive analyses on the relation between the presence of isoforms and the cancer-related factors. Interestingly, we found positive correlations between the number of isoforms and splicing related signature scores for all cancer types (Fig. 7A-D and line 461). These results suggest that the upregulation of such splicing related genes may contribute to the increase in the number of aberrant isoforms. Otherwise, there was no significant relationship between the number of isoforms and cancer stages or specific immune cell signatures for each cancer type (Additional file 12: Fig. S20D and E, and line 453). Since the clinical specimens we analyzed did not have any immune status information, such as the proportion of TILs or the response of immune checkpoint inhibitor, we were not able to deepen the analysis regarding these factors. Further study is necessary to conclude the effect of these aberrant isoforms for immune systems in tumors. We included this discussion to the text (line 469).*

5. Sequencing data for lung cancer cell lines should be made available to all researchers without controlled access. Also, the predicted isoform information of the TCGA data (as well as other public cohorts to be analyzed by the authors) should be provided along with this manuscript.

*We are sorry for the confusion caused by the original version of the manuscript. We deposited sequencing datasets of cell lines into the DNA Data Bank of Japan (DDBJ) under the open access and clinical samples in the DDBJ Japanese Genotype-phenotype Archive (JGA) under the controlled access. We corrected the sentence in the Availability of data and materials in DECLARATIONS section (line 814) as below:*

*The sequencing data of cell lines have been published in the DNA Data Bank of Japan (DDBJ) under accession numbers DRA010214 and DRA010215.*

*Regarding the analysis of the TCGA datasets, we utilized our isoform catalog constructed from 22 LUAD cell lines and seven clinical specimens. We attached this catalog as Additional file 13 and Additional file 14.*

**Second round of review**

**Reviewer 1**

The authors have addressed the concerns. To publish the paper, a few minor grammatical correction to be made:

Line 466: “may contributes”

Line 668: “with a > 80% to repetitive elements”

**Reviewer 2**

The manuscript is significantly improved with GTEx analysis. I don't have further comments.
